# MAO- and Borate-Free Activating Supports for Group 4 Metallocene and Post-Metallocene Catalysts of α-Olefin Polymerization and Oligomerization

**DOI:** 10.3390/polym15143095

**Published:** 2023-07-19

**Authors:** Ilya E. Nifant’ev, Pavel D. Komarov, Oksana D. Kostomarova, Nikolay A. Kolosov, Pavel V. Ivchenko

**Affiliations:** 1 A.V. Topchiev Institute of Petrochemical Synthesis, Russian Academy of Sciences, Leninsky Av. 29, 119991 Moscow, Russia; ilnif@yahoo.com (I.E.N.); komarrikov@yandex.ru (P.D.K.); 2 Chemistry Department, M.V. Lomonosov Moscow State University, Leninskie Gory 1-3, 119991 Moscow, Russia; 3 NIOST LLC, Kuzovlevsky Tr. 2-270, 634067 Tomsk, Russia; kostomarovaod@niost.sibur.ru (O.D.K.); kolosovna@niost.sibur.ru (N.A.K.)

**Keywords:** activating support, alumina, MAO-free, metallocenes, polyethylene, polyolefins, post-metallocenes, silica, supported catalysts

## Abstract

Modern industry of advanced polyolefins extensively uses Group 4 metallocene and post-metallocene catalysts. High-throughput polyolefin technologies demand the use of heterogeneous catalysts with a given particle size and morphology, high thermal stability, and controlled productivity. Conventional Group 4 metal single-site heterogeneous catalysts require the use of high-cost methylalumoxane (MAO) or perfluoroaryl borate activators. However, a number of inorganic phases, containing highly acidic Lewis and Brønsted sites, are able to activate Group 4 metal pre-catalysts using low-cost and affordable alkylaluminums. In the present review, we gathered comprehensive information on MAO- and borate-free activating supports of different types and discussed the surface nature and chemistry of these phases, examples of their use in the polymerization of ethylene and α-olefins, and prospects of the further development for applications in the polyolefin industry.

## 1. Introduction

**Scheme 1:**
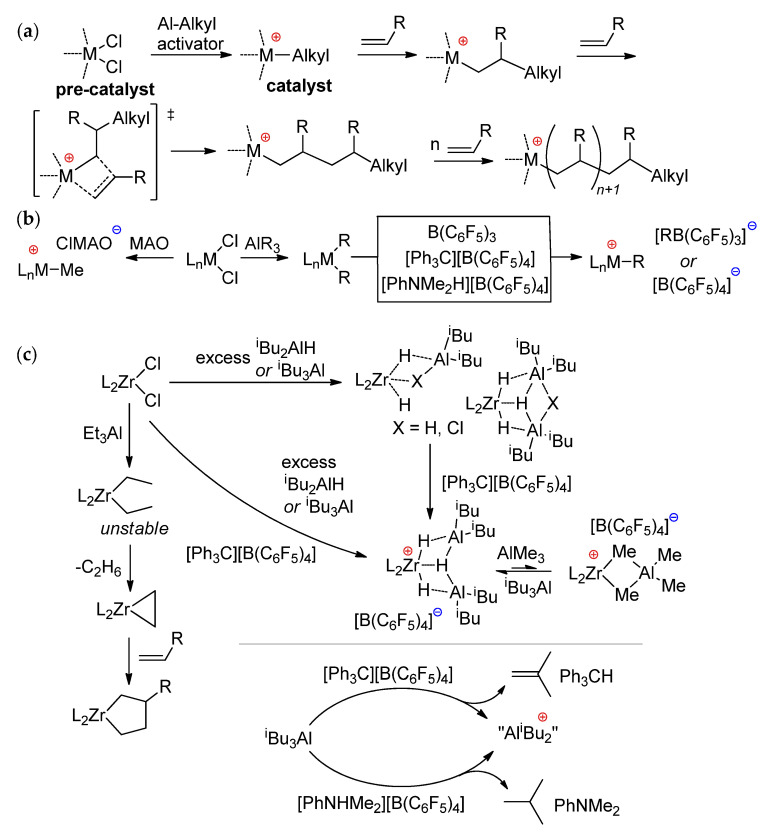
(**a**) Polymerization of α-olefins on Group 4 metal single-site catalysts; (**b**) activation of pre-catalysts; (**c**) neutral and cationic species formed during two-stage activation of zirconocenes by R_3_Al and perfluoroaryl borates. L_2_—bis(η^5^-ligand), ≠—transition state.

Polyolefins still remain the most valuable plastics, with an annual production of ~200 Mt [1]. Most polyolefins are manufactured using Ziegler-Natta titanium catalysts and Phillips chromium catalysts [2,3,4,5,6]. Group 4 metal single-site metallocene and post-metallocene catalysts outperform industrial catalysts on the parameters of activity and homogeneity of ethylene/α-olefin copolymers [2,3,4,7,8]. The advantages of single-site Group 4 catalysts can be primarily attributed to the nature of the catalytic species that represent cationic alkyl-metal centers in basic ligand environments, which have a high ability to π-complexation and insertion of α-olefin molecule [9] (Scheme 1a). Active sites of highly efficient Group 4 metal catalysts are stabilized by weakly coordinating anions. These anions are formed during the activation of pre-catalysts by methylalumoxane (MAO) [10,11] or by trialkylaluminums in combination with B(C_6_F_5_)_3_ or perfluoroaryl borates [9,11,12,13] (Scheme 1b) and other, less commonly used, reagents [14,15,16,17]. High catalytic performance of single-site Group 4 metal catalysts is usually observed when using 10^3^–10^4^ molar equivalents of MAO; perfluoroarylborates can be applied in equal amounts [11,18]. Among trialkylaluminums, Me_3_Al can act as catalyst poison, especially in the case of zirconocenes, due to the formation of dormant L_2_Zr-(μ-Me)_2_-AlMe_2_^+^ species [19,20,21], and one of the functions of excess of MAO under homogeneous conditions is to be a ‘Me_3_Al sponge’. When using zirconocenes and perfluoroaryl borates, activation mechanisms and the structure of catalytic species essentially depend on the type of R_3_Al. In particular, pre-activation of LMCl_2_ by ^i^Bu_3_Al results in the formation of heterometallic hydrides [22,23,24,25], which form cationic species during reaction with borates [26,27]; in the case of Et_3_Al, metallacycles can be formed [28,29] (Scheme 1c). The type of borate activator can also have an impact on the activity, e.g., activation by [PhNMe_2_H][B(C_6_F_5_)_4_] is followed by the formation of PhNMe_2_ capable of coordination at the catalytic center [20]. An important aspect of homogeneous ^i^Bu_3_Al/perfluoroaryl borate activation is the reaction of ^i^Bu_3_Al with borate. As early as 1998, catalyst deactivation caused by the reaction of [CPh_3_][B(C_6_F_5_)_4_] with ^i^Bu_3_Al was observed [30]; this process leads to CPh_3_H, isobutylene, and highly reactive ^i^Bu_2_Al^+^ [31]. When using ^i^Bu_3_Al/[PhNHMe_2_][B(C_6_F_5_)_4_], isobutane was detected in reaction products [32]. Therefore, the mechanism of activation by ^i^Bu_3_Al requires considering the direct participance of cationic ^i^Bu_2_Al^+^ species, which were isolated and characterized recently [33].

**Scheme 2:**
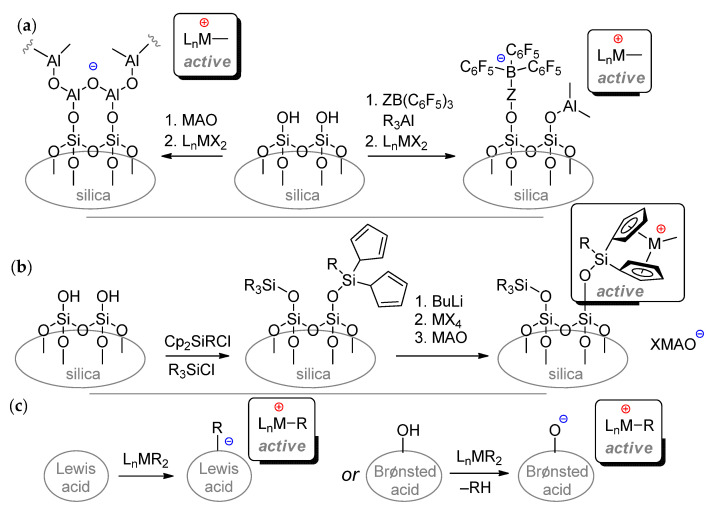
Heterogenization of Group 4 single-site catalysts: (**a**) using silica/MAO (or perfluoroaryl borate) supports; (**b**) via immobilization of the ligand precursors; and (**c**) using activating supports.

The use of a large excess of organoaluminum compounds often leads to hydroalumination [34,35], formation of inactive Zr–CH_2_CH_2_–Al species [36] (^i^Bu_3_Al), and cycloalumination (Et_3_Al) [37,38] side processes. The problems of side reactions during homogeneous activation can be resolved during the adaptation of Group 4 catalysts to actual polyolefin technologies that are tuned for use in gas-phase or slurry processes and require supported catalysts [39]. Heterogenization of single-site catalysts was and is a crucial challenge for the polyolefin industry, and this problem was intensively studied over the past three decades [39,40,41,42,43]. The most evident way to solve this problem is based on the reaction of MAO with active support (silica, alumina, etc.) [44,45,46,47,48,49,50,51] or on functionalization of the support by perfluoroaryl borate or aluminate fragments [52,53,54] with a formation of negatively charged particles that provide binding with catalytically active cationic species through Coulomb attraction (Scheme 2a); stabilization of the catalyst occurs through the same mechanism as for homogeneous catalysts [39,40,44]. The studies of immobilization by covalent binding of the ligand precursors with the support surface (Scheme 2b) were also carried out with varying results, this type of heterogenization also requires MAO or borate activators [39,55,56,57,58,59]. Finally, a number of research groups investigated acidic supports that can react with Group 4 metal complexes with a formation of active cationic catalytic species (Scheme 2c) [46,60]. Activating supports are particularly attractive since they do not require MAO or borates (only R_3_Al are needed as alkylating agents and scavengers), thereby reducing the overall cost of the catalysts.

**Scheme 3:**
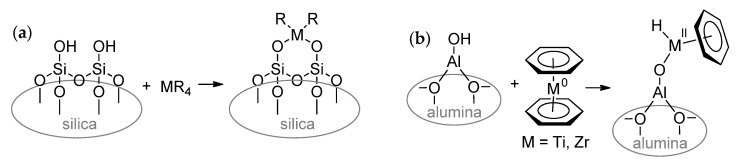
Early studies: activation of Group 4 metal alkyls (**a**) and bis(arene) complexes (**b**) by silica and alumina.

The very idea of the activating supports for Group 4 metallocenes and post-metallocenes is based on early studies of the activation of homoleptic alkyl derivatives of Ti(IV) and Zr(IV) by Al_2_O_3_ and SiO_2_; the activating effect of metal oxides was attributed to the reaction of surface–OH groups of the support (*S*) with MR_4_ that resulted in the formation of (*S*)(–O)_2_MR_2_ species (Scheme 3a) [61,62,63,64,65,66]. Note that the chemical nature of catalytically active (≡Si–O)_2_ZrBn_2_ species, formed on SiO_2_ surface, was confirmed experimentally just in 2013 [67]. Activation of bis(arene) Ti(0) and Zr(0) complexes by Al_2_O_3_ presumably proceeds via oxidative addition of a surface O–H bond to give a divalent supported species (Scheme 3b) [65,66,67,68,69].

The first experimental proof of the ability of support to activate metallocenes with a formation of highly active cationic catalytic species according to Scheme 2c comes from the studies of Marks and colleagues [70,71,72]. In 1985, they investigated the reaction of (η^5^-C_5_Me_5_)_2_ThMe_2_ with dehydroxylated γ-Al_2_O_3_ (DA); the transfer of methyl groups from Th to surface Al sites with a formation of cationic species was detected using ^13^C cross-polarization/magic angle spinning (CP MAS) NMR [70]. In 1988, they showed that DA is an efficient activator for (η^5^-C_5_H_5_)_2_ZrMe_2_, (η^5^-C_5_Me_5_)_2_ZrMe_2_, and (η^5^-C_5_Me_5_)ZrMe_3_ in ethylene polymerization [71]; Zr→Al migrations of Me group were also confirmed by ^13^C CP/MAS NMR experiments [65,69].

In the early 1990s, Soga and Kaminaka [73,74,75] studied the propylene polymerization activity of different zirconocene dichlorides activated by AlR_3_, with SiO_2_, Al_2_O_3_, MgCl_2_, MgF_2_, CaF_2_, or AlF_3_ supports. The absence of activity, observed for SiO_2_ and MgO, was attributed to a lack of acidity of these oxides compared with the other materials. MgCl_2_ was then studied as an activating support for Ti(IV)-based post-metallocenes [76,77,78,79] and zirconocenes [73,80,81]. Since unmodified silica and alumina provided only partial activation of metal complexes [82], in the current century, the focus was on sulfated metal oxides, studied mainly by the scientific group of Marks [83,84,85,86,87], and fluorinated metal oxides, studied by the scientists from Elf [88], Chevron Phillips [89,90,91,92,93,94,95,96,97,98], INEOS [99,100,101], and Total in cooperation with the scientific group of Boisson [102,103,104,105,106,107].

The use of activating supports in the catalytic chemistry of α-olefins has been discussed in a number of review papers and book chapters. The first promising results in the surface chemistry of sandwich complexes of actinides and Group 4 metals have been discussed by Marks and Eisen in the early 1990s [72,108]. Some issues regarding metallocene activation by silica [109], and the difference in activating chemistry and efficiency between silica and alumina [110], were discussed by Copéret et al. The importance of achieving supported catalyst structural uniformity was also demonstrated in a review from Wegener et al [111]. The chapters, devoted to MgCl_2_-supported single-site catalysts [112] and activation of metallocenes by solid acids [113], were published in the collection ‘*Tailor-Made Polymers, Via Immobilization of Alpha-Olefin Polymerization Catalysts*’ (2008). In 2018, activating supports for metallocenes and related catalysts were briefly discussed by Hoff in a chapter of the ‘*Handbook of Transition Metal Polymerization Catalysts*’ [114]. Activating supports were also mentioned in recent reviews on supported catalysts [41,42,43,46,60].

In the present work, we summarized and analyzed published data on activating supports of different types and compared mechanisms and efficiency of Group 4 metallocene and post-metallocene activation and reactivity. The prospects of the use of activating supports in the polyolefin industry are also discussed.

## 2. Metal Halides

### 2.1. MgCl_2_

**Figure 1:**
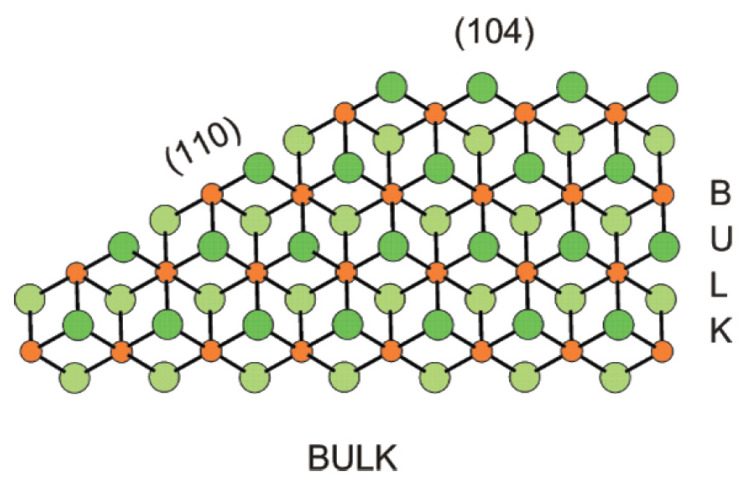
Schematic representation of a MgCl_2_ monolayer fragment developed by Cavallo et al. The (104) and (110) lateral faces are indicated. The Mg atoms are colored orange. The Cl atoms above and below the Mg plane are dark and light green colored, respectively. Reprinted with permission from [116]. Copyright (2012) American Chemical Society.

MgCl_2_ is a widely used support for conventional titanium–magnesium Ziegler–Natta catalysts (TMZNCs) of third and subsequent generations [115,116,117,118,119,120]. In view of the industrial importance of TMZNCs, efficient methods for controlling the particle size, porosity, and morphology of MgCl_2_ have been developed, and it is not surprising that MgCl_2_ was studied as a support for Group 4 metal single-site catalysts [39,112]. MgCl_2_ supports may also have the advantage of easier fragmentation than silica or alumina supports, thereby facilitating polymerization and improving the morphology of polymer particles formed. Whilst TMZNCs are not the subject of our review, the role of MgCl_2_ as an activating support for absorbed Ti catalytic species is not in doubt. According to current theoretical concepts about the nature of TMZNCs, pre-catalysts represent TiCl_4_ adsorbed on MgCl_2_ crystallites, and binding strength depends on the crystal structure of the MgCl_2_ support. In their fundamental works, Cavallo and colleagues assumed the MgCl_2_ bulk to be in the α crystalline phase with the surface comprising (104) and (110) lateral cuts that have Mg centers with coordination numbers (CN_Mg_) of 5 and 4, respectively (Figure 1) [116,121].

In terms of the reaction mechanism, the method of the activation, i.e., the introduction of R_3_Al after or before/simultaneously with the interaction of the pre-catalyst with MgCl_2_, could be essential. In the latter case, the chemistry of R_3_Al on the MgCl_2_ surface is an important point in examining the activation of Group 4 metal complexes. Using ^27^Al MAS NMR spectroscopy, Potapov and colleagues have studied organoaluminum compounds Et_n_AlCl_3−n_ supported on a highly dispersed MgCl_2_, prepared by the reaction of Mg with *n*-BuCl [122]. In the ^27^Al MAS NMR spectrum of the sample Et_2_AlCl/MgCl_2_ they detected a characteristic signal of Al species with coordination number (CN_Al_) = 5. An attempt to record the ^27^Al MAS NMR spectrum of Et_3_Al/MgCl_2_ failed; however, analysis of the spectrum obtained after treatment with Bu_2_O allowed to propose the formation of dimeric Et_2_Al(μ-Et)_2_AlEt_2_ species on the MgCl_2_ surface (CN_Al_ = 5). However, the reaction of MgCl_2_ with R_3_Al can not be called fully studied; a lot of research on TMC activation was focused on R_3_Al interactions with Ti species [123,124] without analyzing concurrent processes on the MgCl_2_ surface. 

In the 1990s, a number of MgCl_2_-supported Group 4 metal complexes were studied in polymerization experiments after activation by alkylaluminum compounds. Kaminaka and Soga activated *rac*-[1,2-ethylenebis(η^5^-4,5,6,7-tetrahydroinden-1-yl)]ZrCl_2_ (*rac*-[EBTHI]ZrCl_2_, **Zr1**) by R_3_Al (R = Me, Et) in the presence of MgCl_2_ [73]. The supported catalyst was prepared by the grounding of the support and **Zr1** in toluene in the presence of R_3_Al. In propylene polymerization, supported catalysts were significantly inferior to homogeneous **Zr1**/MAO by activity, but at the same time, polypropylene (PP), formed when using a supported catalyst, had higher isotacticity. During a comparative study of (η^5^-C_5_H_5_)_2_ZrCl_2_ (Cp_2_ZrCl_2_, **Zr2**) and [Me_2_C(η^5^-fluoren-9-yl)(η^5^-C_5_H_4_)]ZrCl_2_ (Me_2_C(Flu)(Cp)ZrCl_2_, **Zr3**), activated by Me_3_Al in the presence of commercial MgCl_2_, atactic and syndiotactic polypropylenes were obtained; both zirconocenes have demonstrated high activities comparable with the activity of MAO-activated complexes [74]. In further studies, the mechanism of the activating of Cp_2_ZrX_2_ (X = Cl, Me, Et) by Zr→Mg transfer of X with a formation of Cp_2_ZrX^+^ was proposed [75]. Note that with an increase of the Al:Zr ratio, catalytic activity goes through the maximum; this was attributed to R_3_Al binding with the MgCl_2_ surface. The binding of Cp_2_Zr fragments with MgCl_2_ surface is rather weak: so, for example, Ochędzan-Siodłak and Nowakowska showed that the addition of MAO to the **Zr2**/MgCl_2_/R_3_Al system results in the formation of a homogeneous catalyst [125].

Soga and colleagues have studied the catalytic behavior of CpTiCl_3_ (**Ti1**), supported on MgCl_2_ and activated by ^i^Bu_3_Al, in the polymerization of propylene [126]. Surprisingly, when using a MgCl_2_-supported catalyst, isotactic PP was obtained. However, in a later study, Kang and colleagues reported a lack of activity when using CpMCl_3_/MgCl_2_ (M = Ti, Zr) pre-catalysts prepared in the presence of 2-ethylhexanol [127].

Bis- and mono-cyclopentadienyl Ti(IV) complexes Cp_2_TiCl_2_ (**Ti2**) and **Ti1** were supported on MgCl_2_ by physical (grinding) or chemical (in the presence of Et_3_Al) methods [128]. UV-vis spectra showed coordination of Cl ligands of **Ti1** and **Ti2** to surface Mg Lewis acidic sites (LAS), which were more pronounced for **Ti1**. After activation by Et_3_Al or MAO, supported catalysts were studied in propylene polymerization. For both Ti complexes, isotactic PP was obtained, and the authors attributed this fact to the formation of Ti(III) catalytic species.

**Scheme 4:**
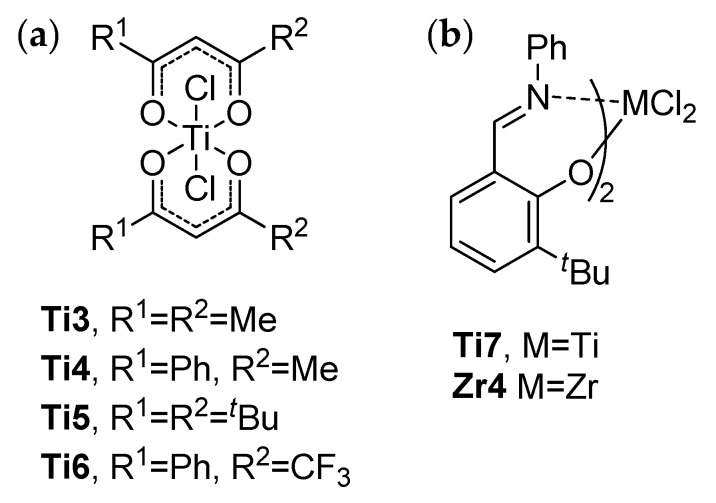
(**a**) β-Diketonate Ti(IV) complexes studied in propylene polymerization after activation by R_3_Al/MgCl_2_ [129]; (**b**) FI catalysts studied in ethylene polymerization using Et_3_Al/MgCl_2_ activator [79].

The first study of the activation of Group 4 post-metallocenes using MgCl_2_ and different alkylaluminums in propylene polymerization was conducted by Soga and colleagues in 1997 [129] on the example of β-diketonate L_2_TiCl_2_ complexes **Ti3**–**Ti6** (Scheme 4a). Pre-catalysts were prepared by the reaction of **Ti3**–**Ti6** with MgCl_2_ in toluene, and the activation by alkylaluminums was performed in *n*-heptane. The polymerization rate when using **Ti3** decreased in the following order: MAO > Me_3_Al > Et_3_Al > ^i^Bu_3_Al > Et_2_AlCl from 300 to 17 kg∙mol^−1^∙h^−1^; mainly atactic PP was formed in all experiments. Ethylene/propylene copolymerization on the example of **Ti6** as a pre-catalyst with and without diisopropyldimethoxysilane as an ‘external donor’ was studied the following year [76]. The nature of the catalytic species, formed by **Ti3**–**Ti6** after activation, remained unclear, and the fact that the activating support mechanism is applicable to these catalysts did not.

Bis(phenoxy-imine) Group 4 metal complexes (named FI catalysts), combined with MAO or borate activators, display unique polymerization activities in α-olefin polymerization [130,131,132,133,134,135]. Because of the high productivity of FI catalysts and the broad spectrum of accessible polymers, there has been significant interest in developing supported catalysts for industrial applications. The use of harmless and cost-competitive MgCl_2_ as an activating support seemed a profitable and promising idea. In 2004, the Fujita group reported the results of the study of catalytic behavior of the complexes **Ti7** and **Zr4** (Scheme 4b), activated by Et_3_Al in the absence and in the presence of mechanically pulverized MgCl_2_, in the polymerization of ethylene. At 50 °C and an ethylene pressure of 0.9 MPa, no polymer formed in the absence of MgCl_2_, whereas in the presence of MgCl_2_, **Ti7** and **Zr4** demonstrated activities of 0.32 and 9.10 kg∙mmol^−1^∙bar^−1^∙h^−1^ [79]. The authors proposed that MgCl_2_ acts as a Lewis acid to form a cationic active species from the ethylated FI catalysts and becomes an anionic species stabilizing cationic active species. Under identical conditions, metallocenes **Zr2** and **Ti2** provided only a trace amount of PE (<0.1 kg∙mmol^−1^∙bar^−1^∙h^−1^).

### 2.2. MgCl_2_/THF Adducts

Supported Cp_2_TiCl_2_/MgCl_2_ pre-catalyst, prepared by the precipitation of **Ti2** and MgCl_2_ solution in THF into pentane, polymerized ethylene after activation by ^i^Bu_3_Al or Et_2_AlCl, and no activity was detected after treatment of **Ti2** by ^i^Bu_3_Al in the absence of MgCl_2_ [136]. Et_2_AlCl was found to be a more efficient activator in comparison with ^i^Bu_3_Al, and the catalysts showed a steady-state kinetic behavior. The authors explained these results by Ti→Mg transfer of the alkyl group with a formation of active Cp_2_TiR^+^ species.

The MgCl_2_(THF)_2_/Et_2_AlCl phase was proposed as a support for **Zr2** by Nowakowska and colleagues [125,137], who assumed the formation of the catalyst precursor Cp_2_Zr(μ-Cl)(μ-Et)Al(μ-Cl)(μ-THF)Mg on the surface of MgCl_2_(THF)_2_, which was activated by MAO without desorption. Activation of this catalyst by R_3_Al was not studied.

### 2.3. MgCl_2_/ROH Adducts

The products of the reaction of MgCl_2_/ROH adducts with alkylaluminum compounds are another type of MgCl_2_-based activating supports. Evidently, their chemical nature is qualitatively different from the chemical nature of the product of the reaction of MgCl_2_ with R_3_Al. 

**Scheme 5:**
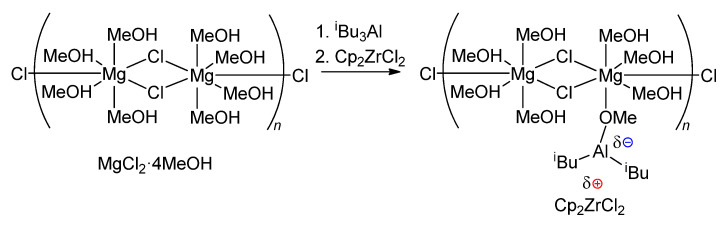
Proposed mechanism for the impregnation of the metallocene catalysts over the recrystallized MgCl_2_ [138].

Cho and Lee prepared adducts MgCl_2_·4MeOH and MgCl_2_·3.33EtOH, and thermal treatment of the former complex resulted in the formation of MgCl_x_(OMe)_y_ species [138]. Active supports were obtained by the treatment of the adducts with Me_3_Al, Et_3_Al, ^i^Bu_3_Al, or MAO (comparative experiments). A more efficient binding with alkylaluminums was observed for MgCl_2_·4MeOH due to the formation of Mg–(μ-OMe)–Al species. The proposed impregnation mechanism for **Zr2**, based on ^27^Al NMR spectral data, is presented in Scheme 5. Supported catalysts were studied in ethylene/hex-1-ene copolymerization after activation by MAO; therefore, the results of the research MgCl_2_(ROH)_x_/R_3_Al system cannot be regarded as a full-fledged activating support. 

**Figure 2:**
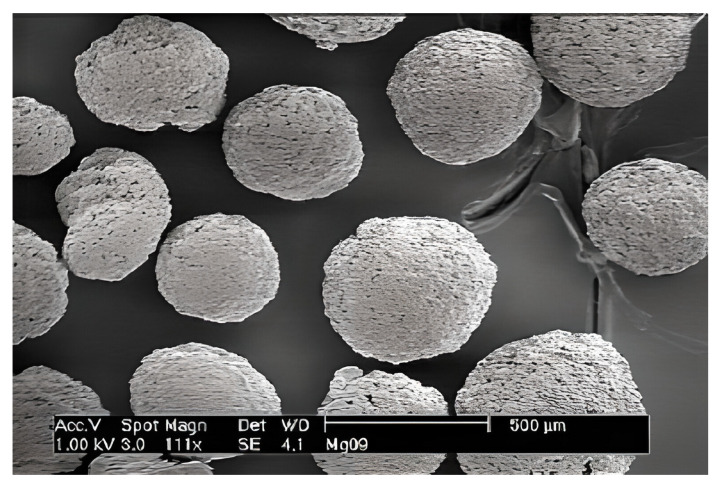
Scanning electron micrograph of polyethylene prepared using Cp_2_TiCl_2_ (**Ti2**)/MgCl_2_ 1.1EtOH/Et_3_Al catalyst. Reprinted with permission from [80]. Copyright (2004) Wiley-VCH Verlag GmbH and Co.

Subsequent studies of the use of MgCl_2_ supports obtained by the reaction of MgCl_2_/EtOH adducts with Al^i^Bu_3_, AlEt_3_, or AlEt_2_Cl have demonstrated high efficiency of ^i^Bu_3_Al and Et_3_Al pre-treated supports in activation of **Ti2**, **Zr2**, and Me_2_Si(η^5^-C_5_Me_4_)(N*^t^*Bu)TiCl_2_ (**Ti8**) in the absence of MAO and borates [80]. In the homopolymerization of ethylene, catalytic activities reached 300 kg∙mol^−1^∙bar^−1^∙h^−1^, and PE of spherical morphology (Figure 2) and relatively high dispersity *Đ*_M_ = 2.4–2.7 were obtained. Note that **Ti2**/MgCl_2_∙2.1EtOH/^i^Bu_3_Al catalyzed formation of PE with *Đ*_M_ = 2.0, which confirms the uniformity and stability of the active species. The activity of *rac*-[1,2-ethylenebis(η^5^-inden-1-yl)]ZrCl_2_ (*rac*-[EBI]ZrCl_2_, **Zr5**)/MgCl_2_∙2.1EtOH/Et_3_Al was 5 kg∙mol^−1^∙bar^−1^∙h^−1^, and PE with narrow molecular weight distribution (*Đ*_M_ = 2.1) was formed. 

Supports, prepared by the reaction of MgCl_2_/EtOH with Et_3_Al (Et_3_Al/EtOH = 2), were used for the activation of a series of zirconocenes and hafnocenes in ethylene polymerization [81]. The investigation of substituent effects was first carried out using the support of composition MgCl_2_∙0.30AlEt_2.46_(OEt)_0.54_ (Table 1). Catalyst immobilization was obtained by contacting the support overnight with a metallocene solution in toluene; ethylene polymerization was carried out at 50 °C with an ethylene pressure of 5 bar. The ethylene mass flow remained essentially constant during polymerization, indicating negligible decay in catalyst activity.

As can be seen in Table 1, low activities were obtained for (RCp)_2_ZrCl_2_ with R = H or Et, but longer-chain alkyl substituents (**Zr7**, **Zr9**) provided much higher activities. Introducing branching into the alkyl substituent (**Zr8**, **Zr10**) led to poor catalyst performance. According to the study of the authors, the increased steric bulk imposed by branched substituents evidently impedes effective activation of the magnesium chloride support, but the causes of abnormal activities of **Zr7** and **Zr8** in comparison with **Zr6** remain unclear. The analogous hafnocenes were also investigated; Cp_2_HfCl_2_ (**Hf1**) demonstrated an activity of 40 kg∙mol^−1^∙bar^−1^∙h^−1^, whereas with (*n*-BuCp)_2_HfCl_2_ (**Hf2**), the activity was 240 kg∙mol^−1^∙bar^−1^∙h^−1^. An increase in the number of Me substituents had no effect on the activity (**Zr13**, **Zr15**–**Zr17**). Mixed-ligand zirconocenes (RCp)(Cp)ZrCl_2_ with R = *n*-Pr, *n*-Bu, and *n*-pentyl (**Zr18**–**Zr20**) demonstrated activities of 740, 1020, and 560 kg∙mol^−1^∙bar^−1^∙h^−1^, respectively, i.e., a smoothed but clear maximum of activity was observed for R = *n*-Bu in (RCp)(Cp)ZrCl_2_ with equally unclear reasons. The variation of the composition of MgCl_2_/EtOH complexes using **Zr7** showed a better performance for support, prepared from MgCl_2_∙1.1EtOH (4300 kg∙mol^−1^∙bar^−1^∙h^−1^), and a higher EtOH content resulted in a lowering of the activity. When ball-milled MgCl_2_ was used as a support, activity was 720 kg∙mol^−1^∙bar^−1^∙h^−1^. When using ^i^Bu_3_Al instead of Et_3_Al, more thermally stable catalysts were obtained, and the activity of the **Zr7**-based catalyst was 12,680 kg∙mol^−1^∙bar^−1^∙h^−1^. It can be assumed that the higher efficiency of ^i^Bu_3_Al in comparison with Et_3_Al is due to the possibility of side reactions after the formation of unstable ZrEt_2_ derivatives (Scheme 1c) [37,38]. Note that the use of (*n*-PrCp)_2_ZrMe_2_ (**Zr7′**) instead of dichloro complex **Zr7** with different organoaluminum co-catalysts did not result in changes of activity and PE characteristics, signifying that the limiting factor in the generation of active species in this system is not the alkylation stage. UV-vis spectral studies showed that the uptake of **Zr2** from the solution was much slower than that of **Zr6** or **Zr7**, indicating a beneficial effect of an electron-donating alkyl substituent. 

**Figure 3:**
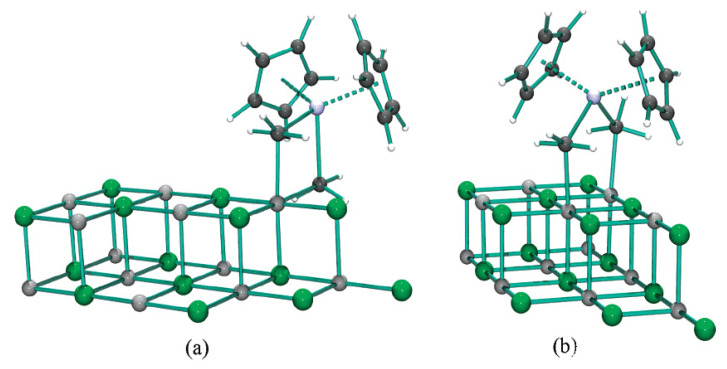
Possible modes of coordination of a zirconocene on (**a**) a (110) and (**b**) a (100) face of MgCl_2_. Reprinted with permission from [81]. Copyright (2008) American Chemical Society.

The nature of the active species in MgCl_2_-supported catalysts is still far from being resolved. Reasoning by analogy with TMZNCs, two possible coordinations were proposed for Cp_2_ZrMe_2_ (**Zr2′**, Figure 3) [81]. The formation of alkyl-zirconocenium cations was attributed to the abstraction of the Me group by acidic Mg centers. Another important factor was the complexation of R_2_AlOEt with **Zr2** or **Zr2′**; supports with relatively low contents of residual ethoxide would therefore be expected to give higher activities for immobilized zirconocenes, as observed in [81]. In our opinion, the coordination of **Zr2′** on the MgCl_2_ surface, presented in Figure 3, is a very rough model: Mg–(μ-OEt)–Al species, formed during the reaction of MgCl_2_/EtOH with R_3_Al, have little choice other than to participate in the formation of active species with **Zr2** or **Zr2′**.

It should also be noted that the reaction of MgCl_2_/EtOH with **Ti2** in the absence and in the presence of R_3_Al is a complex process with a formation of diverse reaction products: as shown in 2016 by Sobota and colleagues [139], during this reaction, **Ti2** loses η^5^-ligands, and heterometallic polynuclear clusters are formed. Given the high nucleophilicity of Mg and Al alkoxides, the decomposition of metallocene fragments is also possible for Zr complexes, but this issue has not yet been studied. 

**Scheme 6:**
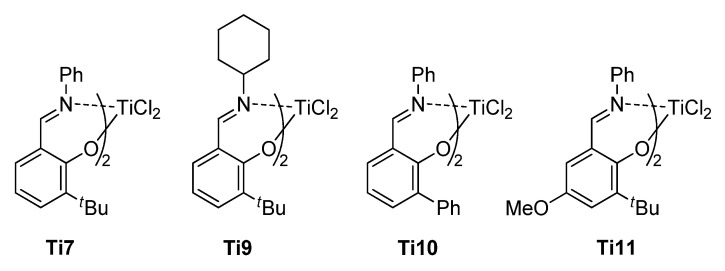
Bis(phenoxy-imine) titanium complexes, studied in ethylene polymerization using MgCl_2_/^i^Bu_m_Al(OR)_n_ support [77,78,79].

**Figure 4:**
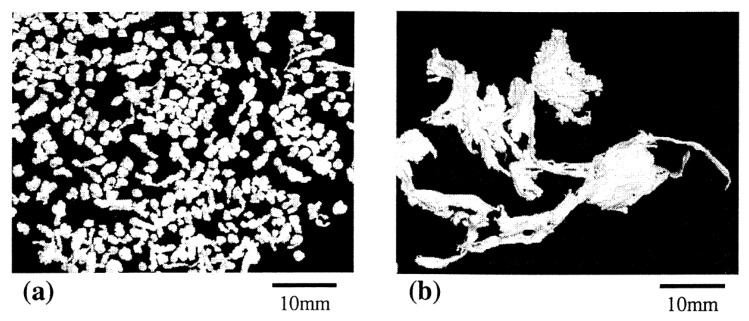
Photographs of the polyethylenes formed with complex **Ti7** using (**a**) MgCl_2_/^i^Bu_m_Al(OR)_n_ or (**b**) MAO as an activator. Reprinted with permission from [78]. Copyright (2003) Elsevier B. V.

The MgCl_2_/ROH/R′_3_Al system was also successfully used for the activation of Group 4 post-metallocenes. Nakayama et al. [77,78,79] prepared an MgCl_2_/2-ethyl-1-hexanol adduct, which is soluble in *n*-decane; treatment of this adduct with ^i^Bu_3_Al resulted in the formation of an MgCl_2_/^i^Bu_m_Al(OR)_n_ support, which is used for activation of bis(phenoxy-imine) titanium complexes **Ti7** and **Ti9**–**Ti11** (Scheme 6). At 50 °C and an ethylene pressure of 0.9 MPa, catalytic activities were 36.3, 20.8, 36.0, and 26.2 kg∙mmol^−1^∙h^−1^, respectively. *M*_w_ of PE was 230–1170 kDa, with broadened molecular weight distribution (*Ð*_M_ = 2.4–3.5). When using MAO as an activator, PE of the worst morphology was obtained (Figure 4).

**Scheme 7:**
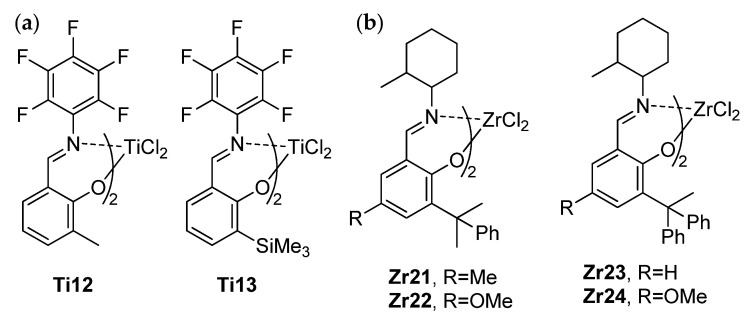
Bis(phenoxy-imine) titanium (**a**) and zirconium (**b**) complexes, studied in polymerization using MgCl_2_/^i^Bu_m_Al(OR)_n_ supports [79,140].

To confirm the single-site nature of the catalyst using an MgCl_2_/^i^Bu_m_Al(OR)_n_ activator, copolymerization of ethylene with propylene was performed [78,79]. Complex **Ti10** produced a copolymer with a propylene content of 29.3 mol% with an activity of 28.2 kg∙mmol^−1^∙h^−1^ and a narrow MWD (*Ð*_M_ = 1.70), thus confirming homogeneity of the catalytic species [78,79]. The studies of **Ti12** and **Ti13**, containing C_6_F_5_ fragment (Scheme 7a), in the polymerization of propylene resulted in the following: **Ti12**-based system catalyzed living propylene polymerization at 25 °C and atmospheric pressure. The living nature was confirmed by the linear relationship between *M*_n_ and polymerization time and by the narrow MWD of the PP obtained (*M*_n_ 53 kDa, *Đ*_M_ = 1.09). The complex **Ti13** activated by MgCl_2_/^i^Bu_m_Al(OR)_n_ produced highly syndiotactic PP ([*rr*] = 97%, *T*_m_ = 155 °C).

When studying bis(phenoxy-imine) Zr complexes **Zr4** (Scheme 4b) and **Zr21**–**Zr24** (Scheme 7b) using MgCl_2_/^i^Bu_m_Al(OR)_n_ activator in ethylene polymerization, a catalytic activity of 1820 kg∙mmol^−1^∙h^−1^ (**Zr22**) was achieved. For **Zr21** and **Zr22**, MAO was a less effective activator in comparison with MgCl_2_/^i^Bu_m_Al(OR)_n_ [79], and PE had a very high molecular weight (up to *M*_v_ = 3 MDa) with an exceptionally high bulk density value of 0.47 g∙mL^−1^ due to excellent spherical morphology of polymer particles.

**Figure 5:**
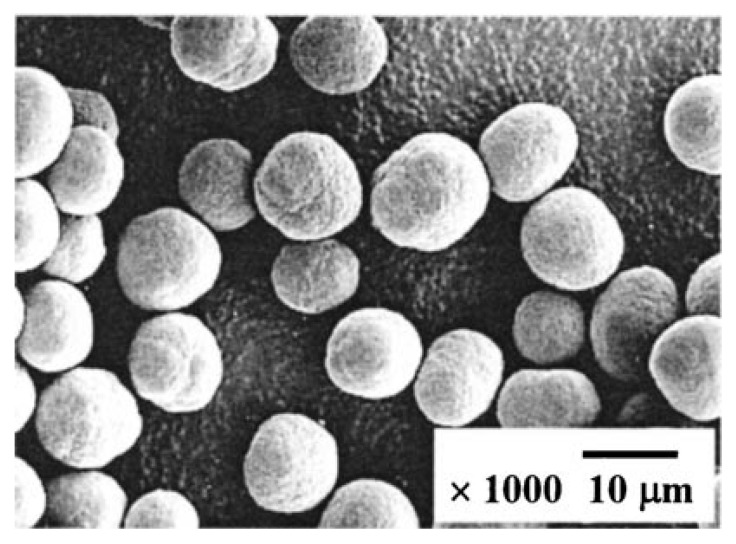
SEM image of the spherical particle PEs produced with MgCl_2_-supported Zr-FI catalyst **Zr23**. Reprinted with permission from [140]. Copyright (2006) Wiley-VCH Verlag GmbH and Co.

The main findings and prospects of FI catalysts when using MgCl_2_/R′_n_Al(OR)_3−n_ activating supports were presented and discussed by Fujita and colleagues in conceptual work [140] that summarized and complemented the results of previous studies [77,78,79]. In the case of FI catalysts, MgCl_2_-based supports pose a real competition to MAO, particularly with regards to polymer morphology (Figure 5). 

### 2.4. MgCl_2_/SiO_2_ Supports

To prepare MgCl_2_/SiO_2_ bisupport particles, Chung and colleagues used agglomeration of silica gel under the action of aq. MgCl_2_ [141]. After separation and drying at 80 °C, bisupport particles were treated with Me_3_Al and **Zr2** in toluene media. The supported catalyst demonstrated an activity of 1013 kg∙mol^−1^∙bar ^−1^∙h^−1^ in the polymerization of ethylene. One might suppose that the organoaluminum activator in this experiment represents MAO, which was formed by hydrolysis of Me_3_Al; however, the comparative experiment with MAO was characterized by a qualitatively different kinetic profile with rapid deactivation of the catalyst.

A prospective approach to MAO- and borate-free supported catalysts was proposed by Kissin and colleagues who prepared catalysts by mixing **Zr5**, Bu_2_Mg, and Et_2_AlCl solutions (Zr/Mg/Al ratio ~1:10:30), followed by addition of the mixture to Davison-grade 955 silica [142]. In ethylene/hex-1-ene copolymerization at 90 °C and ethylene pressure of 1.3 MPa, catalytic activities up to 7800 kg∙mol^−1^∙h^−1^ were achieved, and the hex-1-ene content in the copolymer was 4.4 mol%. The authors assumed that the reaction of Bu_2_Mg with Et_2_AlCl results in the formation of MgCl_2_ that can abstract R^–^ or Cl^–^ from the alkylated Zr complexes, and that the resulting anionic species have a broadly distributed negative charge to stabilize *rac*-[EBI]ZrR^+^ active species.

In 2018, Ko and colleagues described binary support prepared from MgCl_2_/EtOH adducts and silica [143]. Subsequent treatment with various alkylaluminum compounds and **Zr9** resulted in the formation of supported catalysts for ethylene/hex-1-ene copolymerization. Among alkylaluminum compounds, Et_3_Al_2_Cl_3_ demonstrated the highest efficiency, but the hex-1-ene content was ~1 mol% in all experiments. The scheme of catalyst immobilization, presented in [143], looks doubtful as it suggests the formation of a Zr–(μ-O)–Al bond that hinders the formation of active catalytic species. 

### 2.5. Metal Fluorides

Comparative experiments on the activation of **Zr1** by Me_3_Al using MgF_2_, CaF_2_, and AlF_3_, conducted by Soga et al. [75], demonstrated relatively high activities in ethylene polymerization for Group 2 metal fluorides, which were comparable with the activities when using MgCl_2_ support. Similar results were obtained for **Zr3**, which was supported on MgF_2_ in the presence of Me_3_Al [75].

The absence of further research on metal fluoride-activated single-site Group 4 metal can be attributed to the greater development of MgCl_2_-based catalysts, widely used in Ziegler–Natta industrial processes. As a result, we have what we have, and the very idea of the activating supports currently neglects the use of metal fluorides as a basis for catalyst-containing particles. At the same time, positive results of the use of fluorine-modified metal oxides have demonstrated the advantages of fluorine-containing supports (see below); therefore, the use of metal fluorides as catalyst’s supports seems undervalued.

## 3. Silica and Metal Oxides

### 3.1. Silica SiO_2_

Activation of **Zr1** [73,75], as well as **Zr2** and **Zr3** [74], by Me_3_Al using SiO_2_ support in propylene polymerization failed. According to the study of the reaction of (η^5^-C_5_Me_5_)TiMe_3_ (**Ti14′**) with SiO_2_ and Me_3_Al, the formation of Ti–Al and Ti–Al_2_ heterobimetallic species was proposed [144]; however, ethylene uptake by this catalytic system was only mentioned without any experimental data for comparison.

Chemisorption of Me_3_Al on dehydrated silica material MCM-41 (Mobil Composition of Matter No. 41 [145]) of hierarchical microstructure was studied by Anwander et al. [146]. In the reaction product, a SiMe/AlMe population ratio of 0.45, as well as the formation of Lewis acidic centers, were detected in model reactions using Y[N(SiHMe_2_)_2_]_3_(THF)_2_; Group 4 metal complexes on this support were not explored. In 2001, a similar study was conducted by Sano and colleagues [147]. After calcination at 500 °C and thermal pre-treatment at 280 °C (the content of Si–OH groups was ~3 mmol∙g^−1^), MCM-41 was treated with Me_3_Al (4 mmol per 1 g of the support) and, after separation, calcined at 500–800 °C in vacuo; AlMCM-41 supports were obtained in this way. Propylene polymerization was conducted at 40 °C using **Zr1**, and ^i^Bu_3_Al was used as an additional activator/scavenger. The use of MCM-41 in place of the AlMCM-41 provided no polymer. In the case of AlMCM-41, catalytic activity passed through the maximum for support and calcined at 700 °C in vacuo.

Detailed investigation of the solid–liquid reaction of silica with Me_3_Al by ^13^C, ^27^Al, and ^29^Si MAS NMR [148] showed that AlMe_n_ moieties constitute about 70% of the total amount of surface-attached Me groups. The amounts of Si–OMe, SiMe_3_, and SiMe_2_ moieties were approximately equal to each other and totally amounted to ~30% of Me groups. Note that the formation of Si–OMe fragments implies the presence of Si–Al bonds in the reaction product (not detected by NMR); however, the formation of Si–Al bonds was only mentioned in this work, without direct experimental proof. 

The reaction of mesoporous silica SBA-15 with ^i^Bu_3_Al(Et_2_O) resulted in the formation of well-defined (Si_S_O)_2_Al^i^Bu(Et_2_O) and (Si_S_O)_3_Si^i^Bu species via Al→Si transfer of ^i^Bu group [149]. More in-depth and comprehensive studies of the reaction of SBA-15 with Et_3_Al [150], ^i^Bu_3_Al [151,152], and Et_2_AlCl [153] resulted in the detection of a variety of Al_1_ and Al_2_ species, which were chemically bonded with silica surface via Si_S_OAl fragments. Among the products formed after treatment with ^i^Bu_3_Al, (Si_S_O)_2_AlH and (Si_S_O)_3_SiH species were detected [152]. The formation of Si–Al bonds was not discussed in these later works, which would make the very idea of Si–Al bond formation during the reaction of silica with R_3_Al questionable.

In conclusion, we note that the interaction of silica with organoaluminum compounds was used as a first stage of the synthesis of functionalized (e.g., fluorinated) activating supports (see Section 5).

### 3.2. γ-Al_2_O_3_

**Figure 6:**
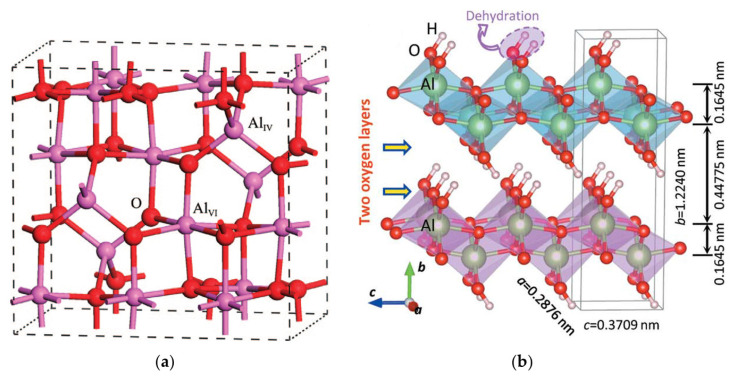
(**a**) Optimized structure of γ-alumina. Reprinted with permission from [155]. Copyright (2008) American Chemical Society. (**b**) Crystal structure of boehmite. Reprinted with permission from [157]. Copyright (2021) International Union of Crystallography.

The γ-Al_2_O_3_ represents the metastable spinel-type cubic phase of alumina obtained by the thermal dehydration (calcination) of aluminum hydroxides and oxyhydroxides [154]. Its bulk structure (Figure 6a) contains an fcc sublattice of oxide ions that generates octahedral and tetrahedral interstices that accommodate Al ions [155,156]. The precursor of γ-Al_2_O_3_ is a well-organized and stable boehmite (AlOOH) that contains a sublattice of cubic close-packaged O^2−^ anions with interstitially situated Al^3+^ cations [157] (Figure 6b). 

**Figure 7:**
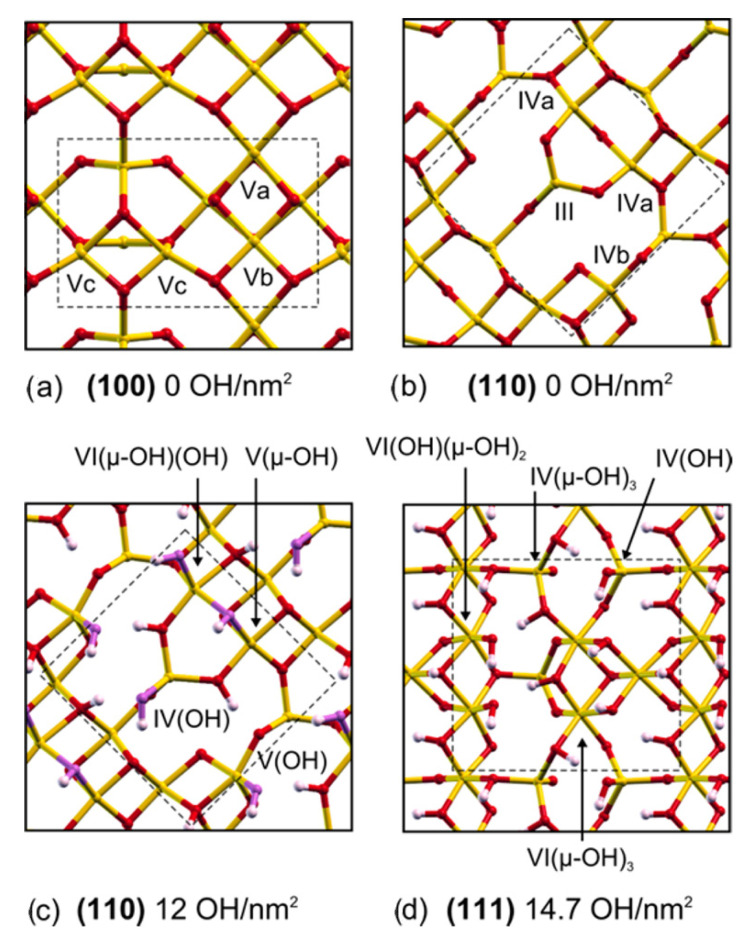
Top views of (**a**) fully dehydrated (100), (**b**) fully dehydrated (110), (**c**) hydrated (110), and (**d**) hydrated (111) terminations of γ-Al_2_O_3_, showing different types of surface Al atoms. X(μ-OH)_w_(OH)_y_ represents an Al atom with a total coordination number of X, including w bridging μ-OH and y terminal OH groups. Only atoms in the top surface layers are shown for clarity. Reprinted with permission from [161]. Copyright (2014) American Chemical Society.

Dehydroxylated and partially dehydroxylated γ-alumina surfaces demonstrate an explicit Lewis acidic character due to the presence of coordinatively unsaturated surface Al sites [158,159,160,161]. From a coordination chemistry point of view, the accessibility of both Brønsted acidic sites (BASs) >Al–OH and Lewis acidic sites (LAS) >Al– on γ-Al_2_O_3_ surface offers a unique complexation environment (Figure 7). The nature of these sites changes with the level of hydroxylation, which is defined and achieved by pre-treatment temperature and duration [159,160,161]. 

During the complex experimental and theoretical study of the surface reactivity of γ-Al_2_O_3_ [160], Wischert et al. showed high efficiency of Al LAS in N_2_ absorption and reactivity of Al,O Lewis acid−base pairs in heterolytic dissociation of H_2_ and CH_4_ molecules with a formation of Al–H and Al–Me species. The maximum site density was observed for alumina, thermally treated at 700 °C, thus confirming the significance of the process on (110) termination of γ-Al_2_O_3_ (see below).

**Figure 8:**
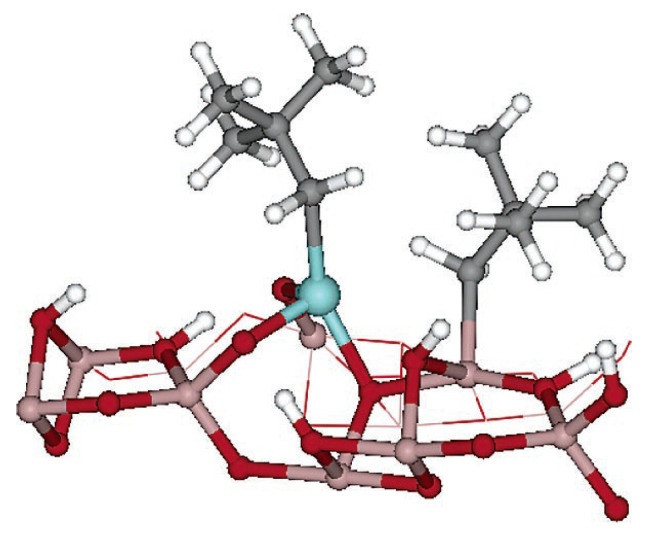
{[(Al_S_O)_2_Zr(CH_2_*^t^*Bu)^+^][Al_S_(CH_2_*^t^*Bu)^–^]} structure optimized on the model surface. Reprinted with permission from [162]. Copyright (2006) American Chemical Society.

The marked difference in reactivity of γ-Al_2_O_3_, calcined at different temperatures, was demonstrated in a number of works, based on the use of the complexes MR_4_. Partially dehydroxylated at 500 °C, γ-Al_2_O_3_ was grafted with Zr(CH_2_*^t^*Bu)_4_, and reaction products contained 2 CH_2_*^t^*Bu per Zr atom [162]. Combined experimental and theoretical (Figure 8) studies allowed the authors to propose the reaction mechanism that includes the formation of (Al_S_O)_2_ZrR_2_ species that further react with the alumina surface through the transfer of one of its R ligands onto an adjacent Al_S_ Lewis center, providing a cationic surface complex [(Al_S_O)_2_ZrR]^+^[(Al_S_)R]^–^. This alkyl transfer was confirmed by a combination of ^13^C CP MAS NMR and chemical shift calculations. In further theoretical and experimental works [158,163], the formation and chemistry of hydride species [(Al_S_O)_2_Zr(H)(μ-H)-Al_VI_] and [(Al_S_O)_2_Zr(H)(μ-R)Al_VI_] along with cationic [(Al_S_O)_2_Zr(H)]+ species stabilized by tetrahedral aluminum hydrides [(Al_IV_-H)^–^] by hydrogenolysis of [(Al_S_O)_2_ZrR]^+^[(Al_S_)R]^–^ was proposed. During the formation of these hydrides or the hydrogenolysis of alkanes, the alkanes transformed into lower homologues to provide methane and ethane as final products. These processes go beyond the subject of our review but seem relevant to the current problem of chemical recycling and upcycling of polyolefins [164]. 

**Scheme 8:**
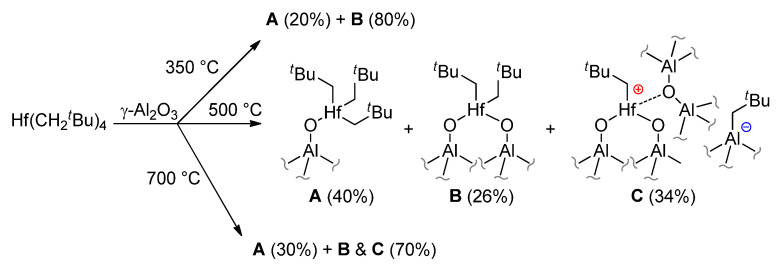
Grafting reaction of Hf(CH_2_*^t^*Bu)_4_ on γ-Al_2_O_3_ at room temperature [159].

Grafting of Hf(CH_2_*^t^*Bu)_4_ on γ-Al_2_O_3_ resulted in different products depending on pre-treatment temperature (Scheme 8): the temperature of 350 °C was not high enough to provide the ability of γ-Al_2_O_3_ to react with Hf alkyl as an activating support [159]. These results were in line with previous experimental and theoretical studies of the Hf(CH_2_*^t^*Bu)_4_/γ-Al_2_O_3_ system [165]. 

Another important aspect of the chemistry of γ-Al_2_O_3_ as an activating support also involved the own reactivity of γ-Al_2_O_3_ towards R_3_Al. As shown in the work of Mazoyer et al. [166], the reaction of calcined γ-Al_2_O_3_ with ^i^Bu_3_Al results in the formation of the surface Al–^i^Bu species, and in the presence of molecular hydrogen, Al–H species are formed. It was found that Al–H species can initiate the polymerization of ethylene (with low activities, ~0.55 kg∙mol^−1^∙h^−1^). However, it can be assumed that surface Al–R and Al–H species can participate in the formation of Group 4 metal catalytic centers on the surface of γ-Al_2_O_3_ when using organoaluminum activators. 

The first study of the activation of Group 4 metallocenes by γ-Al_2_O_3_ was conducted by Marks and colleagues in 1988 [71]. In their experiments, highly dehydroxylated alumina (DA, 150 m^2^∙g^−1^, ~0.1 surface OH∙nm^−2^) or partially dehydroxylated alumina (PDA, 150 m^2^∙g^−1^, ~4 surface OH∙nm^−2^) were mixed with solutions of **Zr2′**, (η^5^-C_5_Me_5_)_2_ZrMe_2_ (**Zr17′**), and (η^5^-C_5_Me_5_)ZrMe_3_ (**Zr25′**) in *n*-pentane, with subsequent elimination of the solvent in He flow and treatment by molecular hydrogen. Hydrogenation of propylene was studied at −63 °C, and turnover frequencies *N*_t_ (s^−1^) amounted to 0.3, 0.2, and 1.1 (DA) and 0.1, 0.06, and 0.3 (PDA) for **Zr2′**, **Zr17′**, and **Zr25′**, respectively. Relative activities in ethylene polymerization were for **Zr2′** >> **Zr17′** ≈ **Zr25′**. The percentage of active sites, determined by their coordination with CO, was found to be 4% for **Zr2′**/DA and 12% for **Zr25′**/DA.

Activation of **Zr1** by Me_3_Al and Et_3_Al using γ-Al_2_O_3_ support in propylene polymerization showed moderate efficiency, which was comparable with the efficiency of MgCl_2_ support [73,75]. A similar pattern was observed for **Zr3**, whereas the catalytic activity of **Zr3** when using γ-Al_2_O_3_ was four times lower than the activity in the presence of MgCl_2_ (42 vs. 172 kg∙mol^−1^∙h^−1^) [74]. **Ti1/** γ-Al_2_O_3_ pre-catalyst, prepared by the reaction of a toluene solution of **Ti1** with γ-Al_2_O_3_ (110 °C, 3 h), demonstrated low activity in propylene polymerization after activation by ^i^Bu_3_Al [126]. Apparently, this was due to the low calcination temperature for γ-Al_2_O_3_ used (200 °C). During comparative studies of the activation of **Zr9** by different metal oxides, treated by ^i^Bu_3_Al, γ-Al_2_O_3_ demonstrated a moderate efficiency relative to other supports [167]. 

**Figure 9:**
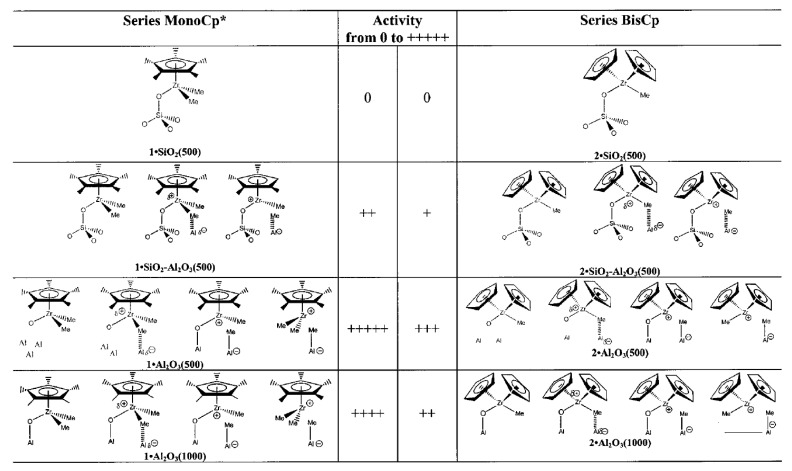
Structure–activity relationship for **Zr25′** and **Zr2′**, supported on γ-Al_2_O_3_. Reprinted with permission from [82]. Copyright (2001) American Chemical Society. The number of “+” symbols reflects catalytic activity according to the assessment of the authors of [82], from very low (+) to very high (+++++).

^13^C CP MAS NMR and EXAFS studies of the reaction of **Zr25′** with γ-Al_2_O_3_ (calcined at 500 °C) showed the presence of Al–O–ZrMe_2_(η^5^-C_5_Me_5_) species; some of the surface complexes underwent further interaction with the γ-Al_2_O_3_ surface, either involving bridging methyl groups between Zr and Al or full Zr→Al transfer of a methyl group [82]. This sample showed moderate activity in ethylene polymerization (~20 kg∙mol^−1^∙bar^−1^∙h^−1^). The results of the ^13^C CP MAS NMR study of the reaction of **Zr2′** with γ-Al_2_O_3_ confirmed the formation of cationic Cp_2_Zr^+^ species, which bonded with alumina via Al–O–Zr covalent or Coulombic interactions [82]. Obviously, only the latter Cp_2_ZrMe^+^ species can be considered catalytically active, but their content was low (activity in ethylene polymerization was only 3 kg∙mol^−1^∙bar^−1^∙h^−1^). In [82], the authors proposed the common scheme of the structure–activity relationship for **Zr25′** and **Zr2′**, supported by γ-Al_2_O_3_ (Figure 9), but the potential catalytic activity of some complexes, presented in this figure, appears doubtful since direct Si–O–Zr or Al–O–Zr bonding will hardly lead to the formation of highly active catalytic species.

**Figure 10:**
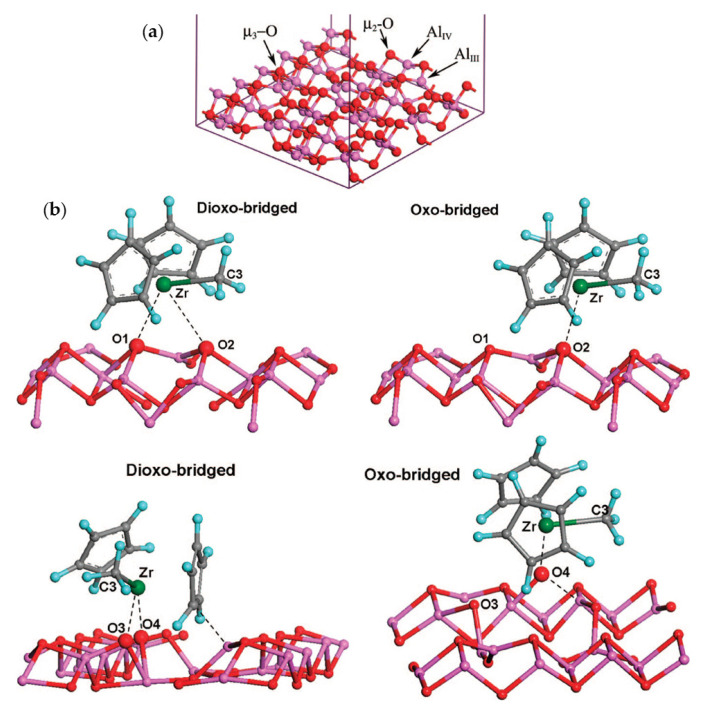
(**a**) Aluminum and oxide sites exposed on the optimized γ-Al_2_O_3_ (110) surface; (**b**) dioxo- and oxo-bridged zirconocenium coordination on μ^3^-O and μ^2^-O surface sites. Reprinted with permission from [155]. Copyright (2008) American Chemical Society.

A more thorough and accurate theoretical study of the structural and catalytic properties of **Zr2′**, chemisorbed on dehydroxylated γ-Al_2_O_3_, was conducted by Marks and colleagues [155]. They developed a γ-Al_2_O_3_ surface model and studied the interactions of **Zr2′** with various surface coordination sites. The results of this modeling were compared and contrasted with data for the homogeneous [Cp_2_ZrMe^+^][MeB(C_6_F_5_)_3_^–^] system. Since theoretical and experimental data for γ-Al_2_O_3_ indicate that the (110) surface predominates (70–80% of the total area), the modeling was focused on the (110) surface (Figure 10a). The optimized alumina (110) surface was found to exhibit significant rearrangement of Al and O ions relative to the bulk; in particular, bulk octahedral Al centers became pseudotetrahedral (Al_IV_ in Figure 10a) while bulk tetrahedral Al centers became pseudotrigonally planar on the surface (Al_III_ in Figure 10a). The surface O ions were found to have either μ^3^-O and μ^2^-O geometries. The μ^3^-O species were bound to Al_III_ and Al_IV_ surface ions and to the octahedral Al_VI_ bulk ion, while the μ^2^-O species were bound to the Al_IV_ and to the octahedral Al_VI_ bulk ions.

The acidic Al sites activate **Zr2′** via Zr–Me scission with a Zr→Al transfer of the Me group to the surface; solid-state NMR data showed that the methide group had been placed on the Al_III_ center at a distance of ~5–8 Å from the metallocenium center. The possible ion pair interactions involving Cp_2_ZrMe^+^ and γ-Al_2_O_3_ surface were scrutinized to search for the most stable configurations for the complexes at μ^3^-O and μ^2^-O sites (Figure 10b). Comparison of the calculated enthalpies of ion pair formation (ΔH_form_) and separation (ΔH_ips_) for chemisorbed species and [Cp_2_ZrMe^+^][MeB(C_6_F_5_)_3_^–^] (Table 2) showed the stronger coordinative capability of the μ^2^-O sites vs. the μ^3^-O sites. Moreover, interactions found in oxo-bridged configurations were significantly stronger than those in the dioxo-bridged configurations. For [Cp_2_ZrMe^+^][MeB(C_6_F_5_)_3_^−^] the adduct formation was less exothermic, but the value of ΔH_ips_ was comparable to that in the heterogeneous μ^3^-O dioxo-bridged complex. 

**Figure 11:**
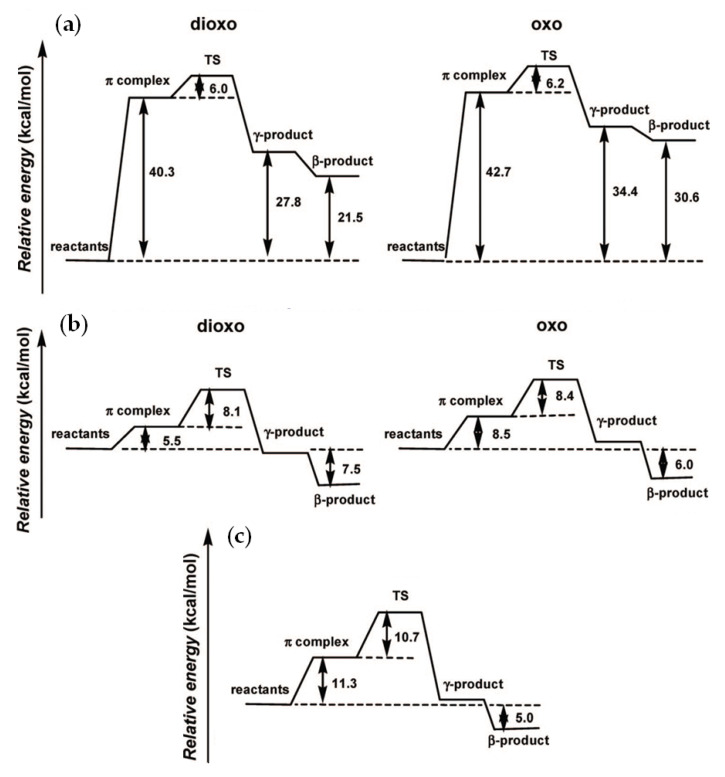
Calculated ethylene enchainment energy profiles for μ^2^-O coordinated (**a**) and μ^3^-O coordinated (**b**) Cp_2_ZrMe^+^ species; the energy profile for [Cp_2_ZrMe^+^][MeB(C_6_F_5_)_3_^−^] is given for comparison (**c**). Reprinted with permission from [155]. Copyright (2008) American Chemical Society.

π-coordination and insertion of ethylene molecule required only partial ion pair separation. These reaction steps were modeled for all four coordination modes of chemisorbed Cp_2_ZrMe^+^ and for [Cp_2_ZrMe^+^][MeB(C_6_F_5_)_3_^−^] (Figure 11). 

As can be seen in Figure 11, π-coordination and insertion of ethylene molecule were easier for μ^3^-O coordinated species even in comparison with [Cp_2_ZrMe^+^][MeB(C_6_F_5_)_3_^−^]. Therefore, only a fraction of the surface sites in dehydroxylated γ-Al_2_O_3_-supported metallocenes are catalytically significant. However, those catalytically significant surface sites exhibit greater catalytic activities than their homogeneous analogs. In addition, it can be assumed that the chemistry of modified alumina supports (see Section 5) may involve surface modification with a decrease in the content of μ^2^-O sites.

Ti(III) complex **Ti15** with the formula [Ti(nacnac)(CH2*^t^*Bu)_2_] (nacnac = [Ar]NC(Me)CHC(Me)N[Ar], Ar = 2,6-(CHMe_2_)_2_C_6_H_3_) [168] was grafted onto alumina that was partially dehydroxylated at 700 °C. This reaction is accompanied by the release of 0.54 equivalents of neopentane per initial surface OH group, which confirms Al–O–Ti bonding with the support. The obtained complex was found to be active in ethylene polymerization, which expands the understanding of the mechanism of α-olefin polymerization by the notion that d^1^ Ti alkyls are possible active sites in the heterogeneous Ziegler–Natta polymerization catalysts.

### 3.3. Silica-Alumina SiO_2_/Al_2_O_3_

**Scheme 9:**
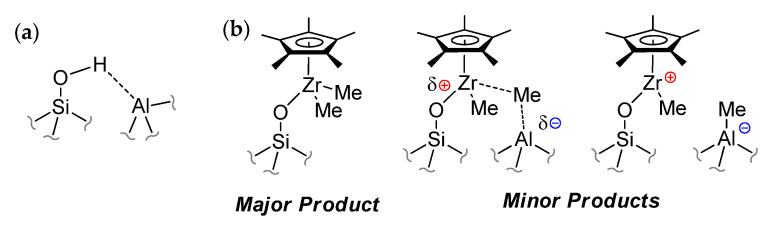
(**a**) Model for Brønsted acidic site (BAS) in silica-alumina [169]; (**b**) the structures of the products of the reaction of **Zr25′** with SiO_2_/Al_2_O_3_ [82].

Due to their combined Lewis and Brønsted acidities, amorphous SiO_2_/Al_2_O_3_ are widespread supports for multifunctional heterogeneous catalysts. The local environment of the acid sites is essential for activation, and in 2010, Chizallet and Raybaud [169] proposed bridging Si–(OH)^…^Al fragments as an active BAS (Scheme 9a). 

During the ^13^C CP MAS NMR and EXAFS studies of the reaction of **Zr25′** with SiO_2_/Al_2_O_3_ [82], different products were detected (Scheme 9b). The main product was the complex with a Si–O–Zr bond, which was observed when studying the reaction of **Zr25′** with silica (see Section 3.1), but the minor products can be considered as trinuclear species with more or less degrees of charge separation. However, experiments on ethylene polymerization showed zero catalytic activity.

In the activation of **Zr9**, ^i^Bu_3_Al-treated SiO_2_–Al_2_O_3_ demonstrated moderate efficiency, which is comparable with the efficiency of Al_2_O_3_ if specific surface areas of the supports are considered [167].

### 3.4. Clay Minerals and Related Systems

**Scheme 10:**
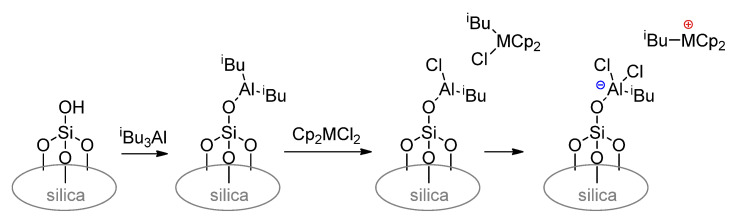
Formation of heterogeneous Ti and Zr metallocene catalysts [170,171].

In 2002, Weiss and colleagues published the results of the study of activation of Group 4 metallocenes by kaolin (vacuum-dried) and montmorillonite (pre-heated at 200 °C) as inorganic carriers with the use of Me_3_Al and ^i^Bu_3_Al as activators [170]. In view of the results of preliminary investigations [171], the authors postulated the mechanism of activation, presented in Scheme 10. Note that this mechanism is quite correlated with the results of the activation of metallocenes by silica/R_3_Al (no activity) [73,74,75].

**Figure 12:**
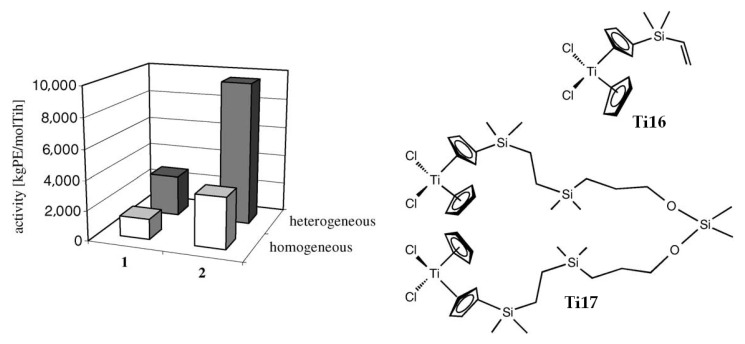
Activities of homogeneous and heterogeneous ethylene polymerization of catalysts **Ti16** and **Ti17**. Reprinted with permission from [170]. Copyright (2002) Elsevier B. V.

Using kaolin/Me_3_Al as an activating support, in the polymerization of ethylene (50 °C, 10 bar), activities of 5.2, 4.3, and 1.5 kg∙mmol^−1^∙h^−1^ were achieved for **Zr2**, Cp_2_ZrHCl (**Zr2″**), and **Ti2**, respectively. On montmorillonite/R_3_Al support **Zr2** demonstrated activities up to 14 kg∙mmol^−1^∙h^−1^, and ^i^Bu_3_Al was found to be a more efficient co-catalyst in comparison with Me_3_Al. In propylene polymerization, **Zr1**/montmorillonite/^i^Bu_3_Al ([Al]/[Zr] = 3200) and **Zr1**/montmorillonite/Me_3_Al ([Al]/[Zr] = 800) catalysts were even more active than the homogeneous **Zr1**/MAO system ([Al]/[Zr] = 1000); the activities amounted to 60.3, 6.8, and 5.7 kg∙mmol^−1^∙h^−1^, respectively. However, the isotacticity of PP, obtained on supported catalysts, was lower (80 and 83% vs. 89%). Another noteworthy result was obtained when studying the activation of homo- and bimetallic Ti complexes **Ti16** and **Ti17**: at [Al]/[Zr] = 2000, the montmorillonite/^i^Bu_3_Al activator was much more efficient than MAO (Figure 12).

Montmorillonite/Et_3_Al system was also studied by Nakano and colleagues in activation of *rac*-Me_2_Si(2-Me-4-Ph-1-Ind)_2_ZrCl_2_ (**Zr26**) [172]. After pre-heating at 200 °C and treatment with Et_3_Al (support 1), 2,6-dimethylpyridine was added in different amounts (supports 2 and 3). Supports 1–3 were reacted with ^i^Bu_3_Al and **Zr26**. UV-vis spectra of the catalysts showed the formation of cationic Zr species only for support 1, containing highly acidic centers.

Since a specific surface area of the support is of great importance for catalytic activity, Sun and Garcés suggest acid lamellar aerogel prepared by exfoliation of clay in acidic media [173]. Supported catalysts prepared by the reaction of aerogel with R_3_Al and **Zr2′** demonstrated activities of 29–69 kg∙mmol^−1^∙h^−1^. Since ^i^Bu_3_Al was the best activator, and Me_3_Al was the worst, the mechanism of activation can not be interpreted through the formation of alumoxanes. The use of spray-dried silica/clay agglomerates for activation of **Zr9** and **Ti8** has been patented by W. R. Grace & Co. [174]. When using 2–6 mmol of ^i^Bu_3_Al or Me_3_Al and 12–120 μmol of pre-catalyst per 1 g of support, activities of 0.3–2 kg_PE_∙g_Cat_^−1^∙h^−1^ were achieved in ethylene polymerization.

**Scheme 11:**
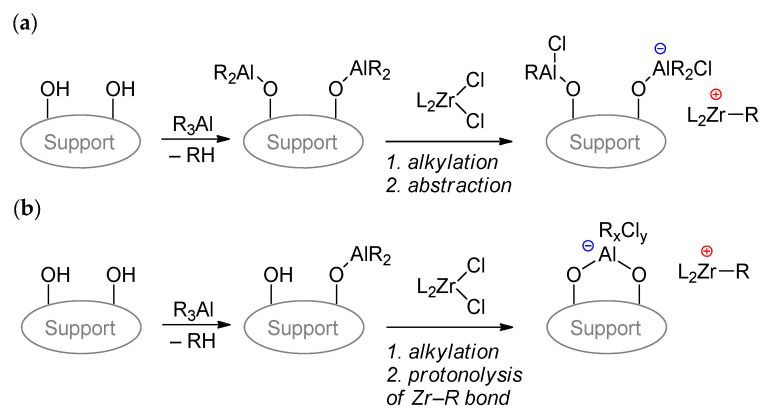
Different activation pathways of a pre-catalyst complex considering the different nature of the acidic sites in the activating support: (**a**) *via* double alkylation; (**b**) *via* single alkylation and proton abstraction [175].

Acid-treated commercial montmorillonite clays (K10, K30) and alumina-modified SBA-15 were studied by Cuenca and colleagues in 2010 [175]. After treatment with Me_3_Al or Et_3_Al, these supports (2.5 g) were mixed with (η^5^-C_5_Me_5_)(η^5^-C_5_H_4_SiMe_2_CH_2_CH=CH_2_)ZrCl_2_ (**Zr27**, 0.007 μmol) and ^i^Bu_3_Al (1 mL) in toluene and studied in polymerization of ethylene (1 bar). Alumina-modified SBA-15 was inactive, whereas all montmorillonite/R_3_Al samples were proven to be activating supports with the best performance of 19.1 kg_PE_∙mmol_Zr_^−1^∙h^−1^ for K10/Me_3_Al. The authors proposed that montmorillonite/R_3_Al supports react with dichloro complex **Zr27** in two pathways (Scheme 11). The first one was the ligand exchange and subsequent alkyl/halide abstraction; the support reacts as an organo-Lewis-acidic cocatalyst similar to MAO (Scheme 11a). An alternative activation process involves protonolysis of the M–R bond on Brønsted acidic centers (Scheme 11b). The Lewis and Brønsted acidities of the supports were determined using pyridine as a probe molecule by monitoring the bands for pyridine absorbed on BAS and LAS at 1540 and 1450 cm^−1^, respectively. It was found LAS comprised the main fraction of the acid sites, detected in montmorillonite and montmorillonite/R_3_Al. Treatment with R_3_Al resulted in a decrease in LAS content, and it was notable that BASs were still detected, though in small numbers, after R_3_Al treatment. 

As a final note, the use of clay minerals did not receive further development due to the creation of more efficient activating supports (See Section 4 and Section 5).

### 3.5. Other Metal Oxides

**Figure 13:**
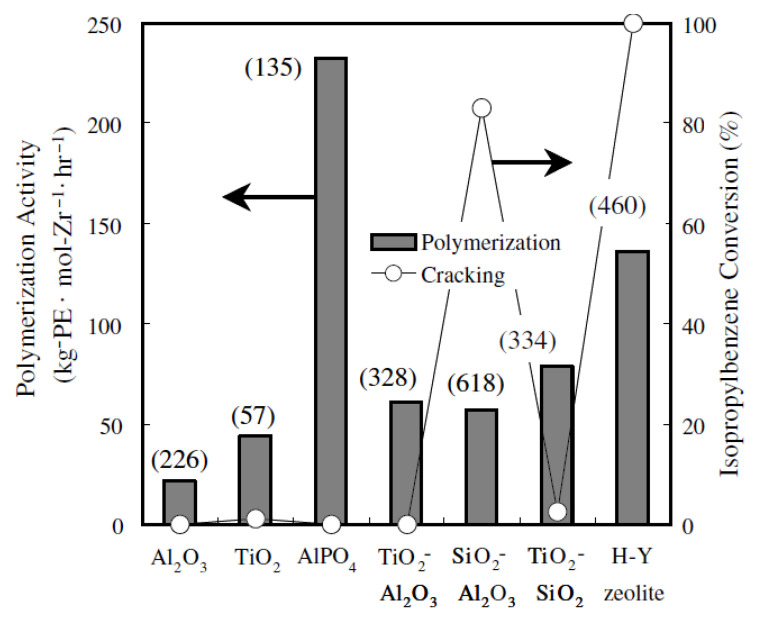
Ethylene polymerization activity using acidic oxide-supported (*n*-BuCp)_2_ZrCl_2_ (**Zr9**), with acid properties as determined by cumene cracking: (1) polymerization (bar): **Zr9** = 4.6 μmol, acidic oxide = 20 mg, TIBA = 0.52 mmol (Al/Zr = 115), temperature = 60 °C, and ethylene pressure = 0.70 MPa; (2) cracking (bent line): reaction temperature = 400 °C, catalyst = 0.10 g, carrier (He) = 30 mL∙min^−1^, and pulse size = 0.5 μL; and (3) BET surface area of each oxide presented in parenthesis. Reprinted with permission from [167]. Copyright (2007) Elsevier B. V.

A comparative study of the activation of **Zr9** by different metal oxides (Al_2_O_3_, TiO_2_, AlPO_4_, TiO_2_–Al_2_O_3_, SiO_2_–Al_2_O_3_, TiO_2_–SiO_2_, and H–Y zeolite), calcined at 500 °C and treated with ^i^Bu_3_Al, in the polymerization of ethylene was conducted by Ikenaga et al. [167]. These oxides were also tested in the cracking of cumene to evaluate their acidity. Among the catalysts under study, the AlPO_4_-supported catalyst showed the highest activity (230 kg∙mol^−1^∙h^−1^). The activity of the zeolite-supported catalyst was two times lower. Catalysts supported on other oxides demonstrated even lower activities. No correlation between the activity and the surface area of the oxides was observed. In cumene cracking (in view of the correlation between the cracking rate and Brønsted acidity), the highest average conversion was obtained using H–Y zeolite. Although SiO_2_–Al_2_O_3_ showed the second-highest cracking activity, this oxide was ineffective as an activator for metallocene. Moreover, the most effective activator of polymerization, AlPO_4_, showed negligible activity in the cracking of cumene (Figure 13).

FT-IR spectral studies of pyridine absorption on Al_2_O_3_ and AlPO_4_ showed more Lewis acidity of the former phase, and the absorption band of pyridinium ions, derived from BAS, was not observed in both cases. Under optimized conditions, the activity of **Zr9** reached 820 kg∙mol^−1^∙h^−1^ (*M*_n_ = 10.0 kDa, *Ð*_M_ = 2.5). At the same time, activities of **Zr2** and **Zr1** were 18 and 66 kg∙mol^−1^∙h^−1^, respectively, and in the latter case PE with *Ð*_M_ = 5.3 was obtained. The formation of active sites on the AlPO_4_ was attributed to the reaction of surface –OH groups with ^i^Bu_3_Al [167]. Nb_2_O_5_ was found to be ineffective in the activation of **Zr25′**; only Nb–O–ZrMe_2_(η^5^-C_5_Me_5_) species have been detected [82].

Modification of silica by MgO, LiOH, or NaOH resulted in the formation of new phases capable of activation of *rac*-[SiMe_2_(η^5^-4,5,6,7-tetrahydroinden-1-yl)]ZrCl_2_ (**Zr28**)/^i^Bu_3_Al in the polymerization of ethylene and propylene [176]. The highest catalytic activities were detected when using SiO_2_/MgO support, they comprised 70 kg_PE_∙g_Zr_^−1^∙h^−1^ and 340 kg_PP_∙g_Zr_^−1^∙h^−1^. The acidity of the surfaces was estimated using a set of indicators, and acid strength in the range of 5.6 < pKa < 3.0 was detected for SiO_2_/MgO. The acid strengths of SiO_2_ and MgO and Mg(OH)_2_ were found to be weak [177]; therefore, in SiO_2_/MgO phase, SiO_2_ and MgO have undergone a reorganization of the bonding structure with a formation of new acidic sites that are required for high activity of zirconocene in propylene polymerization. The role of the basic anionic sites had been also raised in [176], without mechanistic interpretation.

## 4. Sulfated Metal Oxides and Related Systems

More than 40 years ago, it was shown that the acidic sulfate treatment of metal oxides such as ZrO_2_, TiO_2_, SnO_2_, and Fe_2_O_3_ increases the surface acidity and catalytic activity in the processes involving carbenium ions [178]. The Hammett acidity function (*H*_0_) for superacid sites of sulfated metal oxides (SMO) was up to <−16. The common methods of the preparation of SMO are the treatment of precipitated hydroxides with H_2_SO_4_ with subsequent calcination, thermal decomposition of metal sulfate hydrates, or treatment of crystalline oxides with H_2_SO_4_. The features of the methods, as well as specific reaction and thermal treatment conditions, depend on the type of metal oxide and result in supports with different degrees of sulfation, –OH group content, and porosity.

Most of the studies of Group 4 metal complexes, supported on sulfated metal oxides, have been conducted by Marks and colleagues, and their significant proportion relates specifically to the catalytic hydrogenation of arenes [179,180,181]. Along with that, attention has been paid to the nature of the catalytic species and coordination polymerization of α-olefins, which matters for our review as well. 

### 4.1. Sulfated ZrO_2_

The preparation of active sulfated ZrO_2_ (ZrS) is based on the calcination of sulfate-doped zirconium hydroxide or zirconium sulfate hydrates [182,183]. Active SZ is based on tetragonal zirconia (t-ZrO_2_) as the main phase. ZrS, which is based on the more stable monoclinic ZrO_2_, is much less active than tetragonal ZrS [184]. High-resolution transmission electron microscopy (HRTEM) studies of ZrS showed that the presence of SO_4_^2−^ groups stabilizes small tetragonal ZrO_2_ crystallites and induces the formation of well-faceted particles with the (110) plane as the most abundant crystallographic plane [185]. Periodical DFT calculations and FT-IR spectral studies showed a broad variety of structures for sulfated surfaces on t-ZrO_2_ [184].

**Scheme 12:**
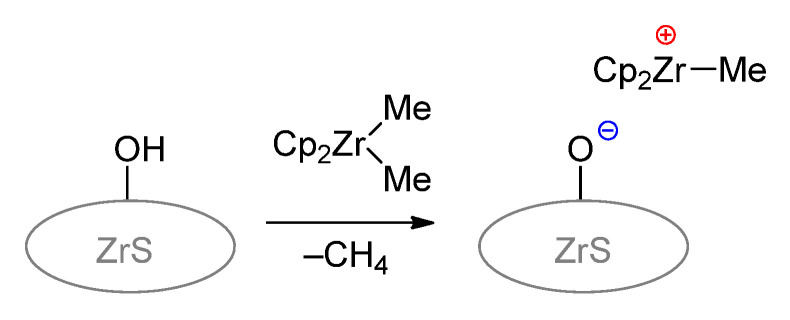
Activation of Cp_2_ZrMe_2_ by sulfated zirconia (ZrS) [83].

In 1998, Ahn and Marks reported the preparation of ZrS by thermal decomposition of Zr(SO_4_)_2_∙4H_2_O with subsequent thermal activation under high vacuum (5 × 10^−6^ Torr) at 300, 400, and 740 °C [83]. ZrS^300^ contained strong BAS/LAS and weak BAS, ZrS^400^ contained both strong BAS and LAS, and ZrS^740^ contained mostly LAS. Next, **Zr2′** and **Zr25′** were adsorbed by acid supports from *n*-pentane solutions. The complex **Zr2′** demonstrated high activity in hex-1-ene hydrogenation, and catalytic activity correlated with strong BAS populations. Less sterically hindered **Zr25′** showed a dramatic enhancement in hydrogenation activity when supported on ZRS^400:^, for example, it mediated fast hydrogenation of benzene at 25 °C and 1 bar H_2_. **Zr2′**/ZrS^400^ and **Zr25′**/ZrS^400^ also catalyzed ethylene homopolymerization at 25 °C with activities of 1.5 and 40 kg∙mol^−1^∙bar^−1^∙h^−1^, respectively. ^13^C CP MAS NMR spectra of the Cp_2_Zr(^13^CH_3_)_2_/ZrS^400^ sample showed the presence of the signals of cationic Zr–Me^+^ species, without the signals of the transferred methide group. The authors have proposed that the sulfated zirconia activates Group 4 metallocenes via Zr–C protonolysis with a formation of ion pairs (Scheme 12). This reaction mechanism is qualitatively different from the Zr→Al migratory mechanism that is characteristic for the γ-Al_2_O_3_ activating support. 

More thorough investigation of the efficiency of ZrS^400^ supports in activation of different Group 4 metal complexes [84] showed that catalytic activities are reduced in a raw Zr(CH_2_Ph)_4_ > Zr(CH_2_SiMe_3_)_4_ > Zr(CH_2_*^t^*Bu)_4_ > **Zr25′** for both ethylene and liquid propylene polymerizations. ^13^C CP MAS NMR spectral studies of **Zr2′**/ZrS^400^ and **Zr25′**/ZrS^400^ showed the presence of both μ-oxo and cationic species, the signals of transferred methide groups were not reliably detected. These results confirm weaker Lewis acidity of sulfated zirconia than that of dehydroxylated γ-Al_2_O_3_.

**Figure 14:**
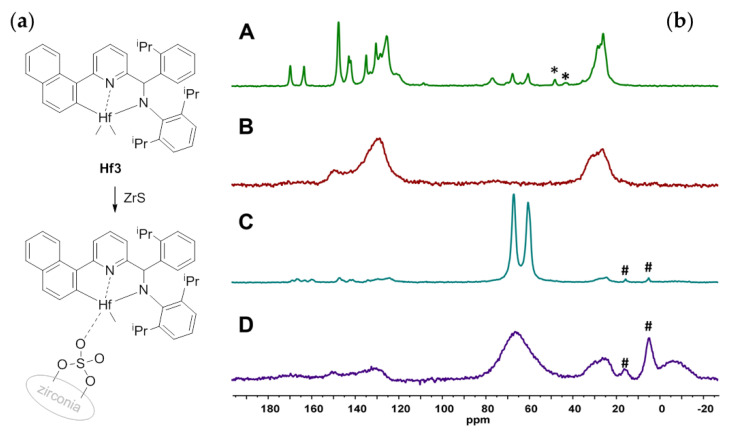
(**a**) Immobilization of **Hf3** (LHfMe_2_) on ZrS support; (**b**) ^13^C CPMAS NMR spectra (100 MHz; repetition time, 5 s; contact time, 2 ms; 10 kHz spinning speed) of (A) LHfMe_2_, 876 scans; (B) LHfMe^+^/ZrS^−^, 10,000 scans; (C) ^13^C labeled LHf(^13^CH_3_)_2_ (60 scans); and (D) LHf^13^CH_3_^+^/ZrS^−^, 1600 scans. *—Rotational sidebands, #—impurities. Reprinted with permission from [186]. Copyright (2018) American Chemical Society.

The further studies of Group 4 polymerization catalysts by Marks’ group [186] were focused on well-known post-metallocene Hf complex **Hf3** (Figure 14a), exhibiting excellent catalytic activity in ethylene homopolymerization and ethylene/α-olefin copolymerization [187,188,189,190,191,192]. In the ^13^C CP MAS NMR spectrum of **Hf3**/ZrS the downfield-shifted broad signal at δ = 65.5 ppm (Figure 14b, D) can be assigned to a “cation-like” electron-deficient LHf^13^CH_3_^+^ species, as evidenced by the marked downfield shift from δ = 60.2 ppm for **Hf3** pre-catalyst (Figure 14b, A). A similar downfield-shifted signal was observed at δ 65.8 ppm in the homogeneous [LHf^13^CH_3_]^+^[MeB(C_6_F_5_)_3_]^–^ [193]. DFT modeling of chemisorption of **Hf3** on the ZrS surface yielded an average distance *d*(Hf–O) of 2.14 Å that is slightly elongated in comparison with typical Hf–OR bonds (1.92–2.03 Å).

Such close contact could not fail to affect the catalytic activity. In ethylene homopolymerization, activities of **Hf3**/ZrS and homogeneous **Hf3**/[Ph_3_C]^+^[B(C_6_F_5_)_4_]^−^ were 4.5 and 6260 kg∙mol^−1^∙bar^−1^∙h^−1^, and activities in ethylene/oct-1-ene copolymerization amounted to 3.9 and 25,600 kg∙mol^−1^∙bar^−1^∙h^−1^. And, it is highly significant that oct-1-ene incorporation in the transition from homogeneous to supported catalyst has fallen from 50 to 2.6 mol% [186]. Another important difference between homogeneous and supported catalysts consisted in opposite directions of first ethylene insertion, at Hf–Aryl and Hf–Me bonds, respectively.

**Scheme 13:**
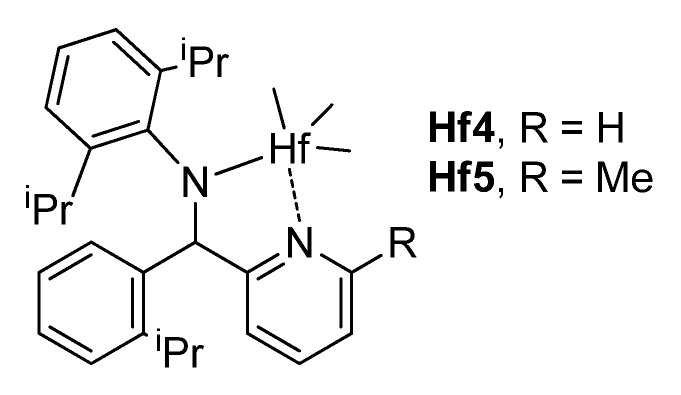
Pyridylamido complexes of Hf, studied in activation and polymerization on sulfated zirconia [194].

In the search for more efficient catalytic systems, pyridylamido complexes **Hf4** and **Hf5** (Scheme 13) were synthesized and studied [194]. Being supported on ZrS, **Hf4** and **Hf5** have demonstrated higher activities in comparison with **Hf3**: 15.7 and 22.7 vs. 4.5 kg∙mol^−1^∙bar^−1^∙h^−1^ in ethylene homopolymerization; 11.5 and 16.6 vs. 3.9 kg∙mol^−1^∙bar^−1^∙h^−1^ on ethylene/oct-1-ene copolymerization. 

### 4.2. Sulfated Al_2_O_3_

Sulfated alumina (AlS), prepared by the impregnation of γ-Al_2_O_3_ with 1.6 M H_2_SO_4_ and subsequent 3 h calcination at 550 °C, was studied as an activating support for (C_5_Me_5_)_2_ZrMe_2_ (**Zr17′**) by Marks and colleagues in 2003 [85]. ^13^C CP MAS NMR spectroscopy revealed that chemisorption proceeds via Zr–Me bond protonolysis with a formation of cationic (C_5_Me_5_)_2_ZrMe^+^ species. In ethylene polymerization experiments (toluene, 60 °C, 10 bar), catalytic activities of Zr complexes were 2100 (Zr(CH_2_Ph)_4_), 1200 (Zr(CH_2_SiMe_3_)_4_), 1100 (**Zr25′**), 160 (**Zr17′**), and 80 (**Zr2′**) kg∙mol^−1^∙h^−1^ [85]. Under similar conditions, activities of these complexes, supported on γ-Al_2_O_3_, were an order of magnitude lower. 

In 2010, Marks’ research group revisited the study of the activation of half-sandwich and sandwich Zr(IV) complexes by AlS. In particular, they prepared nanosized sulfated alumina (n-AlS) by calcination of commercial 25 nm γ-Al_2_O_3_ to remove residual surfactant, surface modification with H_2_SO_4_, and final calcination; TEM analysis indicated that the nanoparticles were loosely aggregated nanorods (diameter∼10 nm, lengths∼10–30 nm) [195]. When studying the chemisorption of **Zr17′** on n-AlS by ^13^C CP MAS NMR spectroscopy, both (C_5_Me_5_)_2_ZrMe^+^ and Al–Me species were detected in contrast with AlS. Experiments on ethylene (co)polymerization on Zr(CH_2_Ph)_4_/n-AlS and **Zr25′**/n-AlS showed higher catalytic activities in *n*-heptane relative to activities in toluene, which is generally not surprising.

**Figure 15:**
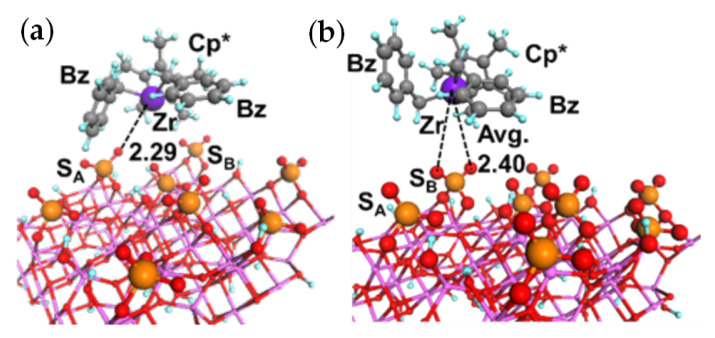
Energy-minimized computed chemisorbed Cp*Zr(CH_2_Ph)_2_^+^ catalyst structures on sulfated alumina surface between site S_A_ (**a**) and S_B_ (**b**) (Cp*—η^5^-C_5_Me_5_). Reprinted and corrected with permission from [180]. Copyright (2015) American Chemical Society.

Increased understanding of chemisorption of Zr(IV) complexes **Zr2′** and **Zr25′** on AlS was the result of further research with the use of Zr X-ray absorption spectroscopy (XAS) and DFT modeling [179]. DFT modeling reveals two types of sulfate species, sites S_A_ (formed by double-exchange/condensation with the surface and does not afford an acidic proton) and S_B_ (formed by single exchange/condensation reaction with the surface, preserving one acidic proton, which is then transferred to an Al-O surface site) as well as (Al)_n_OH hydroxyl groups, with the OH coordination to three or two Al ions. Zr–Me protonolysis is favored at the (Al)_3_OH site, with a formation of Cp_2_ZrMe^+^ or (C_5_Me_5_)ZrMe_2_^+^ species coordinated at sulfate groups. DFT modeling at site S_B_ and experimental studies with the use of Zr K-edge extended X-ray absorption fine structure (EXAFS) and X-ray absorption near-edge structure (XANES) methods yielded a *d*(Zr–O) of 2.37 Å, which was significantly longer than in typical covalent Zr–O bonds. Very similar results were obtained when studying (C_5_Me_5_)Zr(CH_2_Ph)_2_^+^ species [180] (Figure 15). 

**Figure 16:**
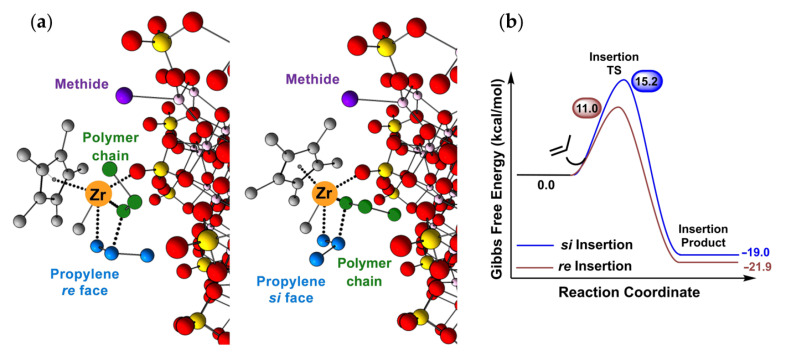
(**a**) *re* and *si* face propylene insertion transition states with **Zr25′**/AlS; (**b**) computed Gibbs free energy profiles for *si* vs. *re* face propylene activation/insertion at the **Zr25′**/AlS catalytic center. Reprinted with permission from [196]. Copyright (2021) Wiley-VCH Verlag GmbH and Co.

A very important and interesting aspect of the use of AlS is associated with the emergence of polymerization stereoselectivity for chemisorbed, initially achiral pre-catalysts such as **Zr25′** [196]. At 25 °C, **Zr25′**/AlS produced polypropylene and poly(hex-1-ene) with 89% and >95% isotacticity, respectively. The requisite **Zr25′**/AlS C_1_ symmetry was attributed to a combination of AlS surface topography and sterically imposing groups beyond the active site. DFT confirmed that propylene coordination and insertion are favored at the *re* enantioface (Figure 16), which results in isotactic polymerization via a ‘back-skip’ mechanism.

**Scheme 14:**
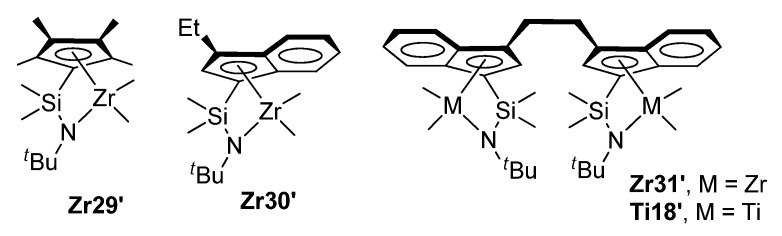
Constrained-geometry Group 4 metal complexes, selected for activation with sulfated alumina [197].

Chemisorption on AlS and catalytic activity of post-metallocene constrained-geometry Group 4 metal complexes **Zr29′**–**Zr31′** and **Ti18** (Scheme 14) in (co)polymerization of ethylene were investigated by Williams and Marks in 2009 [197]. ^13^C CP MAS NMR spectral studies showed that chemisorption of Zr complexes proceeds with a formation of cationic LZrMe^+^ species in two reaction pathways, via Zr–Me bond protonolysis and Zr→Al methide transfer: in contrast with activation of **Zr17′**, surface Al–Me fragments were detected during activation of constrained-geometry complexes.

Experiments on polymerization of ethylene (10 mL of toluene, 5 bar) were conducted with the use of supported catalysts and under homogeneous conditions with the use of perfluoroborate activator [Ph_3_C]^+^[B(C_6_F_5_)_4_]^−^ (Table 3). In all cases, supported catalysts were significantly less active than the homogeneous catalysts, presumably due to steric hindrance from the ancillary ligand-support interaction blocking the ethylene approach to the active site. However, the use of supported catalysts resulted in the formation of high-MW PE, whereas borate-activated **Zr30′** and **Zr31′** produced polyethylene waxes. Ti complex **Ti15** was more active than Zr catalysts. For ethylene/1-hexene copolymerizations at 25 °C, binuclear complex **Ti18**/AlS was found to be the most active (16.4 kg∙mol^−1^∙h^−1^), followed by **Zr31′**/AlS, **Zr30′**/AlS, and **Zr29′**/AlS (1.24, 1.21, and 0.54 kg∙mol^−1^∙h^−1^, respectively). In this way, the cooperative ‘binuclear effect’, observed previously by Marks and colleagues for bimetallic Group 4 constrained-geometry complexes in homogeneous conditions [198,199,200], does not occur when using supported catalysts. 

**Figure 17:**
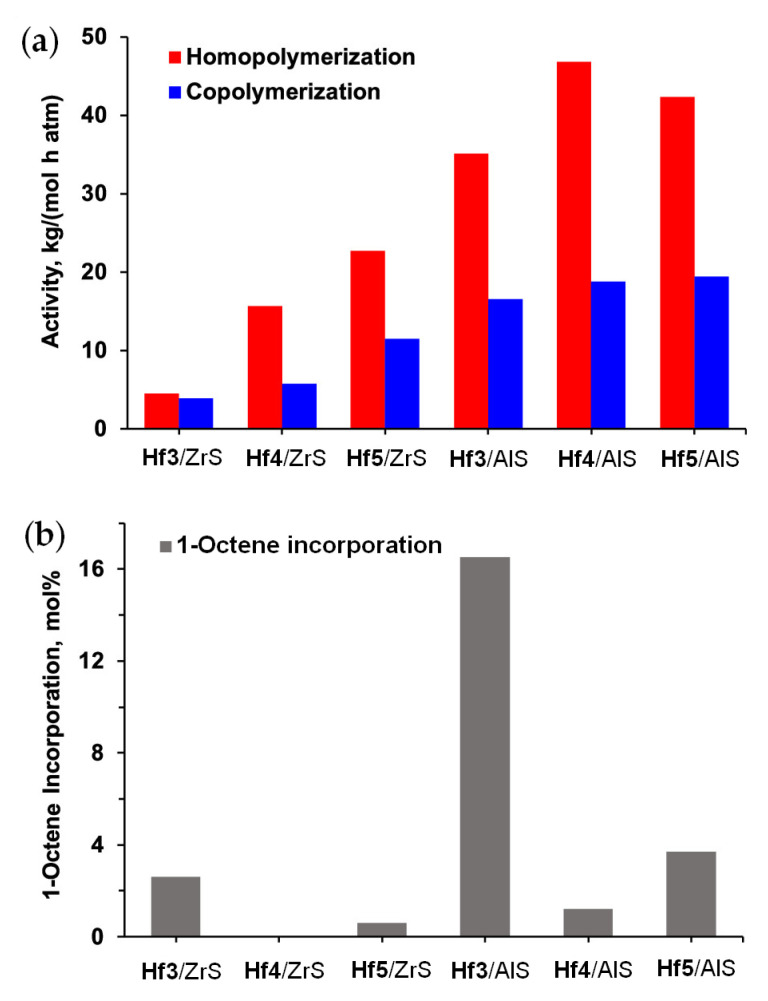
Effects of pre-catalyst structure and support on (**a**) activity and (**b**) oct-1-ene incorporation in ethylene copolymerization using the indicated surface molecular catalysts. Reprinted with permission from [194]. Copyright (2021) American Chemical Society.

During activation of a highly efficient industrialized pyridylamido-naphthyl Hf pre-catalyst **Hf3** (see Figure 17) by AlS, both protonolysis and methide Hf→Al transfer were detected [194]. Hf EXAFS spectrum of **Hf3**/AlS allowed to determine the ligand environment of Hf as two Hf−N (2.30 and 2.08 Å), one Hf−C (2.26 Å), one Hf−Me (2.21 Å), and one Hf−O_support_ (2.06 Å). Comparative studies of supported **Hf3** and pyridylamido complexes **Hf4** and **Hf5** showed that when using AlS these complexes demonstrate close catalytic activities (35.1–46.8 kg∙mol^−1^∙bar^−1^∙h^−1^ in ethylene homopolymerization, 16.6–19.4 kg∙mol^−1^∙bar^−1^∙h^−1^ in ethylene/oct-1-ene copolymerization), which was not observed when using ZrS as an activating support [194]. Oct-1-ene incorporation was also dependent on the pre-catalyst and support used (Figure 17). 

**Scheme 15:**
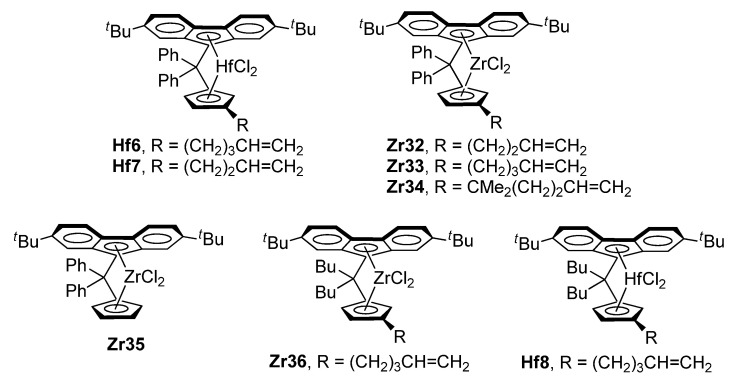
Metallocenes studied in ethylene polymerization using AlS/R_3_Al support [201].

A rather high efficiency of AlS as activators for Group 4 metallocenes could not fail to interest chemists and chemical engineers employed in the polyolefin industry. AlS was the subject of a number of patents, filed by Chevron Phillips. In particular, in ethylene polymerization with the use of zirconocene **Zr9**, AlS, and Et_3_Al activators, catalytic activities up to 3.23 kg∙g_AlS_^−1^∙h^−1^ have been reached [92]. However, it should be borne in mind that catalytic efficiency of metallocene/AlS/R_3_Al systems is critically dependent on many factors such as the characteristics of Al_2_O_3_, method of sulfation, [SO_4_^2−^]/m_alumina_ ratio, conditions of calcination, nature of Group 4 metal complex and R_3_Al, and others. Therefore, for example, the highest activity of 3.23 kg∙g_AlS_^−1^∙h^−1^ was achieved when AlS was prepared by impregnation of (NH_4_)_2_SO_4_ solution onto Ketjen grade B alumina (1.5 mmol of sulfate per 1 g of alumina), followed by vacuum-drying and calcination in air at 550 °C [92]. The role of the pre-catalyst can be illustrated by the data from the patent [201] (Scheme 15, Table 4), the maximum productivity of 17.3 kg∙g_Cat_^−1^∙h^−1^ was achieved when using **Zr36**/^i^Bu_3_Al in homopolymerization of ethylene in the absence of H_2_. In this patent, only calcining conditions are given (heating to 550 °C with a 240 °C∙h^−1^ ramp rate in air and 3 h hold at 550 °C). 

For the synthesis of PE composites, the chemists of Chevron Phillips proposed the use of binary mixtures of metallocene catalysts, the first being the catalyst of the formation of low-MW PE (*M*_w_ 5–50 kDa, for example, (η^5^-Indenyl)_2_ZrCl_2_ (**Zr37**), and the second being the formation of high-MW PE (*M*_w_ 200–1200 kDa, for example, **Zr33** [202]. AlS support was prepared by the reaction of bohemite (‘Alumina A’ from W. R. Grace & Co., Columbia, MD, USA) with aq. (NH_4_)_2_SO_4_, followed by vacuum-drying and calcination at 400–600 °C. The combination of binary pre-catalyst, AlS, and ^i^Bu_3_Al allowed to obtain ethylene/hex-1-ene copolymers with unique mechanical and rheological characteristics that may be employed for the production of large diameter pipes. Polymerization experiments were conducted with the use of molecular hydrogen (350 ppm with respect to the ethylene flow) at 92–95 °C. Maximum catalytic activities amounted to 2.84 and 3.16 kg_PE_∙g_Cat_^−1^∙h^−1^ for ethylene homo- and copolymerizations, respectively. 

The importance of pre-treatment of AlS by R_3_Al was the subject of the patent [203]: when using metallocene dichlorides, such pre-treatment resulted in significant increase of the catalytic activity. From the mechanistic viewpoint, pre-treatment of AlS by organoaluminum compounds results in the substitution of all BAS by –OAlR_2_ fragments, and this modified support retains the ability to absorb LZrCl_2_ via ligand exchange and Zr→Al alkyl transfer to LAS. However, from the analysis of scientific periodicals, this issue remains unexplored. An efficient, but incomprehensible, method to increase the efficiency of metallocene/AlS/R_3_Al systems, proposed in the patent [204], is based on using simultaneous mixing of the catalyst components that results in ~30% improvement of catalytic activity. This effect still remains unexplored with the use of modern analytical methods. 

### 4.3. Other Sulfated Metal Oxides

Sulfated tin (IV) oxide microparticles were prepared by sedimentation of Sn(OH)_4_ gel at pH 8, elimination of residual Cl^–^ by aq. NH_4_OAc, and drying and reaction with 3M H_2_SO_4_, followed by calcination at 500 °C for 3 h [86]. ^13^C CP MAS spectral study of chemisorbed **Zr17′** showed the formation of cationic species, Sn_S_–O–Zr(C_5_Me_5_)_2_Me were detected in trace amounts. In ethylene polymerization experiments (toluene, 60 °C, 10 bar), catalytic activities of Zr complexes were 660 (Zr(CH_2_Ph)_4_), 98 (**Zr25′**), 15 (**Zr17′**) and 8.6 (**Zr2′**) kg∙mol^−1^∙h^−1^. 

In 2004, Marks and colleagues undertook a comparative study of sulfated metal oxides ZrS, AlS, SnS, FeS, and TiS as activators for **Zr2′** [87]. Solid-state ^13^C NMR spectroscopic analysis of the Cp_2_Zr(^13^CH_3_)_2_ adsorbate complexes reveals that each support affords a different ratio of μ-oxo and cationic species. The chemical shift of the signals of Me groups in cationic species (δ = 37.1, 35.7, 37.6, and 33.8 ppm for ZrS, SnS, FeS, and TiS, respectively) correlated with the efficiency of activating supports. In ethylene polymerization experiments (60 °C, 10 bar, using 30 mg of the catalyst), **Zr25′** and Zr(CH_2_Ph)_4_ have demonstrated wide spectra of catalytic activities depending on the type of support (Table 5); sulfated zirconia was the most efficient activating support. 

## 5. Fluorinated Metal Oxides and Related Systems

As was shown in Section 3, some metal oxides have demonstrated the ability to activate Group 4 metallocenes and post-metallocenes, which is primarily due to the high Lewis acidity of the metal centers on the support surface. The obvious way to increase the Lewis acidity consists in the replacement of the oxygen atom in the ligand environment of the acidic metal center by a more electronegative element that is fluorine, without options. On the other hand, partial chlorination can also be effective.

However, in any case, the starting oxide should already be acidic. And this is the reason why fluorinated silica was not used in catalysis [205] and, in particular, has demonstrated zero efficiency as an activating support for zirconocenes [89,90]. On the contrary, in the case of alumina and especially silica/alumina, surface modifications by introducing halogen (F, Cl) resulted in supports with high acidity and bright prospects for use in the polyolefin industry.

### 5.1. Fluorinated Al_2_O_3_

During the studies of gas-phase disproportionation of fluorochloroalkanes with the use of γ-Al_2_O_3_ catalyst, fluorinated alumina Al_2_O_3_(F) was formed [206]. γ-Al_2_O_3_ alone was inactive and required pre-treatment with HF (or fluorocarbon); in this way, it is Al_2_O_3_(F) that is a true catalyst. Although chemical analyses reveal significant amounts of F in the solid after this activation procedure, nearly no structural changes of the γ-Al_2_O_3_ were detected by X-ray diffraction (XRD) [206]. Only after a few days, crystalline α-AlF_3_ becomes detectable. Other approaches to Al_2_O_3_(F) are based on ‘wet’ methods, the reaction of γ-Al_2_O_3_ with NH_4_F or with NH_4_HF_2_ in aqueous media [207], and the content of F in the reaction product is easily determined by γ-Al_2_O_3_/[F^−^] ratio. The reaction of Al_2_O_3_ with HF (Equation (1)) is exergonic (ΔG = −329 kJ∙mol^−1^), and the formation of AlF_3_ during the ‘fluorination’ of alumina should not be ruled out.

Al_2_O_3_ + 6 HF → 2 AlF_3_ + 3 H_2_O (1)

In this way, both prolonged gas-phase fluorination or an excess of fluoride when being used in a ‘wet’ process may result in a high fluorine content with a formation of AlF_3_ after calcination. The problem is that the most stable crystalline phase of AlF_3_, namely α-AlF_3_, does not exhibit the properties of strong Lewis acid since the surfaces derived from α-AlF_3_ are covered completely with basic fluoride anions, irrespective of the plane indexes [208]. It is relevant to remind here that crystalline AlF_3_ was found to be a low-efficient activating support for **Zr1** in the presence of Me_3_Al in the early work of Soga and Kaminaka [75]. It is quite possible that these unsuccessful experiments were conducted with large-sized crystallites of α-AlF_3_, whereas AlF_3_ showed high Lewis acidity only as β-AlF_3_ crystal modification or in the amorphous form [208,209]. Efficient methods of the synthesis of high-surface amorphous AlF_3_ have been developed in the early 2000s [210,211]. These methods consist of a fluorolytic sol–gel reaction starting from aluminum isopropoxide, followed by an activation step under the flow of a fluorinating gas. A similar method was also used for the preparation of Al_2_O_3_(F) with relatively low [F]/[Al] ratios (0.2–0.5) by the reaction of Al(OCH(Me)Et)_3_ with HF in ^I^PrOH with subsequent hydrolysis and calcination [212]. Sol–gel methods are too complex, and fluorination of γ-Al_2_O_3_ seems preferable for the production of highly acidic Al-based supports. 

However, the chemical nature of the surface of Al_2_O_3_(F) is not entirely clear. Back in the 2000s, Fischer et al. performed a solid-state NMR study of fluorinated γ-Al_2_O_3_ that showed the presence of the species with ^VI^Al(O_6−n_F_n_) (n = 1, 2 or 3) environments [213]. With an excess of F^−^, the remaining weakened Al–O–Al bonds anchoring ^VI^Al(O_3_F_3_) to the bulk of alumina were eventually broken with the formation of AlF_3_∙3H_2_O. 

The nature of LAS and BAS formed on the alumina surface during fluorination and the efficiency of fluorinated Al_2_O_3_ supports in the activation of zirconocenes **Zr2** and **Zr2′** were studied by Panchenko et al. [214]. Four samples of Al_2_O_3_(F) with different F content, were prepared by treatment of γ-Al_2_O_3_ (specific surface area 196 m^2^∙g^−1^, pore volume 0.58 cm^3^∙g^−1^) with aqueous solutions of NH_4_F and subsequent calcination in air at 500 °C for 2 h. Then, the supports were treated with a toluene solution of **Zr2** or hexane solution of **Zr2′** using a ratio of 0.05 g Zr per gram of support. Ethylene polymerization was conducted at 80 °C and an ethylene pressure of 10 bar; in the case of **Zr2**/γ-Al_2_O_3_ and **Zr2**/Al_2_O_3_(F), ^i^Bu_3_Al was added.

Determination of LAS and BAS on the surfaces of γ-Al_2_O_3_ and Al_2_O_3_(F) was conducted using additionally dehydroxylated samples (500 °C, 0.02 Torr, 1 h) by FT-IR studies of the adsorption of CO (LAS) and pyridine (BAS). The concentrations of LAS and BAS (*C*_S_) were estimated from the integrated intensity of the CO absorption bands in the region 2175–2240 cm^−1^ and pyridinium ion absorption bands with a maximum of 1535–1550 cm^−1^ using Equation (2).

(2) $$C_{S}=\frac{A}{A_{0}\cdot p\cdot 10^{-3}}$$

where *C*_S_—concentration of acidic sites (μmol∙g^−1^), *A*—integrated absorption of the examined band (cm^−1^), *A*_0_—integral adsorption coefficient for CO or pyridine adsorbate concentration 1 μmol∙g^−1^ (cm∙μmol^−1^), and *p*—amount of the sample per 1 cm beam, g∙cm^−2^. The values of *A*_0_ for CO absorption bands at 2215, 2210, 2202, 2198, and 2180 cm^−1^ are 1.15, 1.10, 1.02, 0.95, and 0.8 cm∙μmol^−1^, respectively; for pyridinium, the ion absorption band *A*_0_ is equal to 3 cm∙μmol^−1^.

The strength of BAs was characterized by the proton affinity (*PA*), which was calculated from the IR spectra of adsorbed pyridine by Equation (3). Characteristics of the supports are given in Table 6.

(3) $$PA=\frac{\mathrm{lg}(3400-\nu^{cg})}{0.0023}-51$$

where 3400 cm^−1^ is the frequency of N–H stretching vibration for free pyridinium ion, and *ν^cg^* is the position of the gravity center of N–H stretching vibration for pyridinium ion on the support surface.

Aluminas, calcined at temperatures above 500 °C, contain four types of LAS. In the IR spectra of adsorbed CO, these LAS are characterized by absorption bands at 2178–2182 (L-1), 2185–2195 (L-2), 2203–2210 (L-3), and 2225–2240 cm^−1^ (L-4). The L-1, L-2, and L-3 LAS correspond most likely to coordinatively unsaturated aluminum atoms in an octahedral environment, while L-4 corresponds to those in a tetrahedral or pentahedral environment. Weak LAS of the L-1 and L-2 types were found on the surface of Al_2_O_3_(F) samples. In addition, the spectra of CO, adsorbed on Al_2_O_3_(F), contain absorption bands at 2215–2208 cm^−1^ corresponding to new, stronger sites (L-3) (Table 6). LAS of maximum strength (absorption band 2215 cm^−1^) were observed in the Al_2_O_3_(5%F) sample. As shown by Corma et al. [215], the addition of HF to Al_2_O_3_ can affect the strength of LAS via direct introduction of one F^−^ (L-2) or two F^−^ (L-3) into the coordination sphere of aluminum. As can be seen in Table 6, the introduction of fluorine into Al_2_O_3_ sharply decreases the total amount of LAS but increases the content of strong type L-3 LAS. In Al_2_O_3_(F) samples with a fluorine content of 3–7 wt%, strong BAS with PA 1190 kJ/mol were detected. The concentration of these sites was 2–7.3 μmol∙g^−1^ with a maximum in the Al_2_O_3_(5%F) sample. 

The content and strength of basic sites at the support surfaces were estimated by analyzing FT-IR spectra of adsorbed CDCl_3_, and it was shown that fluorination of Al_2_O_3_ results in formation of weak basic sites.

The catalysts prepared with the use of dimethyl zirconium complex **Zr2′** were active in the ethylene polymerization without additional cocatalyst ^i^Bu_3_Al, indicating the formation of the active sites containing a Zr–Me bond by the direct interaction of zirconocene with LAS and BAS of the support. In the polymerization run with ^i^Bu_3_Al, the activity was only slightly higher (620 kg_PE_∙mol_Zr_^−1^∙h^−1^) than that without ^i^Bu_3_Al (538 kg_PE_∙mol_Zr_^−1^∙h^−1^). The catalyst **Zr2′**/Al_2_O_3_ showed low activity; the activities of **Zr2′**/Al_2_O_3_(F) were noticeably higher with a maximum observed for **Zr2′**/Al_2_O_3_(5%F). In the presence of ^i^Bu_3_Al, the activity of **Zr2**/Al_2_O_3_(F) catalysts was higher than that of **Zr2′**/Al_2_O_3_(F). This result was attributed to the formation of inactive μ-oxo-like zirconium compounds during the reaction of **Zr2′** with BAS of the support. The activity of **Zr2**/Al_2_O_3_(F) catalysts also depended on fluorine content in the support; **Zr2′**/Al_2_O_3_(5%F) showed a maximum activity (Table 6) [214]. In this way, Al_2_O_3_(F) was a more effective activating support in comparison with γ-Al_2_O_3_.

The above-discussed works are of more theoretical than practical value. Al_2_O_3_(F) was used for the activation of Cr-based catalysts of ethylene polymerization in 1992 [216]. In the early 2000s, the chemists of Phillips Petroleum (subsequently Chevron Phillips) paid special attention to fluorinated alumina and related phases as activating supports for Group 4 metallocenes. In particular, Al_2_O_3_(F) was prepared by the ‘wet’ method from Ketjen B alumina (Akzo Nobel, surface area 340 m^2^∙g^−1^, pore volume 1.78 mL∙g^−1^) with the use of NH_4_HF_2_ as an F^−^ source at different [F]/[Al] ratios [89]. After calcination at ~500 °C in dry air, these supports were tested in combination with **Zr9** and Et_3_Al in ethylene homopolymerization. Activities of 240–950 g_PE_∙g_Cat_^−1^∙h^−1^ were achieved (while the activity was 6.9 g_PE_∙g_Cat_^−1^∙h^−1^ when using γ-Al_2_O_3_ in a comparative experiment), and the relationship between efficiency of the support and fluorine content had a distinct maximum. 

Preparation of Al_2_O_3_(F) from Ketjen B alumina by ‘wet’ method (optimal calcining temperature 600 °C) and from HPV alumina (W. R. Grace & Co., surface area 500 m^2^∙g^−1^, pore volume 2.8 mL∙g^−1^ after pre-calcining at 600 °C) by heating in fluidizing N_2_ and *n*-C_6_F_14_ vapor at 600 °C was described in [92]. In the latter case, the support turned black presumably due to carbon deposition. In ethylene polymerization (90 °C, 38 bar) with **Zr9** and Et_3_Al, the highest activities of 1.25 and 1.27 kg_PE_∙g_Cat_^−1^∙h^−1^ were achieved for supports, which were prepared by ‘wet’ and gas-phase fluorination. After recalcining of ‘black’ support in the air at 600 °C, during which the black color turned back to white, catalytic activity increased to 2.18 kg_PE_∙g_Cat_^−1^∙h^−1^. 

### 5.2. Fluorinated SiO_2_/Al_2_O_3_

Silica/alumina appears not to be an efficient activating support per se (see Section 3.3); however, the chemical binding of fluorine proved to be able to fundamentally increase the ability of supports to provide activation of Group 4 metal complexes. When compared with other activating supports, it is this type of support that showed the best efficiency, which puts fluorinated SiO_2_/Al_2_O_3_ on the top of the activating supports for Group 4 metallocenes and post-metallocenes. Deep and comprehensive corporate studies of this type of support have been undertaken (and apparently are ongoing) for Chevron Phillips (previously Phillips Petroleum), Total, and INEOS, which are the leading polyolefin manufacturers.

**Scheme 16:**
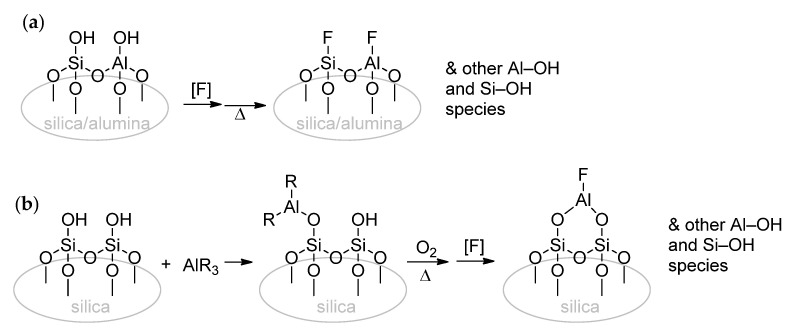
Two distinct approaches to fluorinated SiO_2_/Al_2_O_3_. (**a**) Fluorination of surface –OH groups in SiO_2_/Al_2_O_3_; (**b**) Surface modification of SiO_2_ by aluminum alkyls, followed by oxidation and fluorination. The content of Si–OH and Al–OH surface fragments depends on calcination temperature and duration.

These studies were aimed at finding the most effective solutions in the development of MAO- and borate-free activating supports, and were based on well-defined and commercially available inorganic phases with known porosity and surface area. However, these studies were conducted in fundamentally different directions, namely, fluorination of the commercial silica/alumina phases and surface treatment of commercial silicas by organoaluminums with subsequent thermal treatment and fluorination (optionally, the latter stage was omitted when alkylaluminum fluorides were used). Consequently, these different approaches (Scheme 16) are discussed separately in our review.

Note that some recent patents protect the somewhat unexpected type of activating supports, namely, fluorinated silica-grafted alumina (Al_2_O_3_/g-SiO_2_(F), see Section 5.2.3). 

#### 5.2.1. Fluorination of SiO_2_/Al_2_O_3_ (SiO_2_/Al_2_O_3_(F))

**Figure 18:**
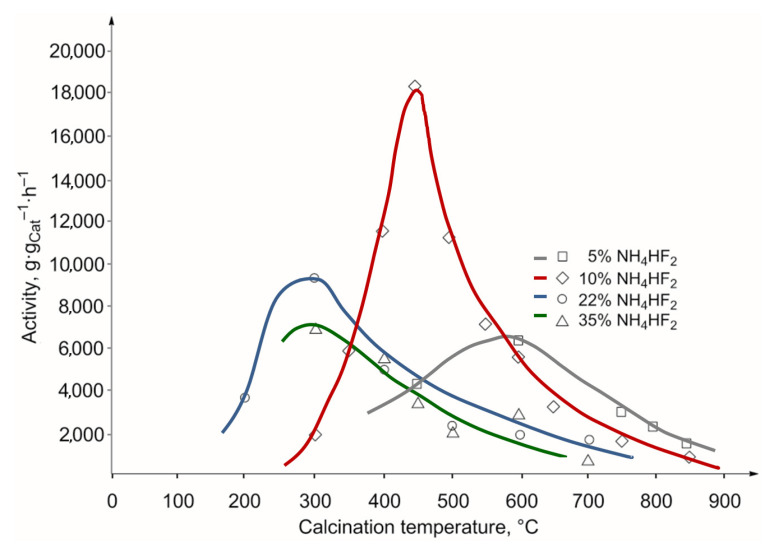
The function of catalyst activity on calcination temperature for **Zr9**/Et_3_Al system and SiO_2_/Al_2_O_3_(F) activating supports, prepared with different NH_4_HF_2_ loadings. Designed based on the data presented in [89].

The common ‘wet’ method of the fluorination of silica/alumina, which was studied since the early 2000s by the chemists of Phillips Petroleum, is completely similar to the ‘wet’ method of the preparation of fluorinated γ-Al_2_O_3_. In early experiments, the samples of SiO_2_/Al_2_O_3_ MS 13-110 (W. R. Grace & Co., 13 wt% alumina, surface area 400 m^2^∙g^−1^, pore volume 1.2 mL∙g^−1^) were treated by aqueous NH_4_HF_2_ at different [F]/[Al] ratios [89]. After calcination at different temperatures, the supports were used for activation of the **Zr9**/Et_3_Al catalyst. The results of polymerization experiments have demonstrated that both [F]/[Al] ratio and calcination temperature affect the polymerization activity, and optimal calcination temperatures vary greatly for supports, which are prepared at different [F]/[Al] ratios (Figure 18). In the absence of fluorinated SiO_2_/Al_2_O_3_ support, the **Zr9**/Et_3_Al system was inactive. 

**Scheme 17:**
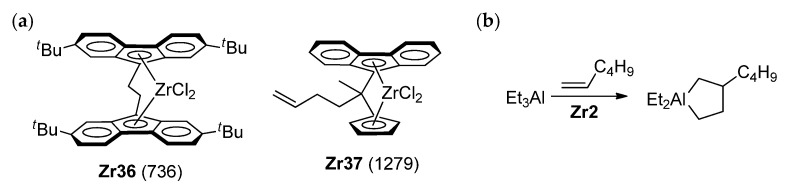
(**a**) Structures of zirconocene pre-catalysts and catalytic activities in ethylene polymerization (in brackets, g_PE_∙g_Cat_^−1^∙h^−1^) of the corresponding heterogeneous supported catalysts, prepared with the use of fluorinated SiO_2_/Al_2_O_3_ and Et_3_Al; (**b**) formation of cyclic organoaluminum species during ‘pre-contacting’ of **Zr2**/Et_3_Al with hex-1-ene [93].

Fluorinated SiO_2_/Al_2_O_3_ (MS 13-110, W. R. Grace & Co.), prepared under optimized conditions, was studied in the polymerization of ethylene using **Zr2**/Et_3_Al with and without pre-contacting of **Zr2**/Et_3_Al with hex-1-ene before interaction with an activating support [93]. The reaction of **Zr2** with Et_3_Al and hex-1-ene (toluene/*n*-heptane, 30 min) prior to charging to the support resulted in a marked increase of activity (2932 vs. 1640 g_PE_∙g_Cat_^−1^∙h^−1^). Under similar conditions, bis(η^5^-fluorenyl) and (η^5^-fluorenyl)-Cp *ansa*-zirconocenes **Zr36** and **Zr37** (Scheme 17a) have demonstrated lower activities. 

An increase in catalytic activity was attributed in [93] to the formation of aluminum metallocyclic species (Scheme 17b). Despite the experimentally proven fact of the formation of similar species during the reaction of **Zr2** with Et_3_Al [28,29], their possible role in further activation and polymerization processes is unclear.

The addition of arylboronic acids to the solutions of **Zr2** and **Zr2′**, followed by mixing with SiO_2_/Al_2_O_3_(F)/^i^Bu_3_Al activator, resulted in a marked increase of catalytic productivity in the case of **Zr2′** [95]. One can assume that **Zr2′** reacts with ArB(OH)_2_ with a formation of Cp_2_Zr–O–B(OH)Ar species, but the role of similar species in further transformations is unclear too.

Very promising results were obtained when Ketjen Grade B alumina was subjected to ‘wet’ (with final calcining at 500 °C) or gas-phase fluorination (at 600 °C), followed by gas-phase chlorination (CCl_4_, 600 °C). The activities of the **Zr9**/Et_3_Al system on these supports in ethylene polymerization reached 3.1 and 6.3 kg_PE_∙g_Cat_^−1^∙h^−1^, respectively [92]. 

#### 5.2.2. Treatment of SiO_2_ by Organoaluminums with Subsequent or Simultaneous Fluorination (SiO_2_/g-Al_2_O_3_(F))

The synthesis of type SiO_2_/g-Al_2_O_3_(F) supports and their use for activation of Group 4 metallocenes and post-metallocenes are described and discussed in a number of patents (owned by Elf Atochem/Total and INEOS) and Total in cooperation with the scientific group of Boisson [99,100,101,102,103,104] and only a few scientific articles [105,106,107]. 

**Scheme 18:**
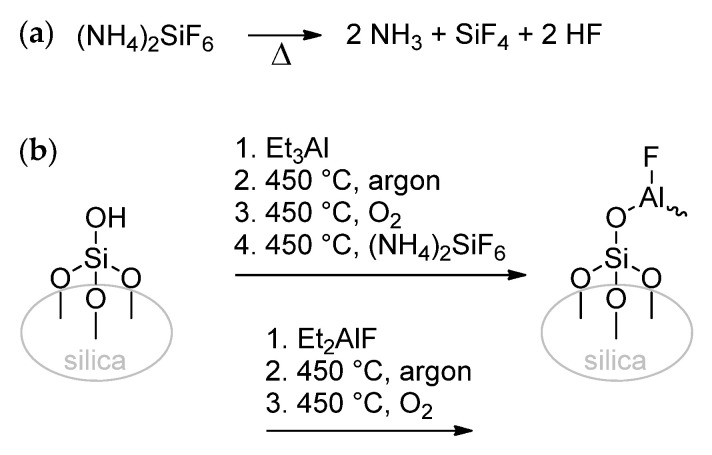
(**a**) Thermal decomposition of (NH_4_)_2_SiF_6_; (**b**) preparation of SiO_2_/g-Al_2_O_3_(F) [107].

The method of the preparation of SiO_2_/g-Al_2_O_3_(F) from silica, developed by Elf Atochem/Total, comprises the following stages (Scheme 18):

1. Treatment of silica with organoaluminum compound in the inert organic solvent;
2. Calcination of the product, at the final stage—in the presence of O_2_;
3. Fluorination using (NH_4_)_2_SiF_6_ and final calcination.

Fluorination is based on the reaction of HF, which was released at a high temperature (typically 450 °C) from the decomposition of the (NH_4_)_2_SiF_6_. An alternative method of fluorination is based on the reaction of silica with Et_2_AlF, followed by calcination. When using Et_2_AlF, there is no need for fluorination by (NH_4_)_2_SiF_6_; a sufficient amount of fluorine is introduced at the stage of the reaction between silica and Et_2_AlF.

An alternative approach to SiO_2_/g-Al_2_O_3_(F) was developed by INEOS [99,100]. The method of INEOS is based on the reaction of SiO_2_ with fluorinated alkoxides of alkylaluminum, followed by calcination (see below).

**Scheme 19:**
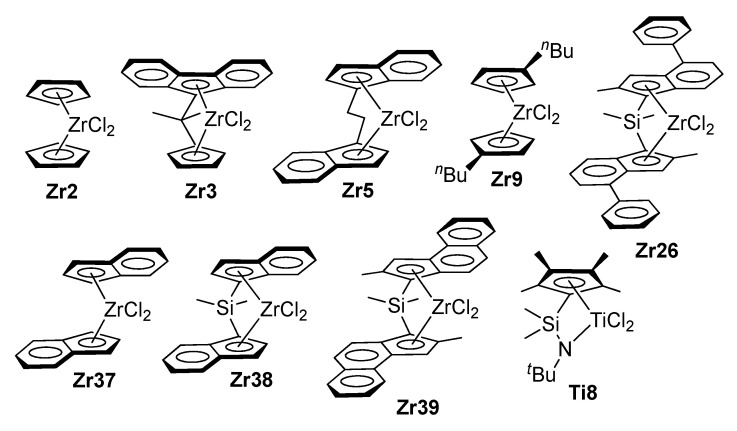
Group 4 metal complexes, investigated with SiO_2_/g-Al_2_O_3_(F) activating supports.

In Table 7, we tried to summarize the data on the synthesis of SiO_2_/g-Al_2_O_3_(F) supports and the results of their use for activation of Group 4 metal complexes in α-olefin polymerization. In addition to the zirconocenes **Zr2**, **Zr3**, **Zr5**, **Zr9**, **Zr26**, and **Zr37** and constrained-geometry complex **Ti8**, already mentioned in our review, the complexes *rac*-[Me_2_Si(η^5^-inden-1-yl)]ZrCl_2_ (**Zr38**) and *rac*-[Me_2_Si(η^5^-2-Me-4,5-benzoinden-1-yl)]ZrCl_2_ (**Zr39**) have been studied. The structures of complexes, investigated in combination with SiO_2_/g-Al_2_O_3_(F) activating supports, are presented in Scheme 19.

Polymerization experiments with **Zr5** were conducted at 80 °C with ethylene solutions in *n*-heptane (6 wt%); the [^i^Bu_3_Al]/[Zr] ratio was ~2000 [105]. Ethylene/hex-1-ene copolymerizations were also performed; the activities of supports have demonstrated similar trends. In both series of experiments, catalytic activities depended significantly on the types of silica used for the preparation of SiO_2_/g-Al_2_O_3_(F). If the average pore diameter was too small (e.g., support based on silica SIL 3), there was no activity. However, no clear correlations between silica specific surface/pore volume and catalytic activity have been found. The comparison of polymerization kinetic curves for **Zr5** supported on SiO_2_/g-Al_2_O_3_(F) and on solid MAO (SMAO) showed noticeable deactivation of the catalysts prepared with activating supports [105].

The results of the study of SiO_2_/g-Al_2_O_3_(F) with different fluorine contents (Table 3) as activating supports for **Zr5** and **Zr39** were reported by Boisson’ group in 2013 [107]. The results of four comparative polymerization experiments are included in Table 7; however, a number of the findings of this comprehensive study deserve a separate discussion. As shown in the Table 8, a significant variation of the Al content was detected for SiO_2_/g-Al_2_O_3_(F), whereas increasing the amount of (NH_4_)_2_SiF_6_ from 5 wt% to 20 wt% led to higher F content in the support. The treatment of silica with a higher amount of (NH_4_)_2_SiF_6_ resulted in a degradation of the silica grain. 

**Figure 19:**
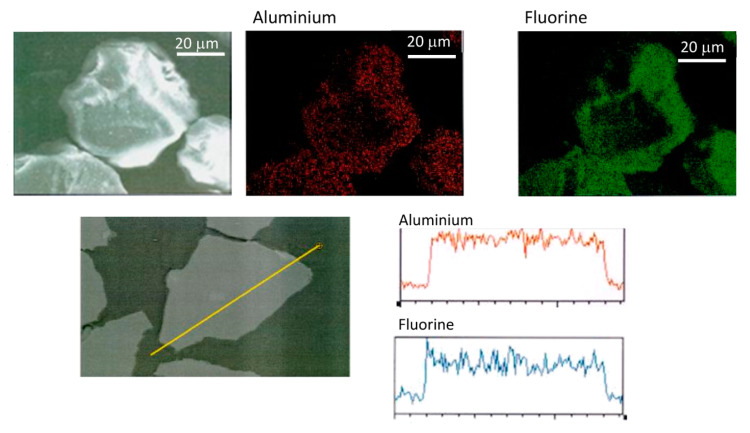
EDX analysis of the support AS1. Reprinted with permission from [107]. Copyright (2013) American Chemical Society.

As can be seen in Scheme 18, SiO_2_/g-Al_2_O_3_(F) should contain Al–F bonds; however, the formation of Si–F bonds on the surface of SiO_2_/g-Al_2_O_3_(F) is theoretically possible. Figure 19 shows that the spatial distribution of Al and F for the support AS1 (see Table 8) appears homogeneous; therefore, F atoms are mainly bonded to Al atoms. ^27^Al MAS NMR analysis of AS2, AS3, and AS4 after exposure to wet air showed road resonances at 50, 32, and 3.5 ppm for AS2 and the appearance of the signal at −15 ppm for AS3. This signal can be attributed to AlF_3_∙3H_2_O (the reference spectrum of this complex was also recorded). The signal at −15 ppm became the main for AS4. ^19^F MAS NMR spectrum of AS7 showed the presence of the signal of AlF_3_∙3H_2_O (−8 ppm) and the intence signal at 12 ppm. This last resonance is close to the signal assigned to ^VI^Al(O_5_F) (9 ppm) and ^VI^Al(O_4_F_2_) (20 ppm) by Fischer et al. [213]. 

**Figure 20:**
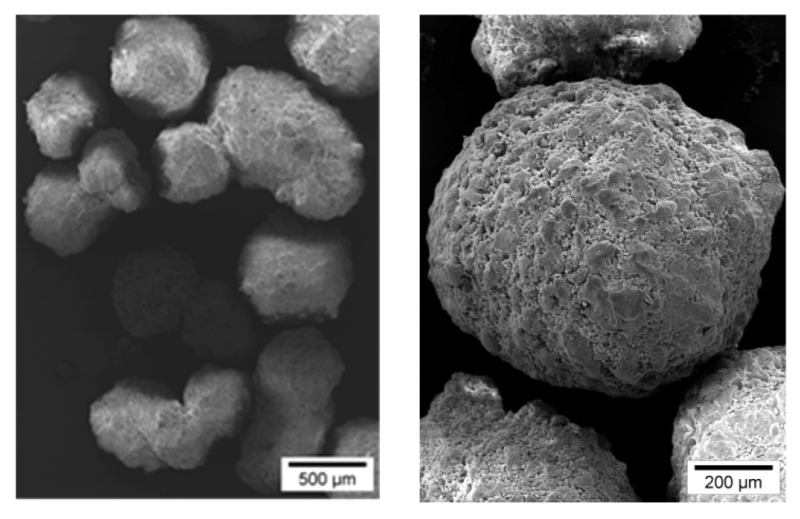
Polyethylene particles obtained using the catalyst **Zr5**/^i^Bu_3_Al/AS8 (heptane, 10 bar, 6 mL of hexene, 80 °C). Reprinted with permission from [107]. Copyright (2013) American Chemical Society.

The support AS1 was studied as an activator for the **Zr5**/^i^Bu_3_Al system in ethylene polymerization and ethylene/hex-1-ene copolymerization (~20 mg AS1, 0.5 μmol **Zr5**, 1 mmol ^i^Bu_3_Al, 4 bar ethylene, 80 °C). High activities (3.4–110 kg∙mmol^−1^∙h^−1^, 39–900 g∙g_Cat_^−1^∙h^−1^) and a remarkable hex-1-ene activation effect were observed. *T*_m_ of polymer decreased from 133 °C (PE) to 104 °C (copolymer with 56 mol% of hex-1-ene). Copolymerization experiments with AS2–AS7 supports and **Zr5**/^i^Bu_3_Al showed a high increase in activity as the wt % of F is increased on the carrier. The support AS5 prepared using treatment of the silica with Et_2_AlF showed comparable properties to support AS2 made using 5 wt % of (NH_4_)_2_SiF_6_, which had a similar content of F. In separate polymerization tests at 10 bar of ethylene (500 mL of heptane, 1 μmol **Zr5**, 2 mmol ^i^Bu_3_Al, 73 mg of AS8, 80 °C, 6 mL of hex-1-ene), productivity of 3200 g∙g_Cat_^−1^∙h^−1^ was achieved without the catalyst’ leaching, as evidenced by perfect morphology of the polymer obtained (Figure 20). 

During the comparison of **Zr5**/AS/^i^Bu_3_Al and **Zr5**/MAO catalysts, better incorporation of hexene was observed with the activating support. MWD and TREF analysis data indicated the presence of multiple active sites for the **Zr5**/AS/^i^Bu_3_Al catalytic system. Similar comparative studies were also performed for other Group 4 metal complexes **Zr9**, **Zr26**, **Zr3**, and **Ti8** (Scheme 19) with the use of supports AS6 and AS7 (Table 9). 

The complex **Zr9** showed a poor ability to insert hexene into the solution, but its behavior was highly improved when using the activating support. It should also be noted that activities (per mmol) of the ^i^Bu_3_Al/AS-activated complexes **Zr3** and **Ti8** in ethylene/hex-1-ene copolymerization were higher than activities of **Zr3**/MAO and **Ti8**/MAO. Better incorporation of hex-1-ene was also detected in all experiments with SiO_2_/g-Al_2_O_3_(F).

At first glance, SiO_2_/g-Al_2_O_3_(F) has demonstrated promising characteristics of the activating supports for zirconocenes; the catalysts obtained could have competed with conventional SiO_2_/MAO supports. However, more rapid decay of the polymerization rate when using **Zr5** on an activating support in comparison with **Zr5**/SMAO [105] has encouraged the research group of Boisson to perform a more thorough study of the kinetic behavior of SiO_2_/g-Al_2_O_3_(F)-activated **Zr5** and **Zr9** in the polymerization of ethylene [106]. In this study, three types of silica (Table 10) were used for the preparation of activating supports by the method described in [107]. The properties of fluorinated support M3703/079A, prepared from Grace Sylopol 952, are presented in the fourth row of Table 10. The complexes were pre-contacted with the supports for 10 min before introduction in the reactor. In all the reactions, around 60 mg of AS was used and the content of zirconocene was 0.4 wt%. Polymerization experiments were conducted at 80 °C and an ethylene pressure of 10 bar, in 0.1M hex-1-ene solution in *n*-heptane.

The study of the evolution of the *M*_w_ as a function of the reaction time using **Zr5**/AS/^i^Bu_3_Al showed that the *M*_w_ and *Ð*_M_ decrease as the reaction rate progresses. From the deconvolution results, it was shown that the rate of the formation of high-MW fraction tends to decrease significantly after 15 min of reaction, which corresponds to the beginning of the catalyst deactivation. Replacement of ^i^Bu_3_Al by Et_3_Al had no effect. However, **Zr9** seemed to respond quite differently to ^i^Bu_3_Al and Et_3_Al. In the absence of a sufficient quantity of alkylaluminum, the catalyst became active, but rapidly reached a maximum rate that was followed by total deactivation of in 20–30 min. In the case of ^i^Bu_3_Al as a co-catalyst, the addition of approximately 500–1000 mol Al/mol Zr was sufficient to stabilize the reaction rate at an intermediate value close to the maximum rate for an hour. These general trends were also observed with Et_3_Al as a co-catalyst to **Zr9**, although as with the **Zr5**, much less Et_3_Al was required than ^i^Bu_3_Al. Also, in the case of Et_3_Al, the stable reaction rates that were obtainable were higher than those that were realized with ^i^Bu_3_Al. However, an increase in the concentration of Et_3_Al has led to a significant decrease in activity due to the formation of heterobimetallic dormant species [106]. The results of the comparative study of two common pre-catalysts **Zr5** and **Zr9** showed that the influence of the pre-catalyst nature on catalytic properties of the complex system, comprising Group 4 metal complex, SiO_2_/g-Al_2_O_3_(F) and alkylaluminum are still not understood.

Besides the η^5^-ligand (L) environment in L_2_ZrX_2_, the nature of X can also affect the catalytic activity. Therefore, for example, [EBTHI]ZrF_2_ (**Zr1″**) demonstrated higher activity than **Zr1** and [EBTHI]ZrMe_2_ (**Zr1′**) under the same reaction conditions [103].

**Scheme 20:**
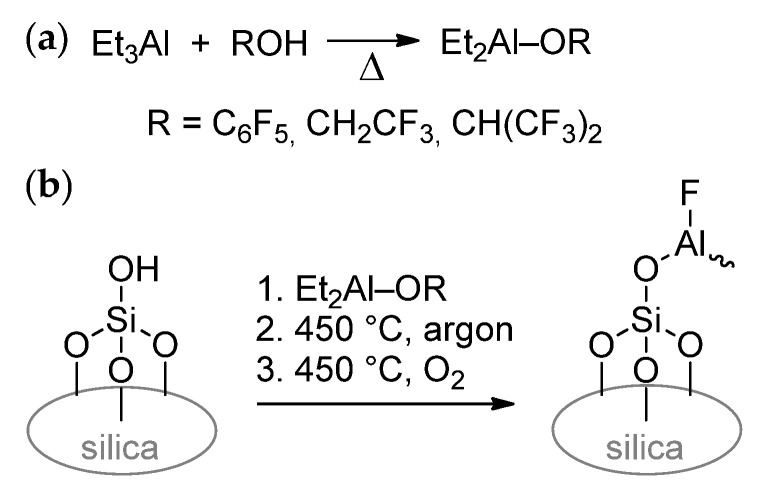
Preparation of SiO_2_/g-Al_2_O_3_(F) activating supports by the method of INEOS [99]. (**a**) Preparation of fluorinated alkoxide or aryloxide; (**b**) Treatment of the silica surface followed by calcination in an inert atmosphere and in the air.

The high efficiency of SiO_2_/g-Al_2_O_3_(F) activating supports caused competing polyolefin companies to search for alternative synthetic approaches to these phases to circumvent the Elf Atochem/Total patents. Such a search had proved successful during the studies of the chemists of INEOS who proposed the reaction of silica with Et_2_Al(OR) (R = C_6_F_5_, CH_2_CF_3_, CH(CF_3_)_2_), followed by calcination, as an alternative synthetic pathway to SiO_2_/g-Al_2_O_3_(F) [99] (Scheme 20). The [F]/[Al] ratios in these supports amounted to 1.96–2.26; these values were determined by the temperature of the pre-calcining of SiO_2_ (CS2050 from PQ Corp.) in the range of 250–450 °C.

These supports were compared with activating support SiO_2_/g-Al_2_O_3_(F), which was prepared by the method of Total with the use of (NH_4_)_2_SiF_6_ ([F]/[Al] = 1.68) [217], in copolymerization of ethylene and hex-1-ene in slurry and gas-phase experiments. Constrained-geometry complex (C_5_Me_4_SiMe_2_N*^t^*Bu)Ti(η^4^-1,3-pentadiene) (**Ti8″**) was selected as a pre-catalyst. In slurry polymerization experiments, **Ti8″** was pre-mixed with ^i^Bu_3_Al ([Al]/[Ti] = 50) and added to 0.1 g of SiO_2_/g-Al_2_O_3_(F) (Ti loading of 30 μmol/g_AS_), and polymerization was conducted in 1.7 L of isobutane containing 0.25 mmol ^i^Bu_3_Al at 90 °C; an H_2_/ethylene ratio of 0.2 mol% and ethylene pressure of 10 bar were maintained for 1 h. The samples of SiO_2_/g-Al_2_O_3_(F), which were prepared with the use of Et_2_Al(OC_6_F_5_) and (NH_4_)_2_SiF_6_, have demonstrated close activities (2.0–3.0 kg_PE_∙g_Cat_^−1^∙h^−1^). Before gas-phase polymerization experiments, solid catalysts were prepared by the above-described pre-mixing of the catalyst components, followed by washing with *n*-hexane and drying in vacuo. H_2_/ethylene and hex-1-ene/ethylene ratios were maintained at 0.13 and 0.55 mol%, respectively, during polymerization at 80 °C and 10 bar. In these experiments, catalytic activities were close to 0.5 kg_PE_∙g_Cat_^−1^∙h^−1^ [99]. Comprehensive rheological studies of copolymers [100] showed that novel supported catalysts provide the formation of polymers with improved rheological properties. In particular, higher viscosities were observed at lower shear rates, providing better bubble stability and lower viscosities at higher shear rates, resulting in better processability in the extruder.

The further development of the activating supports of SiO_2_/g-Al_2_O_3_(F) type by INEOS [101] consisted of the treatment of calcined SiO_2_ (Sylopol 332, W. R. Grace & Co.) by 2,4,6-trimethylpyridine and POCl_3_, followed by vacuum-drying (and optionally additional heating at 130 °C) and high-temperature fluorination with (NH_4_)_2_SiF_6_. The comparison of these supports with SiO_2_/g-Al_2_O_3_(F), prepared by the method of Total [217], in activation of **Zr5** and **Ti8″** for ethylene/hex-1-ene copolymerization showed marked increase in catalytic activities: 927 vs. 340 g_PE_∙g_Cat_^−1^∙h^−1^ for **Zr5** and 500 vs. 410 g_PE_∙g_Cat_^−1^∙h^−1^ for **Ti8″**. As can be seen in Scheme 19, a relatively small number of Group 4 metal complexes have been studied in MAO- and borate-free ethylene (co)polymerization using SiO_2_/g-Al_2_O_3_(F) supports. It can be assumed that the studies of other pre-catalysts can provide a breakthrough in the further development of single-site supported catalysts, which are suitable for use in the polyolefin industry.

#### 5.2.3. Silylation of Alumina with Subsequent Fluorination (Al_2_O_3_/g-SiO_2_(F))

**Figure 21:**
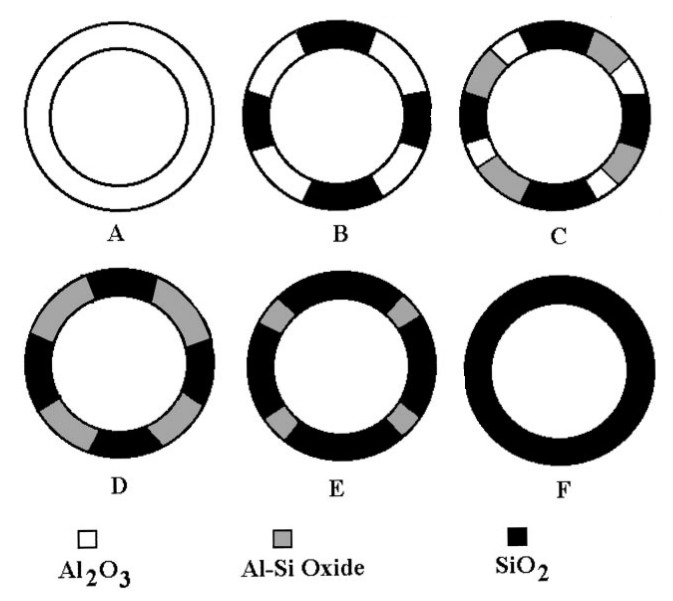
Model depicting the surface composition of PURAL SB (**A**), SIRALs 1.5–5 (**B**), SIRALs 10–20 (**C**), SIRALs 30–40 (**D**), SIRALs 60–80 (**E**), and SIRALs 90–100 (**F**). Reprinted with permission from [218]. Copyright (2000) Elsevier B. V.

‘Inverted’ phase Al_2_O_3_/g-SiO_2_ can be prepared, for example (so-called ‘SIRAL’ phases), by the hydrolysis of a solution of aluminum hexanolate in hexanol with deionized water at 90 °C, followed by mixing the filtered alumina suspension with a solution of orthosilicic acid, and spray-drying. Hydrothermal treatment (5 h at 180 °C) before spray-drying results in the formation of phases with higher acidity [218]. Modification of alumina through silica addition creates (i) strong LAS through isomorphous substitution of tetrahedral Si^4+^ by Al^3+^ ions and (ii) a small quantity of highly acidic BAS, which are thought to be bridged OH species similar to those found in zeolites [218]. The surface structures of SIRALs with different [Al]/[Si] ratios are schematically presented in Figure 21.

**Scheme 21:**
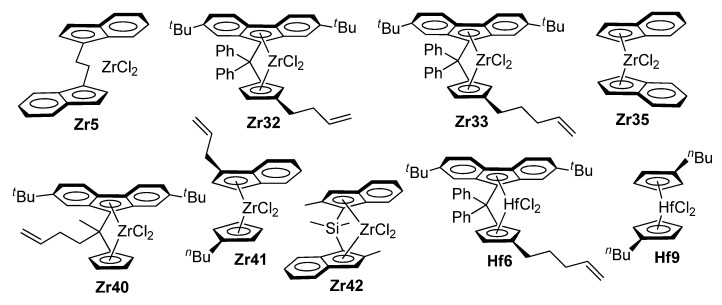
Group 4 metallocene pre-catalysts studied in olefin polymerization with the use of Al_2_O_3_/g-SiO_2_(F) activating supports.

In the first patent of Chevron Phillips in this field [94], Al_2_O_3_/g-SiO_2_(F) was prepared by the impregnation of commercial SIRAL 28M (Sasol) Al_2_O_3_/g-SiO_2_ with NH_4_HF_2_ in methanol with subsequent calcining. In ethylene polymerization on **Zr32**/^i^Bu_3_Al, Al_2_O_3_/g-SiO_2_(F) provided better activity; it was far superior to that of Al_2_O_3_/SiO_2_(F) and almost twice that of sulfated alumina [94]. In ethylene homopolymerization on **Zr40** (Scheme 21), activated by ^i^Bu_3_Al, catalytic activity depended on the alumina to silica ratio, varying from 1.69 kg_PE_∙g_Cat_^−1^∙h^−1^ (fluorinated γ-Al_2_O_3_) to 7.30 kg_PE_∙g_Cat_^−1^∙h^−1^ (Al_2_O_3_/g-SiO_2_(F) with Al_2_O_3_/SiO_2_ = 1.5:1 by weight) [94]. Undoubtedly of interest are the results of the comparative study of catalytic activity of **Zr5**/^i^Bu_3_Al and **Zr40**/^i^Bu_3_Al on different activating supports in ethylene/hex-1-ene copolymerization (Table 11).

This table also contains the tan δ (the ratio of loss and storage moduli, determined at 190 °C by small-strain (10%) oscillatory shear measurements, the shear frequency of 0.1 s^−1^) related to the presence of long-chain branches (LCB) in copolymer (higher tan δ means that the polymer relaxes easily, with little storage of the strain, and that the polymer has relatively lower LCB). 

The synthesis of Al_2_O_3_/g-SiO_2_(F) having varied [Al]/[Si] ratios was also described in [94]. Pre-calcined (600 °C) Alumina A (W. R. Grace & Co., surface area ~300 m^2^∙g^−1^, pore volume 1.3 mL∙g^−1^) was treated with different amounts of Si(OEt)_4_ in MeOH and further with NH_4_HF_2_. The product was calcined in N_2_ at 600 °C. The highest activity for the **Zr40**/^i^Bu_3_Al system in ethylene polymerization (6.3 kg∙g_Cat_^−1^∙h^−1^) was observed for Al_2_O_3_/g-SiO_2_(F) with Al_2_O_3_/SiO_2_ ratio of 7.3 by weight.

Very high catalytic activities in the polymerization of ethylene were achieved for the **Zr40**/^i^Bu_3_Al system when using fluorinated-chlorinated silica-coated alumina as an activating support [96]. This support was prepared by the treatment of Alumina A (W. R. Grace & Co., surface area 300 m^2^∙g^−1^, pore volume 1.2 mL∙g^−1^) with Si(OEt)_4_/^i^PrOH, followed by drying and calcining at 500–900 °C. The chlorination stage (4 h) involved injecting and vaporizing CC1_4_ into the gas stream used to fluidize the Al_2_O_3_/g-SiO_2_ during calcination at 500 °C every five minutes. The fluorination step was conducted in a similar way using tetrafluoroethane within 4.5 h. The maximum activity of 17.83 kg_PE_∙g_Cat_^−1^∙h^−1^ was demonstrated by **Zr40**, which was activated by ^i^Bu_3_Al and Al_2_O_3_/g-SiO_2_(F,Cl) containing 4 wt% of Cl and 7 wt% of F at 95 °C and 28 bar of ethylene [96]. A higher efficiency of Al_2_O_3_/g-SiO_2_(F,Cl) in comparison with other activating supports in ethylene polymerization was also detected for **Zr32**, **Zr35**, and **Hf9** [98]. 

Another interesting application of the Al_2_O_3_/g-SiO_2_(F,Cl) support was the activation of the mixture of pre-catalysts **Hf6** and **Zr41** in ethylene/hex-1-ene copolymerization [96]. The amounts of 1.2 mg of **Hf6**, 1.4 mg of **Zr41**, and 150 mg of support were used, and copolymerization was conducted under similar conditions, with the addition of 5 g of hex-1-ene and 175 ppm of the molecular hydrogen. In this experiment, the catalytic activity of 2.19 kg_PE_∙g_Cat_^−1^∙h^−1^ was achieved. In propylene polymerization experiments (70 °C, 31 bar) using the **Zr42**/^i^Bu_3_Al/Al_2_O_3_/g-SiO_2_(F,Cl) system, catalytic activities reached up to 5.6 kg_PP_∙g_Cat_^−1^∙h^−1^ [96].

Particularly noteworthy are relatively recent studies of ‘mixed’ catalytic systems, conducted by the chemists of Chevron Phillips [219,220]. The very idea of these studies was to develop a heterogeneous system containing both Ziegler–Natta and zirconocene catalysts. As described in [219,220], Al_2_O_3_/g-SiO_2_(F) was prepared from Alumina A by treatment with Si(OEt)_4_/^i^PrOH, followed by drying, calcining at 600 °C, fluorination by NH_4_HF_2_/MeOH, and calcination. The Ziegler–Natta catalyst was prepared by the reaction of Al_2_O_3_/g-SiO_2_(F) with Bu_2_Mg (toluene, 90 °C, 3 h), followed by treatment with TiCl_4_ (90 °C, 3 h). Activation of zirconocenes **Zr33**, **Zr40**, and **Zr41** by ^i^Bu_3_Al and this support gave highly efficient catalysts of the copolymerization of ethylene with hex-1-ene [219,220]. When using **Zr40**/^i^Bu_3_Al at 90 °C and 27 bar of ethylene, catalytic activities were 49.4 and 44.9 kg∙g_Cat_^−1^∙h^−1^ in the absence and in the presence (880 ppm) of the molecular hydrogen. In the absence of zirconocene, Al_2_O_3_/g-SiO_2_(F)-based Ziegler–Natta catalyst was less active (26.5 and 10.8 kg∙g_Cat_^−1^∙h^−1^ in the absence and in the presence (1000 ppm) of the H_2_).

Another method of the preparation of ‘Ziegler–Natta’ Al_2_O_3_/g-SiO_2_(F) catalysts/supports was based on the interaction of Al_2_O_3_/g-SiO_2_(F) with solutions of active metal chlorides and MgCl_2_ in THF, followed by centrifugation and drying in vacuo [219,220]. When TiCl_4_ was used as active metal chloride, a very active catalyst was obtained (5.27 kg∙g_Cat_^−1^∙h^−1^); however, after absorption of the **Zr40**/^i^Bu_3_Al catalytic, activity tripled (~15 kg∙g_Cat_^−1^∙h^−1^). Zirconocenes **Zr33** and **Zr41** were studied in combination with this type of active support with the aim of obtaining polyethylenes having a broad spectrum of characteristics. 

The third method of the preparation of Ti/Mg Al_2_O_3_/g-SiO_2_(F), proposed in [219,220], is close to the common method of the preparation of TMZNCs by the reaction of Mg(OEt)_2_ with TiCl_4_ [221]: Mg(OEt)_2_ reacted with Al_2_O_3_/g-SiO_2_(F) in toluene, with subsequent reflux with TiCl_4_. Zirconocenes **Zr33** and **Zr41** with this type of Ti/Mg Al_2_O_3_/g-SiO_2_(F) support have demonstrated maximum activities of 22.1 and 11.7 kg∙g_Cat_^−1^∙h^−1^, respectively, in copolymerization of ethylene with hex-1-ene in the presence of 100 ppm of H_2_. In almost all samples of TMZNC/zirconocene catalysts, supported on Al_2_O_3_/g-SiO_2_(F), the [Zr]/[Ti] ratio was ~3:10, from which it follows that the zirconocene component of the binary catalyst is far more active.

#### 5.2.4. Fluorinated SiO_2_/Al_2_O_3_ in Oligomerization of Higher α-Olefins

**Scheme 22:**
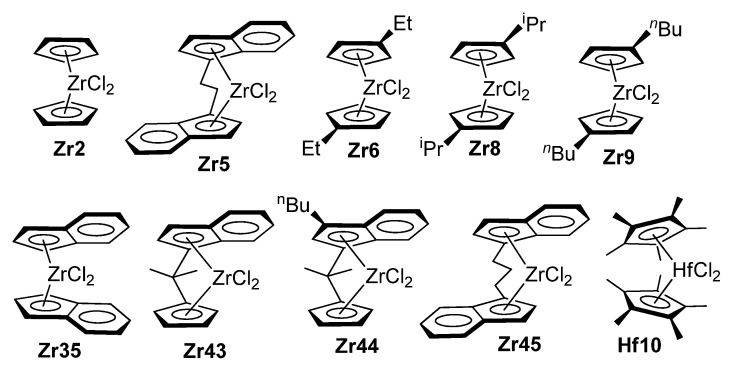
Group 4 metallocene pre-catalysts that have demonstrated high efficiency in the oligomerization of oct-1-ene when activated by SiO_2_/Al_2_O_3_(F) and ^i^Bu_3_Al [97].

SiO_2_/Al_2_O_3_(F), prepared by the ‘wet’ method from MS13-110 silica/alumina (W. R. Grace & Co., 13 wt% Al_2_O_3_, surface area 400 m^2^∙g^−1^, pore volume 1.2 mL∙g^−1^), was used as an activating support for the series of Group 4 metallocenes (17 examples) in a comparative study of the catalytic activity in oligomerization of oct-1-ene [97]. ^i^Bu_3_Al was used as an organoaluminum activator for L_2_MCl_2_ before the addition of the activating supports. The structures of the most active pre-catalysts are presented in Scheme 22; the results of the oligomerization experiments are given in Table 12. As can be seen from this table, TOF of 10^5^ h^−1^ and higher can be achieved when using SiO_2_/Al_2_O_3_(F), which is comparable to activities of the best oligomerization catalysts under homogeneous conditions with perfluoroborate activation [222,223].

However, this topic has not received further development. It can be assumed that SiO_2_/Al_2_O_3_(F)-based α-olefin oligomerization catalysts have a fundamental flaw resulting from the high acidity of the support. The consequence of such acidity is a possibility of cationic skeletal rearrangements during oligomerization, which leads to the deterioration of the characteristics of α-olefin oligomers in terms of their use for the production of engine oil feedstocks.

### 5.3. Fluorinated Compositions of SiO_2_ or Al_2_O_3_ with Other Metal Oxides

The preparation of fluorinated supports, based on silica and Group 4 metal oxides, was described in the patent of Phillips Petroleum [90]. The silica/titania, prepared by the cogellation method (8 wt% of TiO_2_, surface area of ~450 m^2^∙g^−1^) was calcined at 600 °C, treated with NH_4_HF_2_, and calcined at different temperatures. The temperature of the final calcination had a significant effect on the efficiency of these phases when being used as activating supports for the **Zr9**/Et_3_Al catalyzed polymerization of ethylene. Therefore, for example, when using supports calcined at 600 and 450 °C, catalytic activities were 1164 and 2837 g_PE_∙g_Cat_^−1^∙h^−1^_._ Note that fluorinated TiO_2_ had demonstrated the lack of activation in **Zr9**/Et_3_Al catalyzed polymerization of ethylene. Under the same conditions, the samples of fluorinated silica/zirconia have demonstrated a higher efficiency in comparison with fluorinated silica/titania (up to 5 kg_PE_∙g_Cat_^−1^∙h^−1^).

Impregnation of alumina by Zr(OBu)_4_ with subsequent calcination (dry N_2_) and fluorination by perfluorohexane at 600 °C resulted in the formation of fluorinated alumina/zirconia with low own activity in ethylene polymerization after treatment with Et_3_Al (35 g_PE_∙g_Cat_^−1^∙h^−1^) [91]. When using fluorinated alumina/zirconia as an activating support for **Zr9**, the catalyst productivity increased to 1.38 kg_PE_∙g_Cat_^−1^∙h^−1^, which was comparable to the efficiency of the fluorinated alumina support [91]. The presence of high-MW ‘Ziegler–Natta’ PE, formed on fluorinated alumina/zirconia catalytic centers, without the participance of metallocene, allows to consider the product of polymerization as a prospective PE composite.

### 5.4. Chlorinated Metal Oxides

Ketjen Grade B alumina and Davison Grade 952 silica were chlorinated by the reaction with CCl_4_ vapor at 600 °C. After the addition of a toluene solution of **Zr9** and Et_3_Al, a moderately active ethylene polymerization catalyst was formed by chlorinated alumina (~10^3^ g_PE_∙g_Cat_^−1^∙h^−1^), whereas chlorinated silica was found to be inactive [91]. Impregnation of alumina by Zr(OBu)_4_ with subsequent calcination (dry N_2_) and chlorination resulted in a solid phase, which was active in ethylene polymerization after treatment with Et_3_Al; the addition of **Zr9** resulted in a further increase of activity (862 and 1484 g_PE_∙g_Cat_^−1^∙h^−1^, respectively). The efficiency of chlorinated silica/zirconia support in the activation of **Zr9** was moderate [91].

The studies of other methods of the chlorination of HPV alumina were reported in the Chevron Phillips patent [92]; SO_2_Cl_2_ at 300 °C and AlCl_3_ at 250 °C were found to be fairly efficient chlorinating agents (activities of 459 and 401 g_PE_∙g_Cat_^−1^∙h^−1^ in ethylene polymerization when using **Zr9**/Et_3_Al). Chlorination of alumina by CCl_4_ under optimized conditions allowed to reach the catalytic activity of 2.8 kg_PE_∙g_Cat_^−1^∙h^−1^ for **Zr9**/Et_3_Al [92].

## 6. Other Inorganic Supports

There are only a few articles that address patents devoted to the study of inorganic activating supports distinct from the supports discussed above. In 1995, Soga and colleagues demonstrated the ability of solid heteropolyacids H_3_[PMo_12_O_40_] and H_5_[PMo_10_V_2_O_40_] to activate **Zr2** and **Zr5** in the polymerization of ethylene and propylene, respectively [224]. Catalytic activities were low (up to 23 and 3.7 kg∙mol_Zr_^−1^∙h^−1^). Dimethyl derivative **Zr2′** was inactive, on the basis of which they concluded that activation of zirconocene pre-catalyst L_2_ZrCl_2_ proceeds through Cl abstraction.

Impregnation of the silica, alumina, or SiO_2_/Al_2_O_3_ by different metal chlorides with subsequent calcination allowed to obtain solid supports with different activating efficiency [225,226,227]. Active supports were prepared from Ketjen Grade B alumina using Cu(II), Sn(IV), Ag(I), Nb(V), Mn(II), W(VI), La(III), Nd(III), Sb(V), Zn(II) [225], Mo(VI) [226], and Ni [227] salts. The activation of **Zr9**/Et_3_Al in comparative experiments on the homopolymerization of ethylene (38 bar, 90 °C) allowed the identification of the most efficient systems, which were based on Sn, Ag, and Zn chlorides. When the loading of ZnCl_2_ was 20 wt% of alumina, after calcining with CCl_4_ and impregnation with **Zr9**/Et_3_Al, the catalytic activity of 11.8 kg∙g_Cat_^−1^∙h^−1^ was achieved [225].

Cation-exchanged fluorotetrasilicic mica (M^n+^-mica, M^n+^ = Na^+^, Mg^2+^, Fe^3+^, Co^2+^, Ni^2+^, Cu^2+^, Zn^2+^) were prepared and employed as activating supports in the ethylene polymerization or ethylene/hex-1-ene copolymerization (7 bar, 60 °C) in the presence of L_2_ZrCl_2_/^i^Bu_3_Al [228]. M^n+^-mica were calcined at different temperatures, and 100 mg samples of the support were contacted with 20 μmol of **Zr2** or **Zr9** with subsequent treatment by ^i^Bu_3_Al. Catalytic activities in **Zr2** catalyzed homopolymerization of ethylene substantially depended on the nature of M^n+^ (Fe^3+^-mica demonstrated the highest productivity of 174 g_PE_∙g_Cat_^−1^∙h^−1^) and the temperature of calcination (200 °C was the optimal value). The authors proposed that the active sites were probably formed by the reactions of Cp_2_ZrCl_2_ and the exchanged cations M^n+^ since the activity was significantly increased by replacing Na^+^ ions with M^2+^ (M^2+^ = Mg^2+^, Co^2+^, Ni^2+^, Cu^2+^, Zn^2+^) and Fe^3+^. This work is of particular interest, but it is not applicable in practice due to the extremely low activity of the catalysts. 

## 7. Organochemical Functionalization of Inorganic Surfaces and Organic Polymers 

### 7.1. Organochemical Functionalization of Inorganic Phases

The very idea of low-cost surface modification of SiO_2_, Al_2_O_3_, SiO_2_/Al_2_O_3_, and other inorganic phases by C_6_F_5_Br and its analogs with a formation of perfluoroaryl-grafted oxides was patented by W. R. Grace & Co. in 1999 [229]. However, this patent did not include any data on catalytic activity. 

**Scheme 23:**
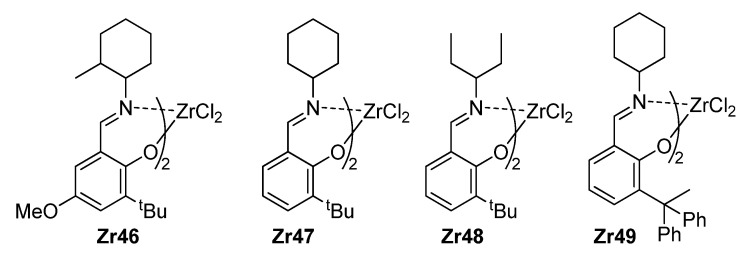
Zirconium complexes, studied in ethylene polymerization using (Ph_3_C)_m_H_n_[PMo_12_O_40_] as an activator [230].

In the early 2000s, the activation of post-metallocenes **Zr46**–**Zr49** (Scheme 23) by surface-modified heteropolyacids and Et_3_Al was studied by Fujita’s group [230]. The activating support of the formula (Ph_3_C)_m_H_n_[PMo_12_O_40_]∙8H_2_O (average m/n~2:1) was obtained by the reaction of H_3_[PMo_12_O_40_]∙28H_2_O with Ph_3_CCl in acetone with subsequent evaporation, washing by toluene, hexane, and drying at 100 °C under vacuum. After the addition of 10 μmol of Zr complex to the mixture of 5 μmol of the support and 1 mmol of Et_3_Al, fast ethylene polymerization was observed under atmospheric pressure and 25 °C, and activities of **Zr46**–**Zr49** were 1.02–5.64 kg∙mol_Zr_^−1^∙h^−1^; complex **Zr49** demonstrated the highest activity. In the cases of **Zr47** and **Zr48**, bimodal PEs were obtained, whereas complex **Zr49** catalyzed the formation of PE with abnormally narrow MWD (*Đ*_M_ = 1.45). The nature of the supports and activation mechanisms were not studied and discussed in [230]. One can assume that the reaction of (Ph_3_C)_m_H_n_[PMo_12_O_40_]∙8H_2_O with Et_3_Al results in the formation of soluble dimeric ethylalumoxane and Mo–O–AlEt_2_ species and does not affect Ph_3_C^+^—[PMo_12_O_40_]^m−^ interactions. Activation of L′ZrCl_2_ proceeds through alkylation by Et_3_Al and subsequent Zr→CPh_3_ ethyl transfer with a formation of L_2_ZrEt^+^ and 1,1,1-triphenylethane. This activation mechanism is very similar to the conventional mechanism of the activation of Group 4 metal complexes by [Ph_3_C]^+^[B(C_6_F_5_)_4_]^−^ [9]. 

**Scheme 24:**
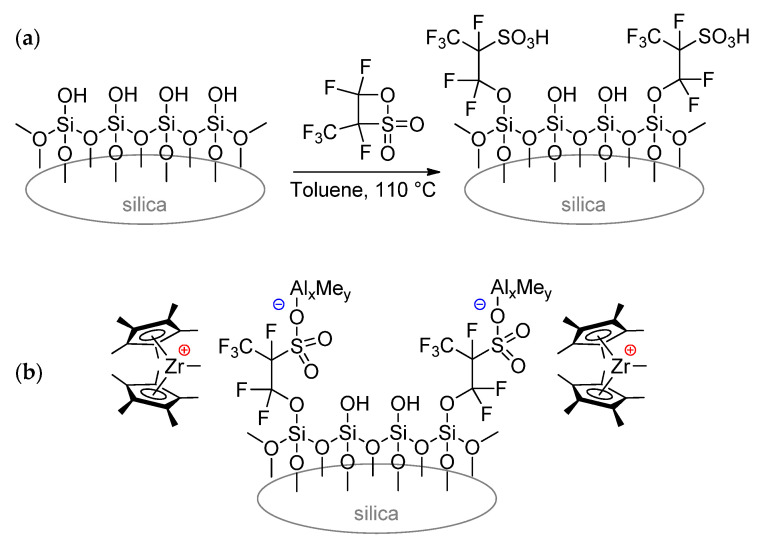
(**a**) Synthesis of tethered Brønsted acid sites by the reaction of silica with perfluorinated sultone; (**b**) hypothetical activated catalyst [231].

In 2007 [231], Jones and colleagues proposed the further development of the idea of solid activating supports, which consisted in surface modification of silica (mesoporous SBA-15) by perfluoroalkyl–SO_3_H groups using fluorinated sultone precursor [232] (Scheme 24a). In the presence of Me_3_Al, this support activated pre-catalyst **Zr17′** in ethylene polymerization (25 °C, 4 bar), yielding productivities up to 1000 kg_PE_∙mol_Zr_^−1^∙h^−1^ without reactor fouling. During the study of the activation mechanism, possible ways of MAO formation and direct interaction of –SO_3_H groups with **Zr17′** were ruled out, which led to the structure of the activated catalyst presented in Scheme 24b. 

**Scheme 25:**
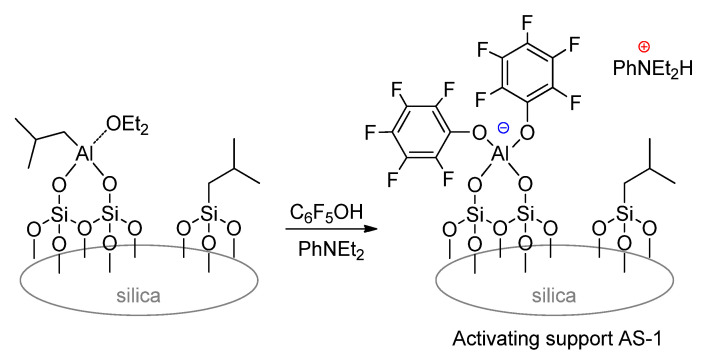
Preparation of a bis(pentafluorophenoxy)aluminate activator [54].

In 2011, Taoufik and colleagues showed that the treatment of silica surface with ^i^Bu_3_Al in Et_2_O afforded a well-defined bipodal surface species [(≡SiO)_2_Al(^i^Bu)(Et_2_O)] [149] (Scheme 25). In further studies, this material was treated with C_6_F_5_OH (2.5 eq.) in benzene in the presence of one eq. of *N*,*N*-diethylaniline; repeated washing and vacuum-drying resulted in obtaining of activating support AS-1 (Scheme 25) [54]. The structure of the surface species was confirmed by diffuse reflectance infrared Fourier transform (DRIFT) spectroscopy and ^1^H, ^13^C, ^19^F, and ^27^Al CP-MAS NMR. 

The catalytic performance of the support was studied in ethylene polymerization with and without hex-1-ene, using common **Zr5** and **Zr9** pre-catalysts and ^i^Bu_3_Al. Reference tests were performed with silica-supported MAO (sMAO). The results of the experiments are presented in Table 13 [54]. 

The **Zr5**/AS-1 system was found to be more active in comparison with **Zr5**/sMAO; a major comonomer effect was observed. The initial activity was higher when using the activating support AS-1, but deactivation was observed during the course of polymerization. This deactivation could be ascribed to aryloxide transfer to Zr.

**Scheme 26:**
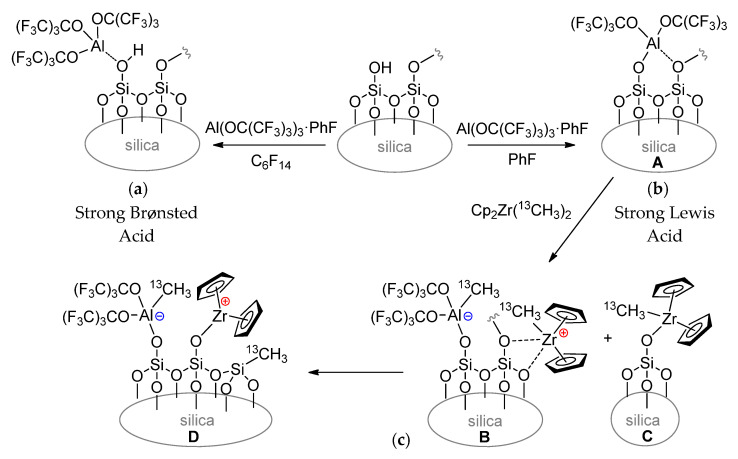
(**a**) Formation of Brønsted (**a**) and Lewis (**b**) acidic sites during the reaction of Al(OC(CF_3_)_3_)_3_∙PhF with silica; (**c**) activation of Cp_2_Zr(^13^CH_3_)_2_ [234]. The key intermediates **A**, **B**, **C** and **D** are discussed below.

During the search of weakly coordinating surface anions, Conley and colleagues studied the reaction of Al(OC(CF_3_)_3_)_3_∙PhF with calcined silica in perfluorohexane that resulted in the formation of highly acidic [≡SiOH^…^ Al(OC(CF_3_)_3_)_3_] species [233] (Scheme 26a). Two years later, they published the results of further studies of this reaction [234]. It turned out that the reaction of Al(OC(CF_3_)_3_)_3_∙PhF with partially dehydroxylated silica in fluorobenzene solvent, which induces proton transfer, results in the formation of [≡SiOAl(OC(CF_3_)_3_)_2_(O(Si≡)_2_) species (**A** in Scheme 26b) with extremely high Lewis acidity.

The ^27^Al{^1^H} MAS NMR spectrum of **A** contained a single signal that simulates as an isotropic chemical shift (δ_iso_) of 74 ppm and a quadrupolar coupling constant (C_Q_) of 18.0 MHz; DFT optimization of **A** predicted C_Q_ = 18.7 MHz. The calculated fluoride ion affinity (FIA) for **A** was 528 kJ∙mol^−1^, which is significantly larger than the calculated FIA for B(C_6_F_5_)_3_ (448 kJ∙mol^−1^. The reaction of **A** with Cp_2_Zr(^13^CH_2_)_2_ was complex. A ^13^C{^1^H} CP-MAS NMR spectrum of Cp_2_Zr(^13^CH_3_)_2_/**A** contains four signals at 38 (Cp_2_Zr–^13^CH_3_^+^ in species **B**, Scheme 26b), 23 (species **C**), 3 (Si–^13^CH_3_ in species **D**), and –11 (Al–^13^CH_3_ in species **B** and **D**) ppm. The pressurizing of Cp_2_Zr(^13^CH_3_)_2_/**A** in cyclohexane by 20 bar of ethylene resulted in the formation of PE with an activity of 78.6 g_PE_∙g_Cat_^−1^∙h^−1^ [234]. The findings of this study are of great significance for understanding the chemistry of Si/Al-based activating supports and for developing new-generation MAO- and borate-free supported Group 4 metallocene and post-metallocene catalysts. 

### 7.2. Functionalized Organic Polymers

**Scheme 27:**
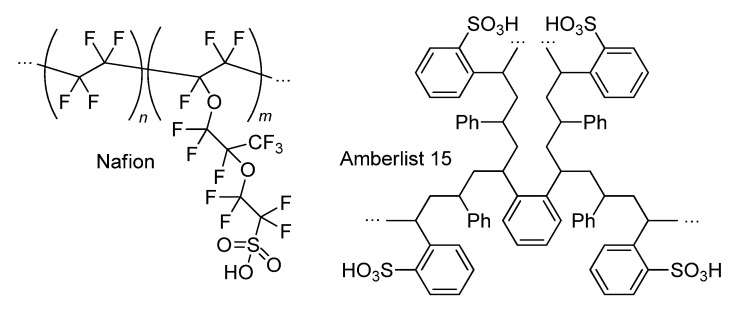
Structures of sulfated polymers used as activating supports for the **Zr5**/^i^Bu_3_Al system [236].

Nafion (Scheme 27) is the first of a class of synthetic polymers with ionic properties; its molecular structure comprises poly(tetrafluoroethylene) backbone and perfluorovinyl ether groups terminated with sulfonate groups as side chains [235]. The presence of highly acidic –SO_3_H groups and permeation for gases led the chemists of Mitsui Petrochemicals to the suggestion that Nafion can be used as an activating support for L_2_ZrCl_2_/R_3_Al systems [236]. This assumption was confirmed by ethylene polymerization experiments (75 °C, pressure not indicated) using **Zr5**/^i^Bu_3_Al; however, substantially cheaper sulfated polymer Amberlist (Scheme 27) showed comparable ability to activate zirconocene. The maximum activity of this catalyst can be roughly estimated as ~2 kg∙g_Cat_^−1^∙h^−1^. Note that MgCl_2_-supported **Zr5**/^i^Bu_3_Al under the same conditions was about an order of magnitude less active [236]. 

## 8. Ionic Liquids

Ionic liquids (ILs) have long attracted the attention of researchers as carrier phases for different catalysts [237]. If the reaction proceeds in a non-polar medium, the use of ILs simplifies the separation of the products and recycling of the catalysts. In particular, ILs have already found applications in the cationic oligomerization of α-olefins [238,239,240,241,242]. Polymerization of ethylene and α-olefins with the use of coordination catalysts and ILs can be considered to be a heterogeneous process. The term ‘support’ implies belonging to the solid phase, so that the inclusion of ILs in this review is not entirely appropriate. However, ILs represent a convenient reaction medium for the study of activation processes, and we chose to include this short section in the present review. 

In 1990 [243], Carlin and Wilkes reported the results of the study of ambient-temperature chloroaluminate molten salt AlCl_3_∙MEIC (MEIC—1-ethyl-3-methylimidazolium chloride) as applied to activation of **Ti2**. When bubbling ethylene at 1 atm through 1.1:1.0 AlCl_3_∙MEIC (6.8 g) containing 22 mg **Ti2** and 0.118 g Al_2_Me_3_Cl_3_, 20 mg PE was obtained. Similar Zr (**Zr2**) and Hf (**Hf1**) complexes were inactive under similar conditions. The authors proposed the formation of the species of the formula Cp_2_TiMe-(μ-Cl)-AlCl_3_ that are able to dissociate with a formation of active Cp_2_TiMe^+^ species. The inertness of **Zr2** and **Hf1** was attributed by the authors to the higher strength of the M–Cl bond for M = Zr, Hf in comparison with Ti, but this explanation contradicts some reports, e.g., [244]. 

**Figure 22:**
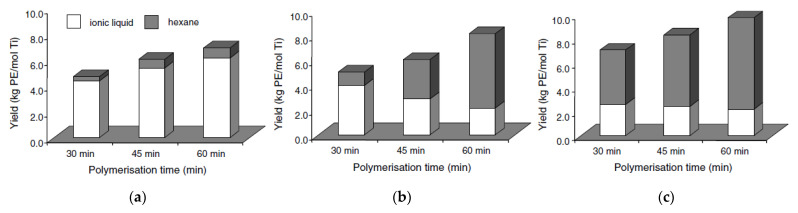
Influence of the kind of alkylaluminum compound and the polymerization time on the PE yield obtained using the Cp_2_TiCl_2_ catalyst in biphasic [BMIM]^+^[AlCl_4_]^−^/hexane polymerization. The PE yield is presented separately for both phases. The alkylaluminum compound: (**a**) MAO, (**b**) Et_2_AlCl (ionic liquid)/MAO (hexane), (**c**) AlEt_2_Cl. Polymerization condition: Ti = 0.06 μmol, Al = 4 mmol, 1 bar. Reprinted with permission from [245]. Copyright (2007) Springer Nature.

From 2007 to 2013, a number of articles on the subject of metallocene activation using ILs were published by the research group of Ochędzan-Siodłak [245,246,247,248,249]. In [245], the catalytic behavior of **Ti2** in ethylene polymerization with 1-*n*-butyl-3-methylimidazolium tetrachloroaluminate [BMIM]^+^[AlCl_4_]^−^ when using MAO, Et_2_AlCl, and Et_3_Al as alkylating agents was investigated. During polymerization experiments (*n*-hexane, 30 °C, 1 bar), a marked difference in the distribution of PE between IL and *n*-hexane phases was observed (Figure 22). At the beginning of the polymerization, the ionic liquid phase becomes white and swells considerably as the polyethylene appears. After 5–10 min, the hexane becomes a white suspension as the polyethylene is progressively shifted from the ionic liquid phase. The best results were obtained using Et_2_AlCl, where the greatest amount of the polymer is in the hexane phase, whereas the amount of the polymer in the ionic liquid decreases and slowly reaches a constant level. After separation, this [BMIM]^+^[AlCl_4_]^–^/**Ti2**/Et_2_AlCl phase maintained the ability to catalyze ethylene polymerization, at 30 °C and 1 bar activities, based on the weights of PE collected from the *n*-hexane phase, which were 9.0 and 8.8 kg_PE_∙mol_Ti_^−1^∙h^−1^ for freshly prepared and recycled catalysts, respectively. When using 1-ethyl-3-methylimidazolium tetrachloroaluminate and **Ti2**, the distribution of PE significantly deteriorated; even in the presence of Et_2_AlCl, the main fraction of PE remained in the IL phase [246].

**Scheme 28:**
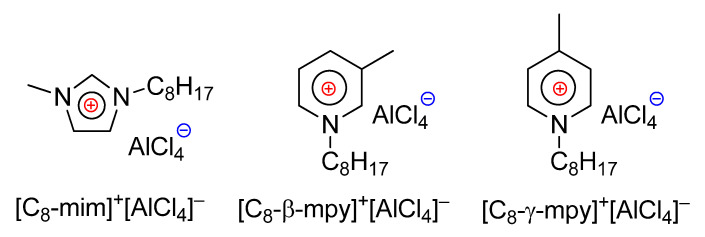
Structures of ionic liquids studied as activators for **Ti2**/EtAlCl_2_ in the polymerization of ethylene [247].

The yield and distribution of PE between IL and aliphatic phases substantially depended on the nature of IL and the duration of polymerization. Comparative studies of ILs [C_8_-mim]^+^[AlCl_4_]^−^, [C_8_-β-mpy]^+^[AlCl_4_]^−^, and [C_8_-γ-mpy]^+^[AlCl_4_]^−^ (Scheme 28) as activators for **Ti2**/EtAlCl_2_ in ethylene polymerization showed the advantage of pyridinium-based ILs both in activity and in PE distribution at a low Al/Ti ratio of 67 (Table 14) [247]. The best results were obtained with use of [C_8_-mim]^+^[AlCl_4_]^−^, for which the optimal Al/Ti molar ratio was 133. In all experiments, the use of the larger amounts of the EtAlCl_2_ activator had a disadvantageous impact on the polymer transfer to the hexane phase. It was also shown that an active catalyst was formed and immobilized in IL phases, and no catalyst leakage was observed [247].

The replacement of *n*-octyl substituent in [C_8_-mim]^+^[AlCl_4_]^–^ by the PhCH_2_CH_2_– fragment was found to be suitable to immobilize **Ti2**/EtAlCl_2_, and PE was gathered mainly in the hexane phase [248]. An increase in the reaction time resulted in a considerable increase in the amount of PE and improved the PE transfer from the IL to hexane. Also, PhCH_2_CH_2_-substituted IL was more stable at high temperatures in comparison with [C_8_-mim]^+^[AlCl_4_]^−^.

**Scheme 29:**
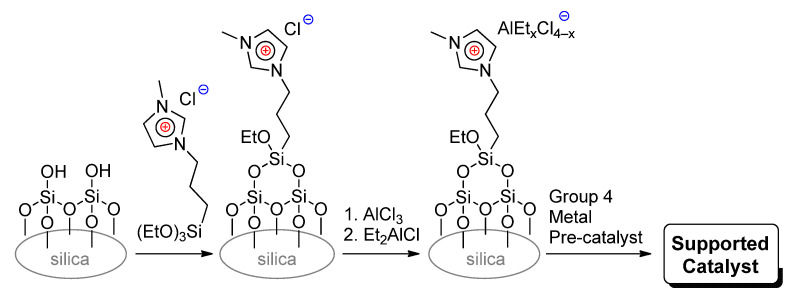
Structures of ionic liquids studied as activators for **Ti2**/EtAlCl_2_ in the polymerization of ethylene [247].

In 2014, Ochędzan-Siodłak and Dziubek came up with the idea of the immobilization of Group 4 metal catalysts on IL-modified silica (SIL) [249]. The modification of SiO_2_ was based on the reaction of surface Si–OH groups with (EtO)_3_Si-finctionalized imidazolium chloride, followed by treatment with AlCl_3_ and Et_2_AlCl (Scheme 29). A comparative study of the pre-catalysts **Ti2**, bis[N-(salicylideno)anilinato]-titanium(IV) dichloride (**Ti19**), and *N*,*N*′-ethylenebis[5-chloro-salicylideneiminato]titanium(IV) dichloride (**Ti20**) showed that titanocene dichloride **Ti2** catalyzed slurry polymerization of ethylene (30 °C, 5 bar, *n*-hexane) with a higher efficiency (activity up to 7200 kg_PE_∙mol_Ti_^−1^∙h^−1^) in comparison with other pre-catalysts (180 kg_PE_∙mol_Ti_^−1^∙h^−1^ for **Ti19**, 410 kg_PE_∙mol_Ti_^−1^∙h^−1^ for **Ti20**).

**Figure 23:**
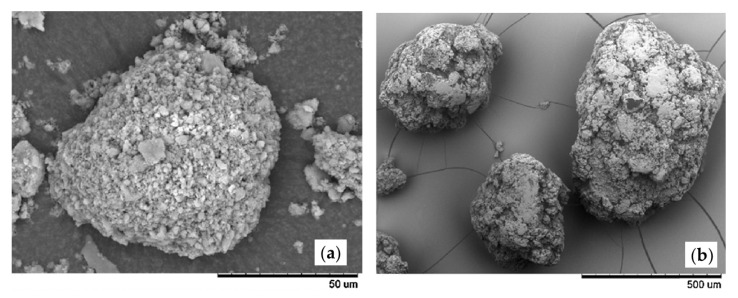
SEM images of the SIL-metallocene catalytic system (**a**) and the obtained polyethylene (**b**). Reprinted with permission from [247]. Copyright (2014) Elsevier B. V.

The **Ti2**/Et_2_AlCl/SIL catalytic system was of spherical morphology (Figure 23a) and provided a formation of PE particles that have the shape of porous granules (Figure 23b). During polymerization experiments using **Ti2**/Et_2_AlCl/SIL, a linear PE was obtained (*T*_m_ = 138–141 °C, degree of crystallinity 62–82%). Therefore, for example, in the experiment, during which the highest catalytic activity was achieved, the resulting PE had the following characteristics: *M*_w_ = 478 kDa, *Ð*_m_ = 3.1, *T*_m_ = 141 °C, and bulk density of 298 g∙dm^−3^.

## 9. Conclusions and Outlook

**Figure 24:**
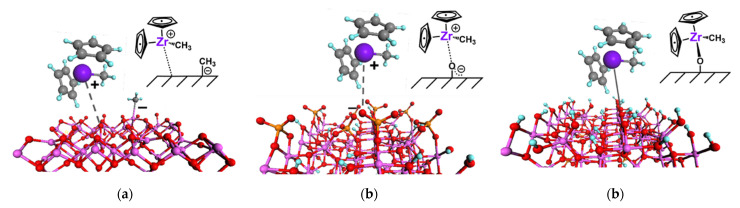
Proposed structures of chemisorbed Cp_2_ZrMe_2_ on the surfaces of dehydroxylated Lewis acidic metal oxides (**a**), highly Brønsted acidic sulfated metal oxides (**b**), and weakly Brønsted acidic hydroxylated metal oxides (**c**). Reprinted with permission from [180]. Copyright (2015) American Chemical Society.

In our review, we summarized scientific periodicals and the patent literature related to the study of the MAO- and borate-free activating supports for Group 4 metallocenes and post-metallocenes and their use in catalytic polymerization of ethylene and α-olefins. In the frameworks of the current views on the mechanism of single-site polymerization catalysis, activation of Group 4 metallocenes and post-metallocenes lies in the formation of alkyl-metal cations, which are capable of α-olefin coordination/insertion. The mechanism of the formation of similar cations depends on the chemical nature of the activating support. If the support contains only strong Lewis acidic sites, activation proceeds via M(IV)→support alkyl migration (MgCl_2_, γ-Al_2_O_3_ calcined at high temperatures). If the support contains mainly Brønsted acidic sites, activation proceeds via protonolysis of M(IV)–alkyl fragment, and catalytic activity depends on the strength of M(IV)–O(support) bonding (sulfated alumina). In the case of weak Brønsted acidic sites such as ≡Si–OH, inactive ≡Si–O–M(IV) species are formed. These three types of processes on the surface activating support are clearly represented in Figure 24 by an example of Cp_2_ZrMe_2_ activation [180].

The surface chemistry of the activation of Group 4 metal complexes was studied by different methods, including MAS NMR, EXAFS, FT-IR spectroscopy, and DFT modeling. Among activating supports, γ-Al_2_O_3_, sulfated alumina, fluorinated alumina, and fluorinated alumina-grafted silica are the most thoroughly studied in terms of their surface structure and activation chemistry. Experimental and theoretical investigations in this field were carried out by scientific groups led by T. Marks, C. Boisson, V. Zakharov, M. Conley, and others. Among well-studied activating supports, sulfated alumina was in the top position in catalytic activity, and values of more than 17 kg∙g_Cat_^−1^∙h^−1^ have been reported [201].

However, recent studies of the chemists of Chevron Phillips led to the development of a new type of activating support, namely, fluorinated silica-grafted alumina, Al_2_O_3_-g-SiO_2_(F). It is noteworthy that the sequential high-temperature chlorination and fluorination of Al_2_O_3_-g-SiO_2_(F,Cl) phases with even higher efficiency. A number of patents describe the use of these phases for the activation of Group 4 metallocenes and hybrid catalytic systems, which contain both single-site and Ziegler–Natta polymerization catalysts. Under optimal conditions and with proper selection of pre-catalysts, activities up to 50 kg∙g_Cat_^−1^∙h^−1^ were achieved. Similar activating supports have not been thoroughly studied with respect to understanding mechanisms of activation and the nature of the active species and counterions. The presence of Cl in most active Al_2_O_3_-g-SiO_2_(F,Cl) supports allows us to draw some analogies with other Group 4 metal catalysts with the obvious, still unclear, but undervalued role of M–(μ-Cl)–Al bonding in α-olefin polymerization [250].

It should also be noted that the total number of Group 4 metallocenes and post-metallocenes, studied in ethylene and α-olefin polymerization with the use of activating supports, is relatively small, with only a few dozen pre-catalysts. That is orders of magnitude less than the total number of pre-catalysts studied with the use of conventional MAO and borate activators. It is safe to assume that further search and design of Group 4 metallocenes and post-metallocenes, which are best suited for the use of activating supports and common alkylaluminums, will allow the creation of efficient heterogeneous catalysts for the production of advanced polyolefins. During this search, model reactions of α-olefin oligomerization [251,252,253] with simple end-group analysis and *DP*_n_ control as well as focusing on metal complexes, capable of homogeneous activation using R_3_Al [254], deserve particular attention.

## Figures and Tables

**Table 1:** Ethylene polymerization using (RCp)_2_ZrCl_2_ and (C_5_H_n_R_5–n_)_2_ZrCl_2_ immobilized on an MgCl_2_-based support [81] ^1^.

Zr Comp.	Cp Ring Substitution	Activity, kg∙mol^−1^∙ bar^−1^∙h^−1^	T_m_, °C	Degree of Crystallinity *χ*_c_	*M*_n_, kDa	*M*_w_, kDa	*Đ*_M_
**Zr2**	H	160	134.5	53.5	88	352	4.0
**Zr6**	Et	260	135.1	55.6	113	394	3.5
**Zr7**	*n*-Pr	2760	135.4	58.5	95	242	2.5
**Zr8**	^i^Pr	360	135.6	51.7	194	485	2.5
**Zr9**	*n*-Bu	3680	135.3	56.9	120	271	2.3
**Zr10**	*^t^*Bu	120	135.1	53.7	126	425	3.4
**Zr11**	*n*-Pentyl	1120	134.5	57.9	98	302	3.1
**Zr12**	*n*-Dodecyl	700	135.6	53.8	122	344	2.8
**Zr13**	1,3-Me_2_	140	135.2	47.1	172	474	2.8
**Zr14**	1-Me-3-*n*-Bu	880	135.9	54.3	217	467	2.2
**Zr15**	1,2,4-Me_3_	120	136.1	50.1	184	430	2.3
**Zr16**	Me_4_	140	139.2	47.3	119	392	3.3
**Zr17**	Me_5_	180	139.2	47.3	148	391	2.6

^1^ Polymerization conditions: 500 mL of light petroleum, 100 mg of immobilized catalyst (1 μmol Zr), 1 mmol of AlEt_3_, 50 °C, ethylene pressure of 5 bar, 1 h.

**Table 2:** Calculated ion pair formation enthalpies ΔH_form_ and heterolytic ion pair separation enthalpies ΔH_ips_ (kcal∙mol^−1^) for activation of Cp_2_ZrMe_2_ on the Al_2_O_3_ (110) surface and with the use of B(C_6_F_5_)_3_ [155].

Complex of Cp_2_ZrMe^+^	*d*(Zr–O), Å ^1^	ΔH_form_, kcal∙mol^−1^	ΔH_ips_, kcal∙mol^−1^
μ^3^-O dioxo-bridged	2.82	−16.7	77.2
μ^3^-O oxo-bridged	2.58	−21.7	82.1
μ^2^-O dioxo-bridged	2.34	−51.7	112.2
μ^2^-O oxo-bridged	2.13	−73.3	127.5
[MeB(C_6_F_5_)_3_^−^] adduct	2.56 ^2^	−1.7	77.0

^1^ In the case of dioxo-bridged structures, a mean distance is given. ^2^ The value of *d*(Zr–C) for Zr–(μ-Me)–B fragment.

**Table 3:** Ethylene polymerization on supported (AlS) and nonsupported constrained-geometry catalysts [197].

Catalyst	T, °C	[M], μmol	Reaction Time, h	Activity, kg∙mol^−1^∙h^−1^	*T*_m_ of PE, °C
**Zr29′**/AlS	25	4.46	3	1.21	138
**Zr30′**/AlS	25	4.02	3	3.51	138
**Zr30′**/AlS	60	2.85	3	4.93	137
**Zr31′**/AlS	25	5.60	3	1.66	139
**Zr31′**/AlS	60	4.46	3	1.19	137
**Ti18′**/AlS	25	2.36	3	14.4	136
**Ti18′**/AlS	60	2.19	3	20.6	137
**Zr30′**/BA ^1^	25	10.0	0.75	127	n. d. ^2^
**Zr31′**/2 BA	25	5.0	1.25	87	n. d.

^1^ Homogeneous polymerization, BA—[Ph_3_C]^+^[B(C_6_F_5_)_4_]^−^. ^2^ No data, low-MW PE obtained (*M*_n_ = 610 and 760 Da, respectively).

**Table 4:** Ethylene polymerization activities of Zr complexes, supported on sulfated Al_2_O_3_ (30 bar, isobutane) [201].

Zr Complex	T, °C	Time, min	AlS Weight, mg	Zr Comp. Weight, mg	R_3_Al/ mmol	PE, g	Activity, kg∙mmol^−1^∙h^−1^	*M*_n_, kDa	*M*_w_, kDa
**Hf6**	90	60	100	3.0	^i^Bu_3_Al/0.5	294	80.8	296	911
**Hf6**	105	35	100	3.0	^i^Bu_3_Al/0.2	203	95.6	239	730
**Hf7**	90	60	100	3.0	^i^Bu_3_Al/0.5	252	68.1	315	972
**Hf7**	105	33	100	3.0	^i^Bu_3_Al/0.2	186	91.3	318	843
**Zr32**	80	30	100	1.0	^i^Bu_3_Al/0.25	315	455.5	305	754
**Zr32**	90	30	105	1.0	^i^Bu_3_Al/0.5	295	426.5	263	639
**Zr32**	90	30	104	1.0	Bu_3_Al/0.5	320	462.7	278	708
**Zr33**	90	30	101	1.0	^i^Bu_3_Al/0.5	272	400.9	223	591
**Zr33**	90	30	108	1.0	Bu_3_Al/0.5	211	311.0	314	750
**Zr34**	90	60	50	2.0	Bu_3_Al/0.5	158	60.4	311	772
**Zr34**	90	30	100	2.0	^i^Bu_3_Al/0.25	255	195.1	205	637
**Zr35**	90	60	100	2.0	^i^Bu_3_Al/0.25	42	14.0	ins.	
**Zr35**	105	60	100	2.0	^i^Bu_3_Al/0.25	63	21.1	ins.	
**Zr36**	90	16	50	1.0	^i^Bu_3_Al/0.5	232	655.2	70	183
**Hf8**	90	60	100	3.0	^i^Bu_3_Al/0.5	294	82.4	108	375
**Hf8**	100	60	100	3.0	^i^Bu_3_Al/0.5	369	103.4	97	267

**Table 5:** Ethylene polymerization activities of Zr complexes, supported on sulfated metal oxides [87].

Support	Zr Complex	[Zr], mol_Zr_/mg	Time, min	Activity, kg∙mol^−1^∙h^−1^	*T*_m_ of PE, °C	Active Site % ^1^
ZrS	**Zr25′**	1.43 × 10^−7^	2	770	133.1	~65
ZrS	Zr(CH_2_Ph)_4_	1.37 × 10^−7^	0.75	2500	134.0	
AlS	**Zr25′**	3.9 × 10^−8^	10	1100	133.6	87 ± 3
AlS	Zr(CH_2_Ph)_4_	3.9 × 10^−8^	8	2100	134.2	
SnS	**Zr25′**	4.4 × 10^−8^	60	98	136.1	61 ± 5
SnS	Zr(CH_2_Ph)_4_	4.4 × 10^−8^	30	660	135.1	
FeS	**Zr25′**	9.0 × 10^−8^	30	24	134.2	22 ± 2
FeS	Zr(CH_2_Ph)_4_	9.0 × 10^−8^	7	510	135.2	
TiS	**Zr25′**	1.85 × 10^−7^	120	1.2	138.4	63 ± 9
TiS	Zr(CH_2_Ph)_4_	1.85 × 10^−7^	60	63	138.0	

^1^ Determined by poisoning **Zr25′**/SMO ethylene polymerization with neopentyl alcohol.

**Table 6:** Characteristics of the supports and ethylene polymerization results [214].

Support	*C*_S_ of Type L1 and L2 LAS, μmol∙g^−1^	*C*_S_ of Type L3 LAS, μmol∙g^−1^	*C*_S_ of BAS, μmol∙g^−1^	*PA*, kJ∙mol^−1^	Zr2–Based Catalyst			Zr2′–Based Catalyst		
					Zr wt% (μmol∙g^−1^)	PE Yield, g	Activity, kg∙mol^−1^∙h^−1^	Zr wt% (μmol∙g^−1^)	PE Yield, g	Activity, kg∙mol^−1^∙h^−1^
γ-Al_2_O_3_	60, 660	–	35	1380	1.21 (133)	3.3	25	4.07 (447)	4.5	9
Al_2_O_3_(1%F)	380	20	4	1280	0.62 (68)	11	162	1.93 (212)	6.4	30
Al_2_O_3_(3%F)	180	50	2	1190	0.60 (66)	20	303	2.53 (278)	46	166
Al_2_O_3_(5%F)	120	50	7.3	1190	0.62 (68)	61	895	2.53 (278)	149.5	538
Al_2_O_3_(7%F)	100	50	6.1	1190	0.66 (73)	30	414	2.53 (278)	51	183

**Table 7:** The types and characteristics of SiO_2_; reagents and conditions of the preparation of SiO_2_/g-Al_2_O_3_(F) activating supports and their catalytic applications.

Silica/Specific Surface Area, m^2^∙g^−1^ / Pore Volume, mL∙g^−1^/ Particle Size, μm	Organo- Aluminum Compound	Stages 2, 3; Calcination Regime	Stage 3: Fluorinating Reagent/3rd Calcination Regime	Pre-catalyst/wt% or (mmol∙g^−1^)	R_3_Al	Polymer/ Activity, g∙g_Cat_^−1^∙h^−1^ (kg∙mol^−1^∙h^−1^)	Ref.
Grace 332/300/1.65/70	(BuO)_2_AlOSi (OEt)_3_	20→130 °C, 1 h 130→450 °C, 1 h 450 °C, 1 h	6 wt% (NH_4_)_2_SiF_6_ / 20→130 °C, 1 h 130→450 °C, 1 h 450 °C, 1 h	**Zr2**/(53)	^i^Bu_3_Al	PE/—(900)	[88]
				**Zr2**/(53)	Et_3_Al	PE/—(490)	[88]
				**Zr37**/(53)	^i^Bu_3_Al	PE/—(6720)	[88]
				**Zr38**/(53)	^i^Bu_3_Al	PE/—(2020)	[88] ^1^
Grace 332/300/1.65/70	Et_3_Al	20→130 °C, 1 h 130 °C, 1 h 130→450 °C, 1 h 450 °C, 4 h	5 wt% (NH_4_)_2_SiF_6_/ 30→450 °C, 2 h 450 °C, 2 h	**Zr5**/n. d.	^i^Bu_3_Al	PE/39 (3400)	[107]
Grace 332/300/1.65/70			10 wt% (NH_4_)_2_SiF_6_/ 30→450 °C, 2 h 450 °C, 2 h	**Zr5**/n. d.	^i^Bu_3_Al	ethylene—hex-1-ene (20 mol%)/550 (75,000)	[107]
Crossfield ES70X/276/1.54/53				**Zr5**/n. d.	^i^Bu_3_Al	ethylene—hex-1-ene (20 mol%)/725 (91,000)	[107]
Grace 332/300/1.65/70	Et_2_AlF	20→130 °C, 1 h 130 °C, 1 h 130→450 °C, 1 h 450 °C, 1 h	–	**Zr38**	^i^Bu_3_Al	PE/820 (–)	[102]
Grace 332/300/1.65/70				**Zr39**/1.0	^i^Bu_3_Al	PP/700 (–) ^2^	[102,107]
Grace 332/300/1.65/70				**Zr5**/	^i^Bu_3_Al	ethylene—hex-1-ene (20 mol%)/110 (19,000)	[107]
MS-3030/320/3/85				**Zr5**/0.4	^i^Bu_3_Al	PE/160 (–)	[105]
MS-3040/420/3/80				**Zr5**/0.4	^i^Bu_3_Al	PE/320 (–)	–”–
SIL 3/800/1/15				**Zr5**/0.4	^i^Bu_3_Al	PE/0 (–)	–”–
SIL 19/700/2.2/15				**Zr5**/0.4	^i^Bu_3_Al	PE/280 (–)	–”–
SIL 20/630/3.2/100				**Zr5**/0.4	^i^Bu_3_Al	PE/400 (–)	–”–
SIL 30/300/3/100				**Zr5**/0.4	^i^Bu_3_Al	PE/250 (–)	–”–
SP9 446/520/1.9/53				**Zr5**/0.4	^i^Bu_3_Al	PE/1080 (–)	–”–
Grace 948/290/1.7/58				**Zr5**/0.4	^i^Bu_3_Al	PE/700 (–)	–”–

^1^ In [88], ethylene/hex-1-ene copolymerization results for **Zr5**/^i^Bu_3_Al/AS are also mentioned without meaningful results. ^2^ The percentage of isotactic pentads *mmmm* = 96%.

**Table 8:** Preparation and elemental analysis of activating supports [107].

Support	Silica ^1^	Alkylaluminum	(NH_4_)_2_SiF_6_ wt%	Al wt%	F wt%
AS1	Grace 332	Et_3_Al	5	3.72	1.18
AS2	Grace 332	Et_3_Al	5	4.75	1.74
AS3	Grace 332	Et_3_Al	10	3.67	3.31
AS4	Grace 332	Et_3_Al	20	3.81	5.30
AS5	Grace 332	Et_2_AlF	–	4.56	2.21
AS6	Grace 332	Et_3_Al	10	4.98	3.60
AS7	ES70X	Et_3_Al	10	3.01	3.98
AS8 ^2^	ES70X	Et_3_Al	10	3.20	2.20
AS9 ^3^	ES70X	Et_3_Al	5	3.26	2.34

^1^ Properties of silicas are given in Table 2. Before treatment with alkylaluminum, silicas were calcined at 0.01 mbar and 450 °C, [Si–OH] = 1.3 mmol∙g^−1^. ^2^ Pre-calcining temperature was 200 °C. ^3^ Pre-calcining temperature was 300 °C.

**Table 9:** Activation of a range of Group 4 metal complexes using AS6, AS7, and MAO [107] ^1^.

Comp	Activator	Hex-1-ene mol%	Activity, kg∙mmol^−1^∙h^−1^	Activity, g∙g_Cat_^−1^∙h^−1^	*M*_w_, kDa	*Đ*_M_	*T*_m_, °C
**Zr5**	AS6 (20 mg)	20	75	550	97	3.7	111
**Zr5**	AS7 (20 mg)	20	91	725	125	3.6	112
**Zr9**	MAO (2000 eq)	0	150		206	2.9	134
**Zr9**	MAO (2000 eq)	20	76		160	3.0	134
**Zr9**	AS6 (21 mg)	20	9.1	71	102	3.0	124
**Zr9**	AS7 (23 mg)	0	28	142	146	2.3	131
**Zr9**	AS7 (21 mg)	20	22	462	94	3.1	119
**Zr26** ^2^	MAO (2000 eq)	0	140		704	5.1	140
**Zr26** ^2^	MAO (2000 eq)	20	27		154	2.1	90
**Zr26** ^2^	AS6 (20 mg)	20	24	196	346	5.2	81
**Zr26** ^3^	AS7 (43 mg)	0	4.2	29	557	3.6	131
**Zr26** ^2^	AS7 (26 mg)	20	32	189	193	3.3	84
**Zr3**	MAO (2000 eq)	0	2.6		161	3.7	131
**Zr3** ^2^	MAO (2000 eq)	20	9.5		51	2.0	121
**Zr3** ^2^	AS6 (48 mg)	20	33	224	144	4.0	116
**Zr3**	AS7 (41 mg)	0	2.9	23	342	4.1	135
**Zr3** ^2^	AS7 (42 mg)	20	43	338	122	3.5	111
**Ti8**	MAO (2000 eq)	0	1.1		173	3.3	138
**Ti8** ^2^	MAO (2000 eq)	20	3.9		15	1.9	100
**Ti8** ^2^	AS6 (108 mg)	20	38	287	186	3.3	103
**Ti8**	AS7 (112 mg)	0	3.3	24	1022	5.7	133
**Ti8** ^2^	AS7 (112 mg)	20	12	95	356	5.5	101

^1^ 300 mL heptane, 4 bar ethylene, 80 °C, 60 min, MAO 10 wt %. For **Zr5**, **Zr9**, and **Zr26**, [Zr] = 0.5 μmol, [Al] = 1 mmol; **Zr3** [Zr] = 2 μmol, [Al] = 4 mmol; and for **Ti8** [Ti] = 2.5 μmol, [Al] = 5 mmol. ^2^ 15 min. ^3^ [Zr] = 1 μM.

**Table 10:** Characteristics of the silica used for the synthesis of the SiO_2_/g-Al_2_O_3_(F) supports [106].

Silica	Pore Volume, mL∙g^−1^	Specific Surface Area, m^2^∙g^−1^	Particle Size, μm	Mean Pore Diameter, Å
Grace Sylopol 952	1.8	290	58	246
Grace Sylopol 948	1.7	290	58	232
SP9_446	1.9	520	53	131
M3703/079A	1.9	240	53	240

**Table 11:** Catalytic activities of zirconocenes **Zr5** and **Zr40** on different activating supports in ethylene/hex-1-ene copolymerization and the values of tan δ for the copolymers obtained [94].

Activating Support (Al_2_O_3_/SiO_2_ by Weight)	Zr5		Zr40	
	Activity, kg∙g_Cat_^−1^∙h^−1^	Tan δ	Activity, kg∙g_Cat_^−1^∙h^−1^	Tan δ
Sulfated Al_2_O_3_ (–)	0.18	2.89	1.27	14.43
Fluorinated Al_2_O_3_ (–)	0.02	1.34	3.11	4.12
Chlorinated Al_2_O_3_ (–)	0.07	1.88	0.36	1.53
SiO_2_/Al_2_O_3_ (0.15:1)	0.15	1.17	1.61	7.05
Al_2_O_3_/g-SiO_2_(F) (2.6:1)	9.10	5.78	6.35	5.44
Sulfated Al_2_O_3_/g-SiO_2_(F) (2.6:1) ^1^	6.69	3.69	4.94	9.99
Phosphated Al_2_O_3_/g-SiO_2_(F) (P) ^2^	6.41	5.00	5.13	8.79

^1^ Al_2_O_3_/g-SiO_2_ was treated by MeOH solution of NH_4_HF_2_ and H_2_SO_4_ with subsequent calcining at 600 °C for 3 h. ^2^ H_3_PO_4_ was used instead of H_2_SO_4_.

**Table 12:** Oligomerization of 1-octene, catalyzed by zirconium complexes, and activated by TIBA and fluorinated aluminosilicate [97].

Cat.	[Mon]/[Zr] Ratio	T, °C	TOF, h^−1^	*KV* ^100^	*VI*
**Zr2**	1.1·10^5^	90	3.1·10^3^	8.9	211
**Zr5**	4.2·10^5^	115	4.3·10^4^	136	210
**Zr6**	1.5·10^6^	70	1.3·10^5^	130	222
**Zr8**	3.1·10^5^	110	3.4·10^4^	62	186
**Zr9**	5.6·10^5^	105	4.1·10^4^	45	175
**Zr35**	1.1·10^5^	90	9.0·10^3^	23	169
**Zr43**	4.3·10^5^	120	3.1·10^4^	159	214
**Zr44**	4.4·10^5^	120	3.6·10^4^	132	200
**Zr45**	1.0·10^5^	100	5.2·10^3^	10.3	194
**Hf10**	5.5·10^5^	90	2.5·10^4^	8.3	157

**Table 13:** Ethylene (co)-polymerization results from the combination of AS-1 and sMAO solid activators with zirconium metallocene complexes **Zr5** and **Zr9** (80 °C, 4 bar, [^i^Bu_3_Al] = 1 mmol, heptane, 30 min) [54].

Catalyst	Mol% hex-1-ene	Al_surface_/ Zr Ratio	Wt% ^1^	[Zr], μmol	Activity, g∙g_Cat_^−1^∙h^−1^	Activity, kg∙mol_Zr_^−1^∙h^−1^
**Zr5**/AS-1	–	9.6	1.18	2.0	250	8800
**Zr5**/AS-1	19.5	9.3	1.22	2.05	656	22,500
**Zr9**/AS-1	–	9.0	1.21	2.0	130	4300
**Zr9**/AS-1	19.5	9.0	1.21	2.0	133	4400
**Zr5**/sMAO	–	120	1.56	2.1	200	5370
**Zr5**/sMAO	19.5	120	1.56	2.1	620	16,600
**Zr9**/sMAO	–	148	1.32	2.0	326	11,400
**Zr9**/sMAO	19.5	148	1.55	2.0	230	8600

^1^ Weight percent of metallocene in the supported metallocene catalysts.

**Table 14:** Influence of the kind of the ionic liquid (IL) and the [AlEtCl_2_]/[**Ti2**] molar ratio on PE yield in the biphasic IL/hexane polymerization of ethylene (30 °C, 5 bar).

IL	[AlEtCl_2_]/ [Ti2] Ratio	Reaction Time, h	PE Yield, kg_PE_∙mol_Ti_^−1^		
			IL Phase	Hexane Phase	Total
[C_8_-mim]^+^ [AlCl_4_]^−^	67	1	10.7	46.7	57.3
	100	1	14.4	62.0	76.4
	133	1	17.0	101.0	118.0
	167	1	78.0	85.7	163.7
	100	2	13.7	146.7	160.3
	133	2	48.3	147.0	195.3
[C_8_-β-mpy]^+^ [AlCl_4_]^−^	67	1	8.0	68.0	76.0
	100	1	76.7	36.7	113.3
	133	1	101.7	41.0	142.7
	67	2	65.7	37.6	103.3
	133	2	103.3	105.0	208.3
[C_8_-γ-mpy]^+^ [AlCl_4_]^−^	67	1	5.3	59.8	65.1
	100	1	94.3	42.0	136.3
	133	1	171.5	37.5	209.0
	67	2	8.9	81.1	90.1
	133	2	42.0	236.7	278.7

## Data Availability

Not applicable.

## References

[1] Jubinville D., Esmizadeh E., Saikrishnan S., Tzoganakis C., Mekonnen T. (2020). A comprehensive review of global production and recycling methods of polyolefin (PO) based products and their post-recycling applications. Sustain. Mater. Technol..

[2] Qiao J., Guo M., Wang L., Liu D., Zhang X., Yu L., Song W., Liu Y. (2011). Recent advances in polyolefin technology. Polym. Chem..

[3] Chum P.S., Swogger K.W. (2008). Olefin polymer technologies—History and recent progress at The Dow Chemical Company. Prog. Polym. Sci..

[4] Sauter D.W., Taoufik M., Boisson C. (2017). Polyolefins, a Success Story. Polymers.

[5] Geyer R., Jambeck J.R., Law K.L. (2017). Production, use, and fate of all plastics ever made. Sci. Adv..

[6] McDaniel M.P. (2010). Chapter 3—A Review of the Phillips Supported Chromium Catalyst and Its Commercial Use for Ethylene Polymerization. Adv. Catal..

[7] Shamiri A., Chakrabarti M.H., Jahan S., Hussain M.A., Kaminsky W., Aravind P.V., Yehye W.A. (2014). The Influence of Ziegler-Natta and Metallocene Catalysts on Polyolefin Structure, Properties, and Processing Ability. Materials.

[8] Chen C. (2018). Designing catalysts for olefin polymerization and copolymerization: Beyond electronic and steric tuning. Nat. Rev. Chem..

[9] Chen E.Y.-X., Marks T.J. (2000). Cocatalysts for Metal-Catalyzed Olefin Polymerization: Activators, Activation Processes, and Structure-Activity Relationships. Chem. Rev..

[10] Sinn H., Kaminsky W., Vollmer H.J., Woldt R. (1980). “Living Polymers” on Polymerization with Extremely Productive Ziegler Catalysts. Angew. Chem. Int. Ed..

[11] Bochmann M. (2010). The Chemistry of Catalyst Activation: The Case of Group 4 Polymerization Catalysts. Organometallics.

[12] Yang X., Stern C.L., Marks T.J. (1992). Cationic Metallocene Polymerization Catalysts. Synthesis and Properities of the Cationic Metallocene Polymerization Catalysts. Synthesis and Properities of the First Base-Free Zirconocene Hydride. Angew. Chem. Int. Ed..

[13] Parveen R., Cundari T.R., Younker J.M., Rodriguez G. (2020). Computational Assessment of Counterion Effect of Borate Anions on Ethylene Polymerization by Zirconocene and Hafnocene Catalysts. Organometallics.

[14] Faingol’d E.E., Bravaya N.M., Panin A.N., Babkina O.N., Saratovskikh S.L., Privalov V.I. (2016). Isobutylaluminum aryloxides as metallocene activators in Homo- and copolymerization of olefins. J. Appl. Polym. Sci..

[15] Tritto I., Zucchi D., Destro M., Sacchi M.C., Dall’Occo T., Galimberti M. (1990). NMR investigations of the reactivity between zirconocenes and β-alkyl-substituted aluminoxanes. J. Mol. Catal. A Chem..

[16] Bravaya N.M., Panin A.N., Faingol’d E.E., Saratovskikh S.L., Babkina O.N., Zharkov I.V., Perepelitsina E.O. (2016). Isobutylalumoxanes as high-performance activators of *rac*-Et(2-MeInd)_2_ZrMe_2_ in copolymerization of ethylene with propylene and ternary copolymerization of ethylene, propylene, and 5-ethylidene-2-norbornene. Polym. Bull..

[17] Tritto I., Boggioni L., Sacchi M.C., Dall’Occo T. (2003). Novel aluminum based cocatalysts for metallocene catalyzed olefin polymerization. J. Mol. Catal. A Chem..

[18] Kaminsky W. (2012). Discovery of Methylaluminoxane as Cocatalyst for Olefin Polymerization. Macromolecules.

[19] Babushkin D.E., Semikolenova N.V., Zakharov V.A., Talsi E.P. (2000). Mechanism of dimethylzirconocene activation with methylaluminoxane: NMR monitoring of intermediates at high Al/Zr ratios. Macromol. Chem. Phys..

[20] Theurkauff G., Bader M., Marquet N., Bondon A., Roisnel T., Guegan J.-P., Amar A., Boucekkine A., Carpentier J.-F., Kirillov E. (2016). Discrete Ionic Complexes of Highly Isoselective Zirconocenes. Solution Dynamics, Trimethylaluminum Adducts, and Implications in Propylene Polymerization. Organometallics.

[21] Joshi A., Zijlstra H.S., Collins S., McIndoe J.S. (2020). Catalyst Deactivation Processes during 1-Hexene Polymerization. ACS Catal..

[22] Parfenova L.V., Khalilov L.M., Dzhemilev U.M. (2012). Mechanisms of reactions of organoaluminium compounds with alkenes and alkynes catalyzed by Zr complexes. Russ. Chem. Rev..

[23] Baldwin S.M., Bercaw J.E., Brintzinger H.H. (2008). Alkylaluminum-Complexed Zirconocene Hydrides: Identification of Hydride-Bridged Species by NMR Spectroscopy. J. Am. Chem. Soc..

[24] Parfenova L.V., Kovyazin P.V., Nifant’ev I.E., Khalilov L.M., Dzhemilev U.M. (2015). Role of Zr, Al Hydride Intermediate Structure and Dynamics in Alkene Hydroalumination with XAlBu^i^_2_ (X = H, Cl, Bu^i^), Catalyzed by Zr η^5^ Complexes. Organometallics.

[25] Parfenova L.V., Kovyazin P.V., Bikmeeva A.K., Palatov E.R. (2021). Catalytic Systems Based on Cp_2_ZrX_2_ (X = Cl, H), Organoaluminum Compounds and Perfluorophenylboranes: Role of Zr,Zr- and Zr,Al-Hydride Intermediates in Alkene Dimerization and Oligomerization. Catalysts.

[26] Baldwin S.M., Bercaw J.E., Henling L.M., Day M.W., Brintzinger H.H. (2011). Cationic Alkylaluminum-Complexed Zirconocene Hydrides: NMR-Spectroscopic Identification, Crystallographic Structure Determination, and Interconversion with Other Zirconocene Cations. J. Am. Chem. Soc..

[27] Baldwin S.M., Bercaw J.E., Brintzinger H.H. (2010). Cationic Alkylaluminum-Complexed Zirconocene Hydrides as Participants in Olefin Polymerization Catalysis. J. Am. Chem. Soc..

[28] Alt H.G., Denner C.E., Thewalt U., Rausch M.D. (1988). Cp_2_Zr(C_2_H_4_)(PMe_3_), der erste stabile Ethylenkomplex des Zirkoniums. J. Organomet. Chem..

[29] Tamotsu T., Masato M., Masaki K., Masahiko S., Yasuzo U., Kozo K., Tokiko U., Swanson D.R., Negishi E. (1989). Zirconocene–Alkene Complexes. An X-Ray Structure and a Novel Preparative Method. Chem. Lett..

[30] Bochmann M., Sarsfield M.J. (1998). Reaction of AlR_3_ with [CPh_3_][B(C_6_F_5_)_4_]:  Facile Degradation of [B(C_6_F_5_)_4_]^−^ by Transient “[AlR_2_]^+^”. Organometallics.

[31] Bolton P.D., Adams N., Clot E., Cowley A.R., Wilson P.J., Schroder M., Mountford P. (2006). Synthesis and Ethylene Polymerization Capability of Metallocene-like Imido Titanium Dialkyl Compounds and Their Reactions with Al^i^Bu_3_. Organometallics.

[32] Götz C., Rau A., Luft G. (2002). Ternary metallocene catalyst systems based on metallocene dichlorides and AlBu_3_^i^/[PhNMe_2_H][B(C_6_F_5_)_4_]: NMR investigations of the influence of Al/Zr ratios on alkylation and on formation of the precursor of the active metallocene species. J. Mol. Catal. A Chem..

[33] Zaccaria F., Zuccaccia C., Cipullo R., Budzelaar P.H.M., Vittoria A., Macchioni A., Busico V., Ehm C. (2021). Methylaluminoxane’s Molecular Cousin: A Well-defined and “Complete” Al-Activator for Molecular Olefin Polymerization Catalysts. ACS Catal..

[34] Parfenova L.V., Balaev A.V., Gubaidullin I.M., Abzalilova L.R., Pechatkina S.V., Khalilov L.M., Spivak S.I., Dzhemilev U.M. (2007). Kinetic model of olefin hydroalumination by HAlBu^i^_2_ and AlBu^i^_3_ in the presence of Cp_2_ZrCl_2_ catalyst. Int. J. Chem. Kinet..

[35] Parfenova L.V., Pechatkina S.V., Khalilov L.M., Dzhemilev U.M. (2005). Mechanism of Cp_2_ZrCl_2_-catalyzed olefin hydroalumination by alkylalanes. Russ. Chem. Bull..

[36] Culver D.B., Corieri J., Lief G., Conley M.P. (2022). Reactions of Triisobutylaluminum with Unbridged or Bridged Group IV Metallocene Dichlorides. Organometallics.

[37] Negishi E., Kondakov D.Y., Choueiry D., Kasai K., Takahashi T. (1996). Multiple Mechanistic Pathways for Zirconium-Catalyzed Carboalumination of Alkynes. Requirements for Cyclic Carbometalation Processes Involving C-H Activation. J. Am. Chem. Soc..

[38] Khalilov L.M., Parfenova L.V., Rusakov S.V., Ibragimov A.G., Dzhemilev U.M. (2000). Synthesis and conversions of metallocycles. 22. NMR studies of the mechanism of Cp_2_ZrCl_2_-catalyzed cycloalumination of olefins with triethylaluminium to form aluminacyclopentanes. Russ. Chem. Bull..

[39] Severn J.R., Chadwick J.C., Duchateau R., Friederichs N. (2005). “Bound but Not Gagged” Immobilizing Single-Site α-Olefin Polymerization Catalysts. Chem. Rev..

[40] Hlatky G.G. (2000). Heterogeneous Single-Site Catalysts for Olefin Polymerization. Chem. Rev..

[41] Copéret C., Allouche F., Chan K.W., Conley M.P., Delley M.F., Fedorov A., Moroz I.B., Mougel V., Pucino M., Searles K. (2018). Bridging the Gap between Industrial and Well-Defined Supported Catalysts. Angew. Chem. Int. Ed..

[42] Copéret C., Comas-Vives A., Conley M.P., Estes D.P., Fedorov A., Mougel V., Nagae H., Núñez-Zarur F., Zhizhko P.A. (2016). Surface Organometallic and Coordination Chemistry toward Single-Site Heterogeneous Catalysts: Strategies, Methods, Structures, and Activities. Chem. Rev..

[43] Korzyński M.D., Copéret C. (2021). Single sites in heterogeneous catalysts: Separating myth from reality. Trends Chem..

[44] Fink G., Steinmetz B., Zechlin J., Przybyla C., Tesche B. (2000). Propene Polymerization with Silica-Supported Metallocene/MAO Catalysts. Chem. Rev..

[45] Klapper M., Joe D., Nietzel S., Krumpfer J.W., Müllen K. (2014). Olefin Polymerization with Supported Catalysts as an Exercise in Nanotechnology. Chem. Mater..

[46] Stalzer M.M., Delferro M., Marks T.J. (2015). Supported Single-Site Organometallic Catalysts for the Synthesis of High-Performance Polyolefins. Catal. Lett..

[47] Werny M.J., Müller D., Hendriksen C., Chan R., Friederichs N.H., Fella C., Meirer F., Weckhuysen B.M. (2022). Elucidating the Sectioning Fragmentation Mechanism in Silica-Supported Olefin Polymerization Catalysts with Laboratory-Based X-Ray and Electron Microscopy. ChemCatChem.

[48] Bashir M.A., Monteil V., Boisson C., McKenna T.F.L. (2017). Avoiding leaching of silica supported metallocenes in slurry phase ethylene homopolymerization. React. Chem. Eng..

[49] Bashir M.A., Vancompernolle T., Gauvin R.M., Delevoye L., Merle N., Monteil V., Taoufik M., McKenna T.F.L., Boisson C. (2016). Silica/MAO/(*n*-BuCp)_2_ZrCl_2_ catalyst: Effect of support dehydroxylation temperature on the grafting of MAO and ethylene polymerization. Catal. Sci. Technol..

[50] Velthoen M.E.Z., Boereboom J.M., Bulo R.E., Weckhuysen B.M. (2019). Insights into the activation of silica-supported metallocene olefin polymerization catalysts by methylaluminoxane. Catal. Today.

[51] Jeong S.M., Park J.Y., Hyun Y.B., Baek J.W., Kim H., Yoon Y., Chung S., Lee J., Lee B.Y. (2022). Syntheses of Silylene-Bridged Thiophene-Fused Cyclopentadienyl *ansa*-Metallocene Complexes for Preparing High-Performance Supported Catalyst. Catalysts.

[52] Charoenchaidet S., Chavadej S., Gulari E. (2002). Borane-functionalized silica supports: In situ activated heterogeneous zirconocene catalysts for MAO-free ethylene polymerization. J. Mol. Catal. A Chem..

[53] Lamb J.V., Buffet J.-C., Turner Z.R., O’Hare D. (2020). Ethylene Polymerization Using Zirconocenes Supported on Pentafluorophenyl-Modified Solid Polymethylaluminoxane. Macromolecules.

[54] Sauter D.W., Popoff N., Bashir M.A., Szeto K.C., Gauvin R.M., Delevoye L., Taoufik M., Boisson C. (2016). The design of a bipodal bis(pentafluorophenoxy)aluminate supported on silica as an activator for ethylene polymerization using surface organometallic chemistry. Chem. Commun..

[55] Iiskola E.I., Timonen S., Pakkanen T.T., Harkki O., Lehmus P., Seppala J.V. (1997). Cyclopentadienyl Surface as a Support for Zirconium Polyethylene Catalysts. Macromolecules.

[56] Leadbeater N.E., Marco M. (2002). Preparation of Polymer-Supported Ligands and Metal Complexes for Use in Catalysis. Chem. Rev..

[57] Zhang J., Jin C.-X. (2006). Self-immobilized and polymerized miscellaneous metallocene catalysts for ethylene polymerization. Inorg. Chem. Commun..

[58] McKittrick M.W., Jones C.W. (2004). Toward Single-Site, Immobilized Molecular Catalysts: Site-Isolated Ti Ethylene Polymerization Catalysts Supported on Porous Silica. J. Am. Chem. Soc..

[59] Schneider H., Puchta G.T., Kaul F.A.R., Raudaschl-Sieber G., Lefebvre F., Saggio G., Mihalios D., Herrmann W.A., Basset J.M. (2001). Immobilization of η^5^-cyclopentadienyltris(dimethylamido)zirconium polymerization catalysts on a chlorosilane- and HMDS-modified mesoporous silica surface: A new concept for supporting metallocene amides towards heterogeneous single-site-catalysts. J. Mol. Catal. A Chem..

[60] Witzke R.J., Chapovetsky A., Conley M.P., Kaphan D.M., Delferro M. (2020). Nontraditional Catalyst Supports in Surface Organometallic Chemistry. ACS Catal..

[61] Ballard D.G.H. (1975). Transition metal alkyl compounds as polymerization catalysts. J. Polym. Sci. Polym. Chem. Ed..

[62] Dudchenko V.K., Zakharov V.A., Maksimov N.G., Yermakov Y.I. (1977). Comparison of properties of supported organozirconium catalysts containing zirconium ions in different valence states. React. Kinet. Catal. Lett..

[63] Zakharov V.A., Yermakov Y.I. (1979). Supported Organometallic Catalysts for Olefin Polymerization. Catal. Rev. Sci. Eng..

[64] Martineau D., Dumas P., Sigwalt P. (1983). Role of cocatalysts in polymerizations initiated with benzyl derivatives of transition metals, 1. Polymerization of ethylene. Makromol. Chem..

[65] Ittel S.D. (1990). Elastomeric Polypropylene Using Alumina-Supported Bis(Arene)Titanium and Tetra(Neophyl)Zirconium Catalysts. J. Macromol. Sci. A Chem..

[66] Ballard D.G.H., Jones E., Padget J.C., Pioli A.J.P., Robinson P.A., Walker J., Wyatt R.J. (1977). Polymerization Process. U.S. Patent.

[67] Popoff N., Espinas J., Pelletier J., Macqueron B., Szeto K.C., Boyron O., Boisson C., Del Rosal I., Maron L., De Mallmann A. (2013). Small Changes Have Consequences: Lessons from Tetrabenzyltitanium and -zirconium Surface Organometallic Chemistry. Chem. Eur. J..

[68] Tullock C.W., Mulhaupt R., Ittel S.D. (1989). Elastomeric polypropylene (ELPP) from alumina-supported bis(mesitylene)titanium catalysts. Makromol. Chem. Rapid Commun..

[69] Tullock C.W., Tebbe F.N., Mulhaupt R., Ovenall D.W., Setterquist R.A., Ittel S.D. (1989). Polyethylene and elastomeric polypropylene using alumina-supported bis(arene) titanium, zirconium, and hafnium catalysts. J. Polym. Sci. A Polym. Chem..

[70] Toscano P.J., Marks T.J. (1985). Supported organoactinides. High-resolution solid-state carbon-13 NMR studies of catalytically active, alumina-bound pentamethylcyclopentadienyl)thorium methyl and hydride complexes. J. Am. Chem. Soc..

[71] Dahmen K.H., Hedden D., Burwell R.L., Marks T.J. (1988). Organometallic molecule-support interactions. Highly active organozirconium hydrogenation catalysts and the formation of cationic species on alumina surfaces. Langmuir.

[72] Marks T.J. (1992). Surface-bound metal hydrocarbyls. Organometallic connections between heterogeneous and homogeneous catalysis. Acc. Chem. Res..

[73] Kaminaka M., Soga K. (1991). Polymerization of propene with the catalyst systems composed of Al_2_O_3_- or MgCl_2_-supported Et[IndH_4_]_2_ZrCl_2_ and AlR_3_ (R = CH_3_, C_2_H_5_). Makromol. Chem. Rapid Comm..

[74] Kaminaka M., Soga K. (1992). Polymerization of propene with catalyst systems composed of Al_2_O_3_ or MgCl_2_-supported zirconocene and Al(CH_3_)_3_. Polymer.

[75] Soga K., Kaminaka M. (1993). Polymerization of propene with zirconocene-containing supported catalysts activated by common trialkylaluminiums. Makromol. Chem..

[76] Kaji E., Uozumi T., Jin J., Sano T., Soga K. (1998). Ethene–propene copolymerization with the MgCl_2_-supported dichlorobis(4,4,4-trifluoro-1-phenyl-1,3-butanedionato)titanium catalyst activated by an ordinary trialkylaluminum. J. Polym. Sci. A Polym. Chem..

[77] Nakayama Y., Bando H., Kaneko H., Sonobe Y., Mitani M., Kojoh S., Kashiwa N., Fujita T. (2003). MAO-Free new olefin polymerization catalyst systems based on FI catalysts. Stud. Surf. Sci. Catal..

[78] Nakayama Y., Bando H., Sonobe Y., Kaneko H., Kashiwa N., Fujita T. (2003). New olefin polymerization catalyst systems comprised of bis(phenoxy-imine) titanium complexes and MgCl_2_-based activators. J. Catal..

[79] Nakayama Y., Bando H., Sonobe Y., Fujita T. (2004). Development of Single-Site New Olefin Polymerization Catalyst Systems Using MgCl_2_-Based Activators: MAO-Free MgCl_2_-Supported FI Catalyst Systems. Bull. Chem. Soc. Jpn..

[80] Severn J.R., Chadwick J.C. (2004). MAO-Free Activation of Metallocenes and other Single-Site Catalysts for Ethylene Polymerization using Spherical Supports based on MgCl_2_. Macromol. Rapid. Commun..

[81] Huang R., Duchateau R., Koning C.E., Chadwick J.C. (2008). Zirconocene Immobilization and Activation on MgCl_2_-Based Supports: Factors Affecting Ethylene Polymerization Activity. Macromolecules.

[82] Jezequel M., Dufaud V., Ruiz-Garcia M.J., Carrillo-Hermosilla F., Neugebauer U., Niccolai G.P., Lefebvre F., Bayard F., Corker J., Fiddy S. (2001). Supported Metallocene Catalysts by Surface Organometallic Chemistry. Synthesis, Characterization, and Reactivity in Ethylene Polymerization of Oxide-Supported Mono- and Biscyclopentadienyl Zirconium Alkyl Complexes: Establishment of Structure/Reactivity Relationships. J. Am. Chem. Soc..

[83] Ahn H., Marks T.J. (1998). Supported Organometallics. Highly Electrophilic Cationic Metallocene Hydrogenation and Polymerization Catalysts Formed via Protonolytic Chemisorption on Sulfated Zirconia. J. Am. Chem. Soc..

[84] Ahn H., Nicholas C.P., Marks T.J. (2002). Surface Organozirconium Electrophiles Activated by Chemisorption on “Super Acidic” Sulfated Zirconia as Hydrogenation and Polymerization Catalysts. A Synthetic, Structural, and Mechanistic Catalytic Study. Organometallics.

[85] Nicholas C.P., Ahn H., Marks T.J. (2003). Synthesis, Spectroscopy, and Catalytic Properties of Cationic Organozirconium Adsorbates on “Super Acidic” Sulfated Alumina. “Single-Site” Heterogeneous Catalysts with Virtually 100 Active Sites. J. Am. Chem. Soc..

[86] Nicholas C.P., Marks T.J. (2004). Sulfated Tin Oxide Nanoparticles as Supports for Molecule-Based Olefin Polymerization Catalysts. Nano Lett..

[87] Nicholas C.P., Marks T.J. (2004). Zirconium Hydrocarbyl Chemisorption on Sulfated Metal Oxides: New Supports, Chemisorption Pathways, and Implications for Catalysis. Langmuir.

[88] Saudemint T., Spitz R., Broyer J.-P., Malinge J., Verdel N. (2001). Activator Solid Support for Metallocene Catalysts in the Polymerization of Olefins, a Process for Preparing Such a Support, and the Corresponding Catalytic System and Polymerization Process. U.S. Patent.

[89] McDaniel M.P., Collins K.S., Eaton A.P., Benham E.A., Jensen M.D., Martin J.L., Hawley G.R. (2002). Organometal Catalyst Compositions. U.S. Patent.

[90] McDaniel M.P., Collins K.S., Smith J.L., Benham E.A., Johnson M.M., Eaton A.P., Jensen M.D., Martin J.L., Hawley G.R. (2002). Organometal Catalyst Compositions. U.S. Patent.

[91] McDaniel M.P., Collins K.S., Hawley G.R., Jensen M.D., Benham E.A., Eaton A.P., Martin J.I., Wittner C.E. (2003). Organometal Compound Catalyst. U.S. Patent.

[92] McDaniel M.P., Benham E.A., Martin S.J., Collins K.S., Smith J.L., Hawley G.R., Wittner C.E., Jensen M.D. (2005). Compositions That Can Produce Polymers. U.S. Patent.

[93] Jensen M.D., Hawley G.R., McDaniel M.P., Crain T., Benham E.A., Martin J.L., Yang Q. (2005). Acidic Activator-Supports and Catalysts for Olefin Polymerization. U.S. Patent.

[94] McDaniel M.P., Yang O., Muninger R.S., Benham E.A., Collins K.S. (2010). Silica-Coated Aluminia Activator-Supports for Metallocene Catalyst Composition. U.S. Patent.

[95] Yang Q., Jensen M.D., Thorn M.G., Jayratne K.C., Crain T.R. (2008). Catalyst for Olefin Polymerization. U.S. Patent.

[96] McDaniel M.P., Kilgore U., Yang Q., Clear K.S. (2016). Methods for Producing Fluorided-Chlorided Silica-Coated Alumina Activator-Supports and Catalyst Systems Containing the Same. U.S. Patent.

[97] Small B.L., Hope K.D., Masino A.P., McDaniel M.P., Buck R.M., Beaulieu W.B., Yang Q., Baralt E.J., Netemeyer E.J., Kreischer B. (2010). Oligomerization of Alpha Olefins Using Metallocene-SSA Catalyst Systems and Use of the Resultant Polyalphaolefins to Prepare Lubricant Blends. U.S. Patent.

[98] McDaniel M.P., Yang Q., Muninger R.S., Benham E.A., Clear K.S. (2019). Silica-Coated Alumina Activator-Supports for Metallocene Catalyst Compositions. U.S. Patent.

[99] Clarembeau M., Pannier G., Paye S. (2015). Activating Supports. U.S. Patent.

[100] Pannier G., Chai C.K. (2015). Polymers. U.S. Patent.

[101] Muron M., Pannier G., Whiteoak C.J., Spitz R., Boisson C. (2015). Activating Supports. U.S. Patent.

[102] Prades F., Boisson C., Spitz R., Razavi A. (2010). Activating Supports for Metallocene Catalysis. U.S. Patent.

[103] Prades F. (2012). Metallocene Catalyst Components Supported on Activating Supports. U.S. Patent.

[104] Pannier G., Boisson C., Spitz R. (2013). Activating Supports with Controlled Distribution of OH Groups. U.S. Patent.

[105] Tisse V.F., Prades F., Briquel R., Boisson C., McKenna T.F.L. (2010). Role of Silica Properties in the Polymerisation of Ethylene Using Supported Metallocene Catalysts. Macromol. Chem. Phys..

[106] Tisse V.F., Boisson C., McKenna T.F.L. (2014). Activation and Deactivation of the Polymerization of Ethylene over *rac*-EtInd_2_ZrCl_2_ and (*n*BuCp)_2_ZrCl_2_ on an Activating Silica Support. Macromol. Chem. Phys..

[107] Prades F., Broyer J.-P., Belaid I., Boyron O., Miserque O., Spitz R., Boisson C. (2013). Borate and MAO Free Activating Supports for Metallocene Complexes. ACS Catal..

[108] Eisen M.S., Marks T.J. (1994). Recent developments in the surface and catalytic chemistry of supported organoactinides. J. Mol. Catal..

[109] Copéret C., Chabanas M., Saint-Arroman R.P., Basset J.-M. (2003). Homogeneous and Heterogeneous Catalysis: Bridging the Gap through Surface Organometallic Chemistry. Angew. Chem. Int. Ed..

[110] Rascón F., Wischert R., Copéret C. (2011). Molecular nature of support effects in single-site heterogeneous catalysts: Silica vs. alumina. Chem. Sci..

[111] Wegener S.L., Marks T.J., Stair P.C. (2012). Design Strategies for the Molecular Level Synthesis of Supported Catalysts. Acc. Chem. Res..

[112] McDaniel M.P., Jensen M.D., Jayaratne K., Collins K.S., Benham E.A., McDaniel N.D., Das P.K., Martin J.L., Yang Q., Thorn M.G., Severn J.R., Chadwick J.C. (2008). Metallocene Activation by Solid Acids. Tailor-Made Polymers: Via Immobilization of Alpha-Olefin Polymerization Catalysts.

[113] Chadwick J.C., Severn J.R., Chadwick J.C. (2008). Catalysts Supported on Magnesium Chloride. Tailor-Made Polymers: Via Immobilization of Alpha-Olefin Polymerization Catalysts.

[114] Hoff R. (2018). Activator Supports for Metallocene and Related Catalysts. Handbook of Transition Metal Polymerization Catalysts.

[115] Kumawat J., Gupta V.K., Vanka K. (2014). The Nature of the Active Site in Ziegler–Natta Olefin Polymerization Catalysis Systems—A Computational Investigation. Eur. J. Inorg. Chem..

[116] Credendino R., Liguori D., Fan Z., Morini G., Cavallo L. (2015). Toward a Unified Model Explaining Heterogeneous Ziegler–Natta Catalysis. ACS Catal..

[117] Potapov A.G. (2017). Titanium-magnesium Ziegler-Natta catalysts: New insight on the active sites precursor. Mol. Catal..

[118] Klaue A., Kruck M., Friederichs N., Bertola F., Wu H., Morbidelli M. (2019). Insight into the Synthesis Process of an Industrial Ziegler–Natta Catalyst. Ind. Eng. Chem. Res..

[119] Zorve P., Linnolahti M. (2018). Adsorption of Titanium Tetrachloride on Magnesium Dichloride Clusters. ACS Omega.

[120] Vittoria A., Meppelder A., Friederichs N., Busico V., Cipullo R. (2017). Demystifying Ziegler–Natta Catalysts: The Origin of Stereoselectivity. ACS Catal..

[121] Correa A., Credendino R., Pater J.T.M., Morini G., Cavallo L. (2012). Theoretical Investigation of Active Sites at the Corners of MgCl_2_ Crystallites in Supported Ziegler–Natta Catalysts. Macromolecules.

[122] Potapov A.G., Terskikh V.V., Bukatov G.D., Zakharov V.A. (1997). ^27^Al NMR MAS study of AlEt_3-*n*_Cl_n_MgCl_2_ systems. J. Mol. Catal. A Chem..

[123] Linnolahti M., Pakkanen T.A., Bazhenova A.S., Denifl P., Leinonen T., Pakkanen A. (2017). Alkylation of titanium tetrachloride on magnesium dichloride in the presence of Lewis bases. J. Catal..

[124] Yu Y., McKenna T.F.L., Boisson C., Lacerda Miranda M.S., Martins O. (2020). Engineering Poly(ethylene-*co*-1-butene) through Modulating the Active Species by Alkylaluminum. ACS Catal..

[125] Ochędzan-Siodłak W., Nowakowska M. (2004). Magnesium chloride modified with organoaluminium compounds as a support of the zirconocene catalyst for ethylene polymerisation. Eur. Polym. J..

[126] Soga K., Uozumi T., Saito M., Shiono T. (1994). Structure of polypropene and poly(ethylene-co-propene) produced with an alumina-supported CpTiCl_3_/common alkylaluminium catalyst system. Macromol. Chem. Phys..

[127] Kang K.K., Oh J.K., Jeong Y.-T., Shiono T., Ikeda T. (1999). Highly active MgCl_2_-supported CpMCl_3_ (M = Ti, Zr) catalysts for ethylene polymerization. Macromol. Rapid Commun..

[128] Goto K., Taniike T., Terano M. (2013). Dual-Active-Site Nature of Magnesium Dichloride—Supported Cyclopentadienyl Titanium Chloride Catalysts Switched by an Activator in Propylene Polymerization. Macromol. Chem. Phys..

[129] Soga K., Kaji E., Uozumi T. (1997). Polymerization of propene with the MgCl_2_-supported dichlorobis (β-diketonato) titanium catalysts combined with methylaluminoxane or an ordinary alkylaluminum. J. Polym. Sci. A Polym. Chem..

[130] Matsui S., Mitani M., Saito J., Tohi Y., Makio H., Matsukawa N., Takagi Y., Tsuru K., Nitabaru M., Nakano T. (2001). A Family of Zirconium Complexes Having Two Phenoxy-Imine Chelate Ligands for Olefin Polymerization. J. Am. Chem. Soc..

[131] Mitani M., Mohri J., Yoshida Y., Saito J., Ishii S., Tsuru K., Matsui S., Furuyama R., Nakano T., Tanaka H. (2002). Living Polymerization of Ethylene Catalyzed by Titanium Complexes Having Fluorine-Containing Phenoxy-Imine Chelate Ligands. J. Am. Chem. Soc..

[132] Mitani M., Furuyama R., Mohri J., Saito J., Ishii S., Terao H., Kashiwa N., Fujita T. (2002). Fluorine- and Trimethylsilyl-Containing Phenoxy-Imine Ti Complex for Highly Syndiotactic Living Polypropylenes with Extremely High Melting Temperatures. J. Am. Chem. Soc..

[133] Mitani M., Furuyama R., Mohri J., Saito J., Ishii S., Terao H., Nakano T., Tanaka H., Fujita T. (2003). Syndiospecific Living Propylene Polymerization Catalyzed by Titanium Complexes Having Fluorine-Containing Phenoxy-Imine Chelate Ligands. J. Am. Chem. Soc..

[134] Makio H., Kashiwa N., Fujita T. (2002). FI Catalysts: A New Family of High Performance Catalysts for Olefin Polymerization. Adv. Synth. Catal..

[135] Makio H., Terao H., Iwashita A., Fujita T. (2011). FI catalysts for olefin polymerization—A comprehensive treatment. Chem. Rev..

[136] Satyanarayana G., Sivaram S. (1993). An unusually stable supported bis(cyclopentadienyl)titanium dichloride-trialkylaluminum catalyst system for ethylene polymerization. Macromolecules.

[137] Nowakowska M., Ochędzan-Siodłak W., Kordowska M. (2000). Metallocene catalysts on complex magnesium support modified by organoaluminium compounds. Polimery.

[138] Cho H.S., Lee W.Y. (2003). Synthesis of inorganic MgCl_2_–alcohol adduct via recrystallization method and its application in supported organometallic catalysts for the polymerization of ethylene with 1-hexene. J. Mol. Catal. A Chem..

[139] Sobota P., Utko J., Lis T., John Ł., Petrus R., Drąg-Jarząbek A. (2016). Unexpected Reactions between Ziegler–Natta Catalyst Components and Structural Characterization of Resulting Intermediates. Inorg. Chem..

[140] Nakayama Y., Saito J., Bando H., Fujita T. (2006). MgCl_2_/R_n_Al(OR)_3−n_: An Excellent Activator/Support for Transition-Metal Complexes for Olefin Polymerization. Chem. Eur. J..

[141] Chung J.S., Cho H.S., Ko Y.G., Lee W.Y. (1999). Preparation of the Ziegler–Natta/metallocene hybrid catalysts on SiO_2_/MgCl_2_ bisupport and ethylene polymerization. J. Mol. Catal. A Chem..

[142] Kissin Y.V., Nowlin T.E., Mink R.I., Brandolini A.J. (2000). A New Cocatalyst for Metallocene Complexes in Olefin Polymerization. Macromolecules.

[143] Carino A.C., Park S.J., Ko Y.S. (2018). Preparation of (*n*-BuCp)_2_ZrCl_2_ Catalyst Supported on SiO_2_/MgCl_2_ Binary Support and its Ethylene-1-hexene Copolymerization. Appl. Chem. Eng..

[144] Scott S.L., Church T.L., Nguyen D.H., Mader E.A., Moran J. (2005). An investigation of catalyst/cocatalyst/support interactions in silica-supported olefin polymerization catalysts based on Cp*TiMe_3_. Top. Catal..

[145] Kumar D., Schumacher K., du Fresne von Hohenesche C., Grün M., Unger K.K. (2001). MCM-41, MCM-48 and related mesoporous adsorbents: Their synthesis and characterisation. Coll. Surf. A Physicochem. Eng. Asp..

[146] Anwander R., Palm C., Groeger O., Engelhardt G. (1998). Formation of Lewis Acidic Support Materials via Chemisorption of Trimethylaluminum on Mesoporous Silicate MCM-41. Organometallics.

[147] Sano T., Niimi T., Miyazaki T., Tsubaki S., Oumi Y., Uozumi T. (2001). Effective activation of metallocene catalyst with AlMCM-41 in propylene polymerization. Catal. Lett..

[148] Li J., DiVerdi J.A., Maciel G.E. (2006). Chemistry of the Silica Surface:  Liquid−Solid Reactions of Silica Gel with Trimethylaluminum. J. Am. Chem. Soc..

[149] Pelletier J., Espinas J., Vu N., Norsic S., Baudouin A., Delevoye L., Trébosc J., Le Roux E., Santini C., Basset J.-M. (2011). A well-defined silica-supported aluminium alkyl through an unprecedented, consecutive two-step protonolysis–alkyl transfer mechanism. Chem. Commun..

[150] Kerber R.N., Kermagoret A., Callens E., Florian P., Massiot D., Lesage A., Copéret C., Delbecq F., Rozanska X., Sautet P. (2012). Nature and Structure of Aluminum Surface Sites Grafted on Silica from a Combination of High-Field Aluminum-27 Solid-State NMR Spectroscopy and First-Principles Calculations. J. Am. Chem. Soc..

[151] Kermagoret A., Kerber R.N., Conley M.P., Callens E., Florian P., Massiot D., Copéret C., Delbecq F., Rozanska X., Sautet P. (2013). Triisobutylaluminum: Bulkier and yet more reactive towards silica surfaces than triethyl or trimethylaluminum. Dalton Trans..

[152] Werghi B., Bendjeriou-Sedjerari A., Sofack-Kreutzer J., Jedidi A., Abou-Hamad E., Cavallo L., Basset J. (2015). Well-defined silica supported aluminum hydride: Another step towards the utopian single site dream?. Chem. Sci..

[153] Kermagoret A., Kerber R.N., Conley M.P., Callens E., Florian P., Massiot D., Delbecq F., Rozanska X., Copéret C., Sautet P. (2014). Chlorodiethylaluminum supported on silica: A dinuclear aluminum surface species with bridging μ2-Cl-ligand as a highly efficient co-catalyst for the Ni-catalyzed dimerization of ethene. J. Catal..

[154] Trueba M., Trasatti S.P. (2005). γ-Alumina as a Support for Catalysts: A Review of Fundamental Aspects. Eur. J. Inorg. Chem..

[155] Motta A., Fragala I.L., Marks T.J. (2008). Links Between Single-Site Heterogeneous and Homogeneous Catalysis. DFT Analysis of Pathways for Organozirconium Catalyst Chemisorptive Activation and Olefin Polymerization on γ-Alumina. J. Am. Chem. Soc..

[156] Stuart N.M., Sohlberg K. (2021). The Microstructure of γ-Alumina. Energies.

[157] Luo Z. (2021). Structure of boehmite-derived γ-alumina and its transformation mechanism revealed by electron crystallography. Acta Cryst. B.

[158] Joubert J., Delbecq F., Thieuleux C., Taoufik M., Blanc F., Copéret C., Thivolle-Cazat J., Basset J.-M., Sautet P. (2007). Synthesis, Characterization, and Catalytic Properties of γ-Al_2_O_3_-Supported Zirconium Hydrides through a Combined Use of Surface Organometallic Chemistry and Periodic Calculations. Organometallics.

[159] Delgado M., Delbecq F., Santini C.C., Lefebvre F., Norsic S., Putaj P., Sautet P., Basset J.-M. (2012). Evolution of Structure and of Grafting Properties of γ-Alumina with Pretreatment Temperature. J. Phys. Chem. C.

[160] Wischert R., Laurent P., Copéret C., Delbecq F., Sautet P. (2012). γ-Alumina: The Essential and Unexpected Role of Water for the Structure, Stability, and Reactivity of “Defect” Sites. J. Am. Chem. Soc..

[161] Wischert R., Florian P., Copéret C., Massiot D., Sautet P. (2014). Visibility of Al Surface Sites of γ-Alumina: A Combined Computational and Experimental Point of View. J. Phys. Chem. C.

[162] Joubert J., Delbecq F., Sautet P., Le Roux E., Taoufik M., Thieuleux C., Blanc F., Copéret C., Thivolle-Cazat J., Basset J.-M. (2006). Molecular Understanding of Alumina Supported Single-Site Catalysts by a Combination of Experiment and Theory. J. Am. Chem. Soc..

[163] Joubert J., Delbecq F., Copéret C., Basset J.-M., Sautet P. (2008). Gamma-alumina: An Active Support to Obtain Immobilized Electron Poor Zr Complexes. Top. Catal..

[164] Mason A.H., Motta A., Das A., Ma Q., Bedzyk M.J., Kratish Y., Marks T.J. (2022). Rapid atom-efficient polyolefin plastics hydrogenolysis mediated by a well-defined single-site electrophilic/cationic organo-zirconium catalyst. Nat. Commun..

[165] Delgado M., Santini C.C., Delbecq F., Wischert R., Le Guennic B., Tosin G., Spitz R., Basset J.-M., Sautet P. (2010). Alumina as a Simultaneous Support and Co Catalyst: Cationic Hafnium Complex Evidenced by Experimental and DFT Analyses. J. Phys. Chem. C.

[166] Mazoyer E., Trébosc J., Baudouin A., Boyron O., Pelletier J., Basset J.-M., Vitorino M.J., Nicholas C.P., Gauvin R.M., Taoufik M. (2010). Heteronuclear NMR Correlations To Probe the Local Structure of Catalytically Active Surface Aluminum Hydride Species on γ-Alumina. Angew. Chem..

[167] Ikenaga K., Chen S., Ohshima M., Kurokawa H., Miura H. (2007). Ethylene polymerization over zirconocenes supported on alumina- and titania-based acidic oxides. Catal. Commun..

[168] Ashuiev A., Allouche F., Wili N., Searles K., Klose D., Copéret C., Jeschke G. (2021). Molecular and supported Ti(III)-alkyls: Efficient ethylene polymerization driven by the π-character of metal–carbon bonds and back donation from a singly occupied molecular orbital. Chem. Sci..

[169] Chizallet C., Raybaud P. (2010). Acidity of Amorphous Silica–Alumina: From Coordination Promotion of Lewis Sites to Proton Transfer. ChemPhysChem.

[170] Weiss K., Wirth-Pfeifer C., Hofmann M., Botzenhardt S., Lang H., Bruning K., Meichel E. (2002). Polymerisation of ethylene or propylene with heterogeneous metallocene catalysts on clay minerals. J. Mol. Catal. A Chem..

[171] Weiss K., Botzenhardt S., Hofmann M., Kaminsky W. (2008). Ziegler-Natta and Metallocene Polymerisation of Olefins with Heterogeneous Catalysts. Metalorganic Catalysts for Synthesis and Polymerization.

[172] Nakano H., Takahashi T., Uchino H., Tayano T., Sugano T. (2006). 4-Polymerization Behavior with Metallocene Catalyst Supported by Clay Mineral Activator. Stud. Surf. Sci. Catal..

[173] Sun T., Garcés J.M. (2003). Acidic lamellar aerogel nanoplate activated olefin polymerization with metallocene catalysts. Catal. Commun..

[174] Shih K.-Y., Denton D.A., Carney M.J. (2003). Metallocene and Constrained Geometry Catalyst Systems Employing Agglomerated Metal Oxide/Clay Support-Activator and Method of Their Preparation. U.S. Patent.

[175] Tabernero V., Camejo C., Terreros P., Alba M.D., Cuenca T. (2010). Silicoaluminates as “Support Activator” Systems in Olefin Polymerization Processes. Materials.

[176] Yamamoto K. (2009). Olefin polymerizations with zirconocene supported on SiO_2_ modified by MgO, NaOH and LiOH. Appl. Catal. A Gen..

[177] Higuchi K., Onaka M., Izumi Y. (1993). Solid Acid and Base-Catalyzed Cyanosilylation of Carbonyl Compounds with Cyanotrimethylsilane. Bull. Chem. Soc. Jpn..

[178] Corma A. (1995). Inorganic Solid Acids and Their Use in Acid-Catalyzed Hydrocarbon Reactions. Chem. Rev..

[179] Williams L.A., Guo N., Motta A., Delferro M., Fragalà I.L., Miller J.T., Marks T.J. (2012). Surface structural-chemical characterization of a single-site d^0^ heterogeneous arene hydrogenation catalyst having 100% active sites. Proc. Natl. Acad. Sci. USA.

[180] Gu W., Stalzer M.M., Nicholas C.P., Bhattacharyya A., Motta A., Gallagher J.R., Zhang G., Miller J.T., Kobayashi T., Pruski M. (2015). Benzene Selectivity in Competitive Arene Hydrogenation: Effects of Single-Site Catalyst···Acidic Oxide Surface Binding Geometry. J. Am. Chem. Soc..

[181] Stalzer M.M., Nicholas C.P., Bhattacharyya A., Motta A., Delferro M., Marks T.J. (2016). Single-Face/All-cis Arene Hydrogenation by a Supported Single-Site d^0^ Organozirconium Catalyst. Angew. Chem. Int. Ed..

[182] Li X., Nagaoka K., Simon L.J., Olindo R., Lercher J.A., Hofmann A., Sauer J. (2005). Oxidative Activation of *n*-Butane on Sulfated Zirconia. J. Am. Chem. Soc..

[183] Liu J., Liu N., Ren K., Shi L., Meng X. (2017). Sulfated Zirconia Synthesized in a One Step Solvent-Free Method for Removal of Olefins from Aromatics. Ind. Eng. Chem. Res..

[184] Hofmann A., Sauer J. (2004). Surface Structure of Hydroxylated and Sulfated Zirconia. A Periodic Density-Functional Study. J. Phys. Chem. B.

[185] Benaïssa M., Santiesteban J.G., Díaz G., Chang C.D., José-Yacamán M. (1996). Interaction of Sulfate Groups with the Surface of Zirconia: An HRTEM Characterization Study. J. Catal..

[186] Zhang J., Motta A., Gao Y., Stalzer M.M., Delferro M., Liu B., Lohr T.L., Marks T.J. (2018). Cationic Pyridylamido Adsorbate on Brønsted Acidic Sulfated Zirconia: A Molecular Supported Organohafnium Catalyst for Olefin Homo- and Co-Polymerization. ACS Catal..

[187] Domski G.J., Lobkovsky E.B., Coates G.W. (2007). Polymerization of α-Olefins with Pyridylamidohafnium Catalysts: Living Behavior and Unexpected Isoselectivity from a C_s_-Symmetric Catalyst Precursor. Macromolecules.

[188] Wei J., Zhang W., Wickham R., Sita L.R. (2010). Programmable Modulation of Co-monomer Relative Reactivities for Living Coordination Polymerization through Reversible Chain Transfer between “Tight” and “Loose” Ion Pairs. Angew. Chem. Int. Ed..

[189] Froese R.D.J., Jazdzewski B.A., Klosin J., Kuhlman R.L., Theriault C.N., Welsh D.M., Abboud K.A. (2011). Imino-Amido Hf and Zr Complexes: Synthesis, Isomerization, and Olefin Polymerization. Organometallics.

[190] Figueroa R., Froese R.D., He Y., Klosin J., Theriault C.N., Abboud K.A. (2011). Synthesis of Imino-Enamido Hafnium and Zirconium Complexes: A New Family of Olefin Polymerization Catalysts with Ultrahigh-Molecular-Weight Capabilities. Organometallics.

[191] Klosin J., Fontaine P.P., Figueroa R. (2015). Development of Group IV Molecular Catalysts for High Temperature Ethylene-α-Olefin Copolymerization Reactions. Acc. Chem. Res..

[192] Baier M.C., Zuideveld M.A., Mecking S. (2014). Post-Metallocenes in the Industrial Production of Polyolefins. Angew. Chem. Int. Ed..

[193] Zuccaccia C., Macchioni A., Busico V., Cipullo R., Talarico G., Alfano F., Boone H.W., Frazier K.A., Hustad P.D., Stevens J.C. (2008). Intra- and Intermolecular NMR Studies on the Activation of Arylcyclometallated Hafnium Pyridyl-Amido Olefin Polymerization Precatalysts. J. Am. Chem. Soc..

[194] Zhang J., Mason A.H., Motta A., Cesar L.G., Kratish Y., Lohr T.L., Miller J.T., Gao Y., Marks T.J. (2021). Surface *vs* Homogeneous Organo-Hafnium Catalyst Ion-Pairing and Ligand Effects on Ethylene Homo- and Copolymerizations. ACS Catal..

[195] Williams L.A., Marks T.J. (2011). Synthesis, Characterization, and Heterogeneous Catalytic Implementation of Sulfated Alumina Nanoparticles. Arene Hydrogenation and Olefin Polymerization Properties of Supported Organozirconium Complexes. ACS Catal..

[196] Zhang J., Mason A.H., Wang Y., Motta A., Kobayashi T., Pruski M., Gao Y., Marks T.J. (2021). Beyond the Active Site. Cp*ZrMe_3_/Sulfated Alumina-Catalyzed Olefin Polymerization Tacticity via Catalyst Surface Ion-Pairing. ChemCatChem.

[197] Williams L.A., Marks T.J. (2009). Chemisorption Pathways and Catalytic Olefin Polymerization Properties of Group 4 Mono- and Binuclear Constrained Geometry Complexes on Highly Acidic Sulfated Alumina. Organometallics.

[198] McInnis J.P., Delferro M., Marks T.J. (2014). Multinuclear Group 4 Catalysis: Olefin Polymerization Pathways Modified by Strong Metal-Metal Cooperative Effects. Acc. Chem. Res..

[199] Delferro M., Marks T.J. (2011). Multinuclear Olefin Polymerization Catalysts. Chem. Rev..

[200] Liu S., Invergo A.M., McInns J.P., Mouat A.R., Motta A., Lohr T.L., Delferro M., Marks T.J. (2017). Distinctive Stereochemically Linked Cooperative Effects in Bimetallic Titanium Olefin Polymerization Catalysts. Organometallics.

[201] Yang Q., Jensen M.D., Martin J.L., Thorn M.G., McDaniel M.P., Yu Y., Rohlfing D.C. (2007). Polymerization Catalysts for Producing High Molecular Weight Polymers with Low Levels of Long Chain Branching. U.S. Patent.

[202] Yang Q., McDaniel M.P., Martin J.L., Ding E., Rohlfing D.C., Crain T.R. (2012). Catalyst Systems and Methods of Making and Using Same. U.S. Patent.

[203] McDaniel M.P., Yang Q., Crain T.R. (2015). Processes for Preparing Metallocene-Based Catalyst Systems. U.S. Patent.

[204] Clark K.M., Yang Q., Glass G.L. (2016). Processes for Preparing Solid Metallocene-Based Catalyst Systems. U.S. Patent.

[205] Iacono S.T., Jennings A.R. (2019). Recent Studies on Fluorinated Silica Nanometer-Sized Particles. Nanomaterials.

[206] Kolditz L., Calov U., Kauschka G., Schmidt W. (1977). Herstellung von fluorreichen Verbindungen der Reihe C_2_Cl_n_F_6−n_ in heterogener Katalyse. Z. Anorg. Allg. Chem..

[207] Scokart P.O., Selim S.A., Damon J.P., Rouxhet P.G. (1979). The chemistry and surface chemistry of fluorinated alumina. J. Colloid Interface Sci..

[208] Krahl T., Kemnitz E. (2017). Aluminium fluoride—The strongest solid Lewis acid: Structure and reactivity. Catal. Sci. Technol..

[209] Becker C., Braun T., Paulus B. (2021). Theoretical Study on the Lewis Acidity of the Pristine AlF_3_ and Cl-Doped α-AlF_3_ Surfaces. Catalysts.

[210] Kemnitz E., Groß U., Rüdiger S., Shekar C.S. (2003). Amorphous Metal Fluorides with Extraordinary High Surface Areas. Angew. Chem. Int. Ed..

[211] Calvo B., Marshall C.P., Krahl T., Kröhnert J., Trunschke A., Scholz G., Braun T., Kemnitz E. (2018). Comparative study of the strongest solid Lewis acids known: ACF and HS-AlF_3_. Dalton Trans..

[212] Ahrem L., Wolf J., Scholz G., Kemnitz E. (2018). A novel fluoride-doped aluminium oxide catalyst with tunable Brønsted and Lewis acidity. Catal. Sci. Technol..

[213] Fischer L., Harlé V., Kasztelan S., d’Espinose de la Caillerie J.-B. (2000). Identification of fluorine sites at the surface of fluorinated γ-alumina by two-dimensional MAS NMR. Solid State Nucl. Magn. Reson..

[214] Panchenko V.N., Danilova G., Zakharov V.A., Semikolenova N.V., Paukshtis E.A. (2017). Effect of the acid–base properties of the support on the catalytic activity of ethylene polymerization using supported catalysts composed of Cp_2_ZrX_2_ (X = Cl, Me) and Al_2_O_3_. Reac. Kinet. Mech. Catal..

[215] Corma A., Fornés V., Ortega E. (1985). The nature of acid sites on fluorinated γ-Al_2_O_3_. J. Catal..

[216] McDaniel M.P., Klendworth D.D., Johnson M.M. (1992). Fluorided Aluminas, Catalysts, and Polymerization Processes. U.S. Patent.

[217] Prades F., Spitz R., Boisson C., Sirol S., Razavi A. (2013). Transition Metal Complexes Supported on Activating Support. U.S. Patent.

[218] Daniell W., Schubert U., Glöckler R., Meyer A., Noweck K., Knözinger H. (2000). Enhanced surface acidity in mixed alumina–silicas: A low-temperature FTIR study. Appl. Catal. A Gen..

[219] Ding E., Yang Q., Guatney L.W., Greco J.F. (2017). Ziegler-Natta—Metallocene Dual Catalyst Systems with Activator-Supports. U.S. Patent.

[220] Ding E., Yang Q., Guatney L.W., Greco J.F. (2017). Heterogeneous Ziegler-Natta Catalysts with Fluorided Silica-Coated Alumina. U.S. Patent.

[221] Terano M., Soga H., Kimura K. (1989). Catalyst for Polymerization of Olefins. U.S. Patent.

[222] Nifant’ev I.E., Vinogradov A.A., Vinogradov A.A., Bagrov V.V., Churakov A.V., Minyaev M.E., Kiselev A.V., Salakhov I.I., Ivchenko P.V. (2022). A competetive way to low-viscosity PAO base stocks via heterocene-catalyzed oligomerization of dec-1-ene. Mol. Catal..

[223] Nifant’ev I.E., Vinogradov A.A., Vinogradov A.A., Bagrov V.V., Kiselev A.V., Minyaev M.E., Samurganova T.I., Ivchenko P.V. (2023). Heterocene Catalysts and Reaction Temperature Gradient in Dec-1-ene Oligomerization for the Production of Low Viscosity PAO Base Stocks. Ind. Eng. Chem. Res..

[224] Soga K., Uozumi T., Kishi N. (1995). A metallocene catalyst system for olefin polymerization using a heteropolyacid as the counter-anion. Macromol. Rapid Commun..

[225] McDaniel M.P., Collins K.S., Johnson M.M., Smith J.L., Benham E.A., Hawley G.R., Wittner C.E., Jensen M.D. (2000). Compositions That Can Produce Polymers. U.S. Patent.

[226] McDaniel M.P., Kimble J.B., Collins K.S., Benham E.A., Jensen M.D., Hawley G.R., Martin J.L. (2002). Organometal Catalyst Compositions. U.S. Patent.

[227] McDaniel M.P., Collins K.S., Eaton A.P., Benham E.A., Jensen M.D., Martin J.L., Hawley G.R. (2003). Organometal Catalyst Compositions. U.S. Patent.

[228] Kurokawa H., Morita S., Matsuda M., Suzuki H., Ohshima M., Miura H. (2009). Polymerization of ethylene using zirconocenes supported on swellable cation-exchanged fluorotetrasilicic mica. Appl. Catal. A Gen..

[229] Ward D.G. (1999). Halogenated Supports and Supported Activators. U.S. Patent.

[230] Bando H., Nakayama Y., Sonobe Y., Fujita T. (2003). Bis(Phenoxy-Imine) Zr Complexes/Et_3_Al/Heteropoly Compound Catalyst Systems for Ethylene Polymerization. Macromol. Rapid. Commun..

[231] Hicks J.C., Mullis B.A., Jones C.W. (2007). Sulfonic Acid Functionalized SBA-15 Silica as a Methylaluminoxane-Free Cocatalyst/Support for Ethylene Polymerization. J. Am. Chem. Soc..

[232] Alvaro M., Corma A., Das D., Fornésa V., García H. (2004). Single-step preparation and catalytic activity of mesoporous MCM-41 and SBA-15 silicas functionalized with perfluoroalkylsulfonic acid groups analogous to Nafion. Chem. Commun..

[233] Culver D.B., Venkatesh A., Huynh W., Rossini A.J., Conley M.P. (2020). Al(OR^F^)_3_ (R^F^ = C(CF_3_)_3_) activated silica: A well-defined weakly coordinating surface anion. Chem. Sci..

[234] Samudrala K.K., Huynh W., Dorn R.W., Rossini A.J., Conley M.P. (2022). Formation of a Strong Heterogeneous Aluminum Lewis Acid on Silica. Angew. Chem. Int. Ed..

[235] Kusoglu A., Weber A.Z. (2017). New Insights into Perfluorinated Sulfonic-Acid Ionomers. Chem. Rev..

[236] Tasgiro T., Ueda T. (1997). Catalyst Olefin Polymerization and Process for Olefin Polymerization Using the Same. U.S. Patent.

[237] Pârvulescu V.I., Hardacre C. (2007). Catalysis in Ionic Liquids. Chem. Rev..

[238] Hogg J.M., Ferrer-Ugalde A., Coleman F., Swadźba-Kwaśny M. (2019). Borenium Ionic Liquids as Alternative to BF_3_ in Polyalphaolefins (PAOs) Synthesis. ACS Sustain. Chem. Eng..

[239] Fehér C., Tomasek S., Hancsók J., Skoda-Földes R. (2018). Oligomerization of light olefins in the presence of a supported Brønsted acidic ionic liquid catalyst. Appl. Catal. B Environ..

[240] Mashayekhi M., Talebi S., Sadjadi S., Bahri-Laleh N. (2021). Production of polyalfaolefin-based lubricants using new (poly)ionic liquid/AlCl_3_ catalysts as environmentally friendly alternatives to commercial AlCl_3_ route. Appl. Catal. A Gen..

[241] Tabrizi M., Bahri-Laleh N., Sadjadi S., Nekoomanesh-Haghighi M. (2021). The effect of ionic liquid containing AlCl_3_ catalytic systems on the microstructure and properties of polyalphaolefin based lubricants. J. Mol. Liq..

[242] Karimi S., Sadjadi S., Bahri-Laleh N., Nekoomanesh-Haghighi M. (2021). New less-toxic halloysite-supported ionic liquid/AlCl_3_ oligomerization catalysts: A comparative study on the effects of various ionic liquids on the properties of polyalphaolefins. Mol. Catal..

[243] Carlin R.T., Wilkes J.S. (1990). Complexation of Cp_2_MCl_2_ in a chloroaluminate molten salt: Relevance to homogeneous Ziegler-Natta catalysis. J. Mol. Catal..

[244] Deeth R.J., Jenkins H.D.B. (1997). A Density Functional and Thermochemical Study of M−X Bond Lengths and Energies in [MX_6_]^2−^ Complexes:  LDA versus Becke88/Perdew86 Gradient-Corrected Functionals. J. Phys. Chem. A.

[245] Ochędzan-Siodłak W., Sacher-Majewska B. (2007). Biphasic ethylene polymerisation using ionic liquid over a titanocene catalyst activated by an alkyl aluminium compound. Eur. Polym. J..

[246] Ochędzan-Siodłak W., Pawelska P. (2008). Chloroaluminate ionic liquids as a medium of titanocene catalyst activated by alkyl aluminum compounds for ethylene polymerization. Polymery.

[247] Ochędzan-Siodłak W., Dziubek K., Czaja K. (2009). Comparison of imidazolium and pyridinium ionic liquids as the media for biphasic ethylene polymerization in the presence of titanocene catalyst. Polymery.

[248] Ochędzan-Siodłak W., Dziubek K., Czaja K. (2013). Effect of immobilization of titanocene catalyst in aralkyl imidazolium chloroaluminate media on performance of biphasic ethylene polymerization and polyethylene properties. Polym. Bull..

[249] Ochędzan-Siodłak W., Dziubek K. (2014). Metallocenes and post-metallocenes immobilized on ionic liquid-modified silica as catalysts for polymerization of ethylene. Appl. Catal. A Gen..

[250] Nifant’ev I.E., Salakhov I.I., Ivchenko P.V. (2022). Transition Metal–(μ-Cl)–Aluminum Bonding in α-Olefin and Diene Chemistry. Molecules.

[251] Jalali A., Nekoomanehs-Haghighi M., Dehghani S., Bahri-Laleh N. (2020). Effect of metal type on the metallocene-catalyzed oligomerization of 1-hexene and 1-octene to produce polyα-olefin-based synthetic lubricants. Appl. Organomet. Chem..

[252] Hanifpour A., Bahri-Laleh N., Nekoomanesh-Haghighi M., Poater A. (2021). 1-Decene oligomerization by new complexes bearing diamine-diphenolates ligands: Effect of ligand structure. Appl. Organomet. Chem..

[253] Hanifpour A., Gargari M.H., Darounkola M.R.R., Kalantari Z., Bahri-Laleh N. (2021). Kinetic and microstructural studies of Cp_2_ZrCl_2_ and Cp_2_HfCl_2_-catalyzed oligomerization of higher α-olefins in mPAO oil base stocks production. Polyolefins J..

[254] Nifant’ev I.E., Vinogradov A.A., Vinogradov A.A., Minyaev M.E., Bagrov V.V., Salakhov I.I., Shaidullin N.M., Chalykh A.E., Shapagin A.V., Ivchenko P.V. (2023). Heterocene-catalyzed ethylene/oct-1-ene copolymerization under MAO-free and low-MAO conditions: The synthesis of highly statistical copolymers and their use in blending with HDPE. Polymer.

